# Antibacterial Barbituric Acid Analogues Inspired from Natural 3-Acyltetramic Acids; Synthesis, Tautomerism and Structure and Physicochemical Property-Antibacterial Activity Relationships

**DOI:** 10.3390/molecules20033582

**Published:** 2015-02-20

**Authors:** Yong-Chul Jeong, Mark G. Moloney

**Affiliations:** Chemistry Research Laboratory, Mansfield Rd, University of Oxford, OX1 3TA, UK; E-Mail: ycjchem@gmail.com

**Keywords:** barbiturates, antibacterial, synthesis, tautomerism

## Abstract

The synthesis, tautomerism and antibacterial activity of novel barbiturates is reported. In particular, 3-acyl and 3-carboxamidobarbiturates exhibited antibacterial activity, against susceptible and some resistant Gram-positive strains of particular interest is that these systems possess amenable molecular weight, rotatable bonds and number of proton-donors/acceptors for drug design as well as less lipophilic character, with physicochemical properties and ionic states that are similar to current antibiotic agents for oral and injectable use. Unfortunately, the reduction of plasma protein affinity by the barbituric core is not sufficient to achieve activity *in vivo*. Further optimization to reduce plasma protein affinity and/or elevate antibiotic potency is therefore required, but we believe that these systems offer unusual opportunities for antibiotic drug discovery.

## 1. Introduction

**Figure 1:**
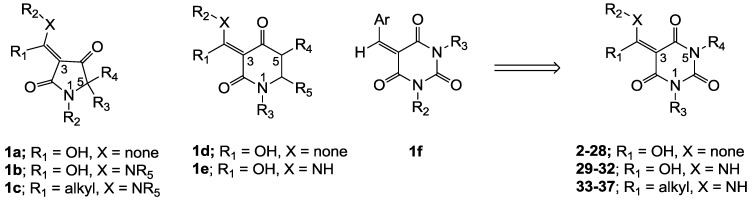
Optimization of core scaffold.

The use of natural products as leads for antibacterial drug discovery is enjoying a resurgence of interest, forced by the failure of existing drug discovery strategies, the particular requirements of antibacterial therapies, the emergence of virulent bacterial strains and the paucity of new development candidates working their way through the drug development pipeline [1,2]. In this regard, the tetramic acid scaffold (especially with a 3-acyl side chain moiety) [3,4,5,6,7,8] is of particular interest, since naturally occurring 3-acyltetramic acids such as reutericyclin (bacterial membrane disruption) [5], streptolydigin (bacterial RNA polymerase (RNAP) inhibitor) [6], kibdelomycin (bacterial type II topoisomerase inhibitor) [7] and signermycin B (the dimerization domain of histidine kinase WalK inhibitor) [8] all exhibit antibacterial activity with novel modes of action. Aiming to develop both the biological activity and bioavailability of these systems, methodology for the modification of ring substituents in natural 3-acyltetramic acids (R_1_-R_4_ in **1a**) [9,10,11,12,13,14] as well as the replacement of the 3-acyl side chain group by 3-carboxamide (**1b**) [13,15,16,17,18] and 3-enamine functionalities (**1c**, Figure 1) [13,19] has been reported. These investigations revealed that 3-acyl **1a** and 3-carboxamide **1b** substitutions can impart good antibacterial activity, resulting from novel modes of action including bacterial membrane disruption, inhibition of bacterial RNAP or undecaprenyl pyrophosphate synthase (UPPS), while 3-enamine **1c** exhibits much weaker antibacterial activity and without a clear mode of action. Furthermore, it has been found that the 5-membered tetramic acid core scaffold may also be replaced by the 6-membered piperidine-2,4-dione unit with 3-acyl (**1d**) [14] and 3-carboxamide (**1e**) [16,18] pendant functionality, and that these systems show similar antibacterial activity and mode of action compared to tetramates **1a**,**b**.

Although 3-acyl and 3-carboxamide tetramate analogues reported in our previous papers [13,14,15] showed good antibacterial activity, novel modes of action and acceptable toxicity, their high plasma protein binding (PPB) affinity interfered with further biological study *in vivo* for antibiotic drug discovery. In order to overcome this PPB affinity, we decided to conduct further structural optimization of the core scaffold, by moving from the tetramic acid to the 6-membered barbituric acid system. This scaffold possesses similar chemical structure compared to tetramic acids and piperidine-2,4-diones (especially around the C(3) position), but importantly has additional polar functional groups at the 5- and 6-positions, and we expected that these groups might help to reduce PPB affinity. Further validation of this proposal comes from the fact that arylidene barbituric acids **1f** are reported to have mild antibacterial activity [20,21,22,23]. In this paper, with the inspiration from related analogues **1a**–**f**, novel barbituric acids **2**–**28** (with 3-acyl), **29**–**32** (with 3-carboxamide) and **33**–**37** (with 3-enamine) have been prepared and their tautomeric behavior, antibacterial activity and structure-activity relationships (SARs) have been studied. Furthermore, in order to understand trends of biological activity for further drug optimization, their physicochemical property-activity relationships have been investigated and compared with the tetramic acids reported in our previous papers [13,14,15,19] as well as clinical antibiotics. To the best of our knowledge, the antibacterial activity of 3-acylbarbituric acids with only limited functionality has been reported in the literature [20,21], and the 3-carboxamide and the 3-enamine analogues are as yet completely unreported.

## 2. Result and Discussion

### 2.1. Synthesis

**Scheme 1:**
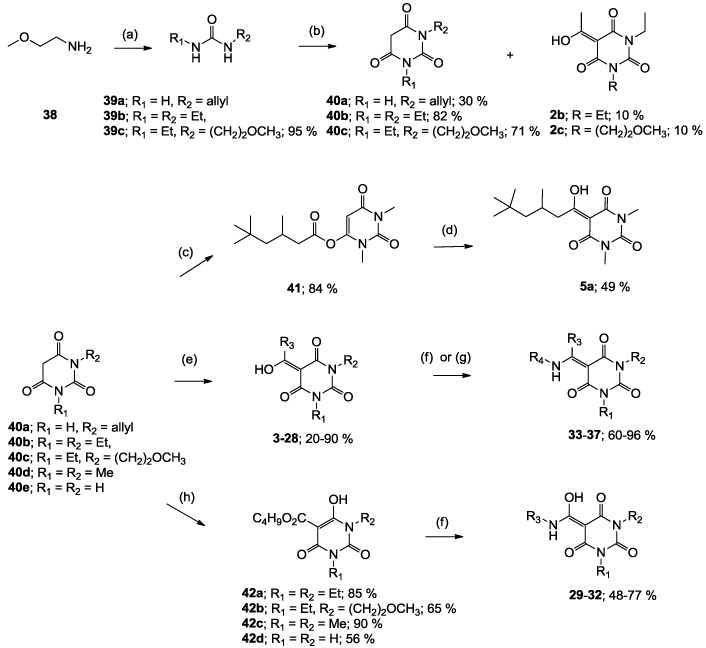
Synthesis of barbituric acid analogues. *Reaction conditions*; (a) ethyl isocyanate (1.0 eq), CH_2_Cl_2_, 0 °C; (b) malonic acid (1.0 eq), acetic acid, acetic anhydride, 60–90 °C; (c) 3,5,5-trimethylhexanoyl chloride (1.1 eq), triethylamine (1.2 eq), CH_2_Cl_2_, r.t.; (d) DMAP (1.2 eq), CH_2_Cl_2_, r.t.; (e) R_3_CO_2_H (1.1 eq), DCC (1.1 eq), DMAP (1.2 eq), CH_2_Cl_2_, r.t.; (f) RNH_2_ (1.0 eq), toluene, reflux; (g) RNH_2_ (1.1 eq), CH_3_OH, reflux; (h) butyl chloroformate (1.2 eq), DMAP (2.2 eq), CH_2_Cl_2_, r.t.; Abbreviation; DCC; *N*,*N*′-dicyclohexylcarbodiimide, DMAP; 4-(dimethylamino)pyridine.

The starting barbituric templates **40a**–**c** were prepared by known methods, while templates **40d**,**e** were commercially available (Scheme 1). Thus, urea **39a**–**c** was condensed with malonic acid in the presence of acetic acid and acetic anhydride to provide barbituric acids **40a**–**c**, respectively [24]. *N*-Disubstituted **40b**,**c**, along with 3-acetyl **2b**,**c** [20,25] as minor products, respectively, could be purified by flash column chromatography, while *N*-monosubstituted **40a** was best obtained by precipitation in ethyl acetate solution. For this reaction, urea **39c** was efficiently obtained from amine **38** and ethyl isocyanate [20,26], while ureas **39a**,**b** were commercially available.

With templates **40a**–**e** in hand, the synthesis of 3-acyl, 3-carboxamide and 3-enamine tetramic acids using recently reported approaches were successfully applied to the synthesis of the corresponding barbituric acid analogues [13,14,15,19,27]. 3-Acyl analogues **3**–**28** (with the exception of **5a**) were prepared via direct 3-acylation of templates **40a**–**e** with the required carboxylic acid promoted by 1.1 equivalent of DCC and 1.2 equivalent of DMAP, while stepwise 3-acylation via *O*-acylation using the acid chloride in the presence of triethylamine followed by acyl migration promoted by DMAP (1.2 equivalent) gave analogue **5a**. Although other synthetic methods for 3-acylbarbituric acids have been reported [20,21,28,29,30], this direct acylation approach provides efficient access to systems with a wide variety of substituents at the acyl group. Furthermore, it is also applicable to *N-*unsubstituted, -mono and di-substituted barbituric acids. 3-Enamines **33**–**37** were prepared by reaction of the corresponding 3-acyl analogue with an amine in refluxing toluene [25,31,32,33]. In the case of compound **36**, methanol instead of toluene was required as solvent. Alternatively, 3-alkoxycarbonyl barbiturates **42a**–**d** needed as starting materials for 3-carboxamides **29**–**32** were conveniently prepared from the corresponding barbituric acids by treatment with butyl chloroformate in the presence of 1.2 equivalents of DMAP. Conventional direct amine exchange of the 3-alkoxycarbonlys in toluene allowed preparation of 3-carboxamides **29**–**32**. To the best of our knowledge, this is the first example of the preparation of 3-carboxamidobarbituric acids.

### 2.2. Tautomerism

**Figure 2:**
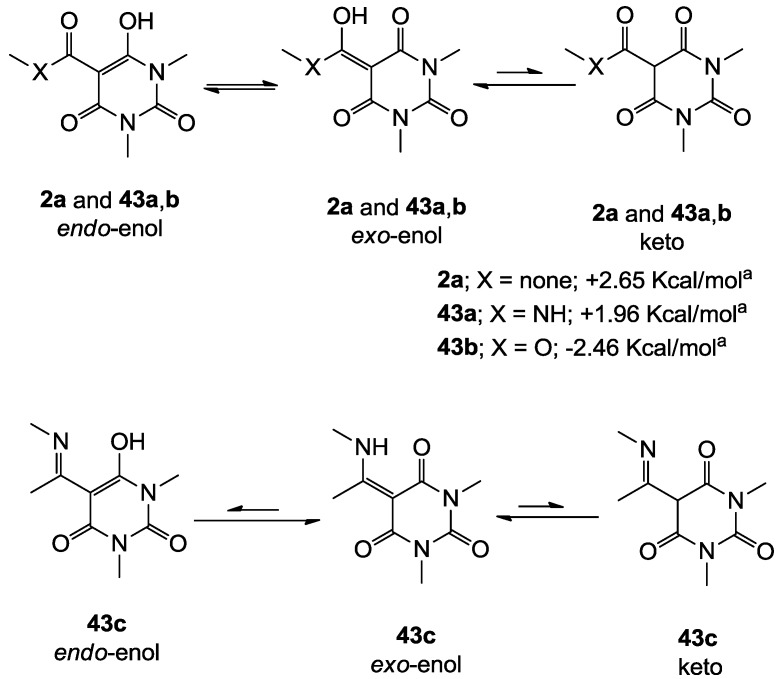
Tautomeric behavior of barbituric acid analogues; the energy difference between *endo*- and *exo*-enol tautomers (∆E = E*_endo_* − E*_exo_*) was calculated by using DFT B3LYP (6-31G*) in Spartan 02.

Similar to tetramic acid derivatives [14,15,19,27], 3-acyl, 3-carboxamide, 3-alkoxycarbonyl and 3-enamine barbituric acids can exist as *endo*- and *exo*-enol and keto tautomers in solution (Figure 2). In the case of barbiturates derived from symmetrical barbituric acids **40b**,**d**,**e**, one set of peaks in their NMR spectra was observed, while in the case of asymmetric barbituric acids **40a**,**c**, split signals (rather than two sets from two tautomeric isomers) were observed. The enol tautomer was assigned from the chemical shift of the C(3)-carbon (80–90 ppm for sp^2^ carbon) as well as the absence of the H(3)-proton signal. The observation of one set of signals for *endo*- and *exo*-enol tautomers supports the fact that equilibration between *endo*- and *exo*-enol tautomers is fast on the NMR time scale, resulting in coalescence of the peaks of the two enol tautomers.

In order to identify the favored enol-form, the ground state energies of simplified analogues, 3-acyl **2a**, 3-carboxamide **43a**, 3-alkoxycarbonyl **43b** and 3-enamine **43c** in *endo*- and *exo*-enol tautomers were calculated (Figure 2). The *exo*-enol form of 3-acyl **2a** and *endo*-enol form of 3-alkoxycarbonyl **43b** were found to be more stable than the alternative enol form, and these results are similar to the favoured tautomer of tetramic acids [15,27]. In contrast, 3-carboxamidobarbiturate **43a** favours the *exo*-enol tautomer, while the corresponding 3-carboxamidotetramate favours the *endo*-enol tautomer [15]. In the case of 3-enamine **43c**, the ground state energy of the *endo*-enol tautomer is much less stable than the *exo*-enol tautomer (compare the geometry of 3-enamine **43c** between *endo*- and *exo*-enol tautomers in Supplementary Figure 1g,h in the Supporting Information). From this computational result, 3-acyl, 3-carboxamide and 3-enamine barbituric acids all preferentially exist as *exo*-enol tautomers, and 3-alkoxycarbonyl as *endo*-enol tautomers. In addition, HMBC NMR spectra of representative analogues were acquired and the correlations for the main ring were established (Supplementary Figure 2 in Supporting Information). In this assignment, the free carbonyl (around 160 ppm) and hydrogen-bonded carbonyl (165–170 ppm) on C(2) and (C4) were readily identified.

### 2.3. Antibacterial Activity

**Figure 3:**
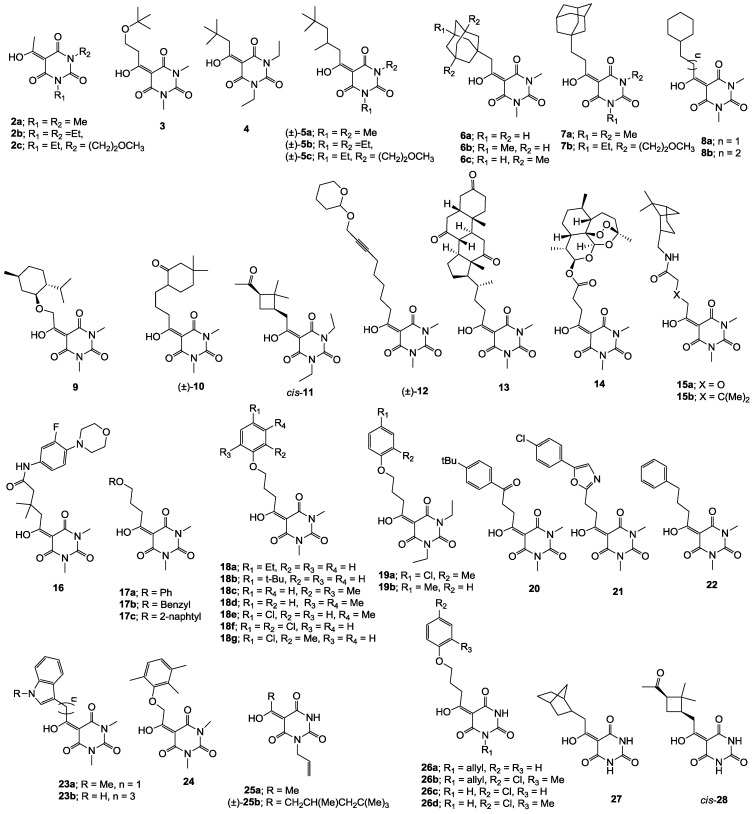
3-Acylbarbituric acids (50 analogues).

**Figure 4:**
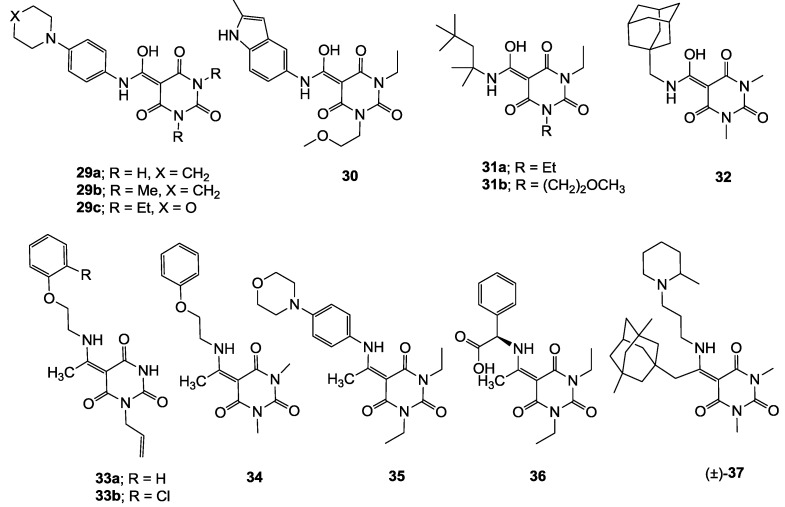
3-Carboxamides (7 analogues) and 3-enamine (6 analogues) barbituric acids.

Minimum inhibition concentration (MIC) values for the *in vitro in vitro* antibacterial activity of 73 barbiturates was determined (shown in Table 1) against Gram-positive bacteria such as *Staphylococcus aureus* (methicillin sensitive S1, vancomycin susceptible S26, non-resistant S4 and methicillin-resistant *in vivo*, MRSA, S2), *Enterococcus faecalis* (vancomycin susceptible, VSE, E1), *E. faecium* (vancomycin resistant, VRE, E2) and *S. pneumonia* (erythromycin susceptible P1 and multi drug resistant, MDRSP, P9) as well as Gram-negative bacteria such as *Pseudomonas aeruginosa*, *Escherichia coli* (efflux-positive Ec50 and -negative Ec49) and *Haemophilus influenzae* (efflux-positive H3 and -negative H4). In general, the activity trend for barbiturates is similar to that for tetramates [13,14,15,19,34]; firstly, none of the analogues was active against both *P. aeruginosa* and efflux-positive and -negative *E. coli*, (MIC ≥ 32 µg/mL) while the activity against the other strains depended on their ring substituents. Secondly, templates **40a**–**e**, 3-alkoxycarbonyls **42a**–**d**, and *O*-acyl derivative **41** did not exhibit antibacterial activity against any strains, while the activity of 3-acyls **2**–**28** (Figure 3), 3-carboxamides **29**–**32** and 3-enamines **33**–**37** (Figure 4) depended both upon the identity of the bacterial strains as well as their chemical substituents, with 3-acyls and 3-carboxamides tending to be more effective than 3-enamines. Third, *N*-disubstituted barbituric acids (especially 3-acyls) exhibited excellent activity whereas *N*-monosubstituted and *N*-unsubstituted analogues were inactive (see below in detail). These two results reveal that the functional group located on the C(3) position, as seen for tetramates (e.g., 3-acyl and 3-carboxamide), as well as the *N*-substitution in the barbituric acid templates, are critical factors for the observation of antibacterial activity. Lastly, of particular importance is that, depending on the substituents, the analogues exhibited excellent antibacterial selectivity against resistant and susceptible strains (S1, S26, S2, E1, E2, P1 and P9). By comparison, the activity of ciprofloxacin against MRSA S2 and VRE E2 and amoxicillin against MDRSP P9 dropped more than 50-fold compared to that of the non-resistant strain [15]. In conclusion, 3-acyls **7a**,**b** and 3-carboxamides **32** possessing adamantyl groups exhibited excellent antibacterial activity against Gram-positive strains and Gram-negative *H. influenzae* (MIC; up to 0.25 µg/mL).

Despite replacement of the tetramic acid with the more hydrophilic barbituric acid core, PPB affinity of barbiturates was only slightly reduced when compared with that of tetramates [13,14,15,19]; overall, MICs of barbiturates against *S. pneumonia* P9 in the presence of 2.5% horse blood were only slightly shifted from the values without blood (Table 1). For example, (±)-**5a**,**b**, **6a**,**b**, **7a**,**b**, **8a**,**b** and **18b**,**g** against *S. aureus* S26 in the presence of 10% human serum exhibited weak activity (MIC = 64 µg/mL) which were approximately 4-fold worse compared to those without serum. Moreover, it appears that the 3-acyl group might be better than the 3-carboxamide group for PPB binding in this family ((±)-**5b**; 8 to 64 µg/mL *versus* **31a**; 8 to >64 µg/mL, and **6a**; 16 to 64 µg/mL and **7a**; 1 to 64 µg/mL *versus* **32**; 1 to >64 µg/mL by 10% human serum).

**Table 1:** *In vitro* antibacterial activity (MIC, µg/mL) of barbituric acid analogues ^a−f^.

	S1	S26	S26S	S4	S2	E1	E2	P1	P9	P9B	H3	H4
(±)-**5a**	16	16	64	16	16	16	16	8	8	8	8	4
(±)-**5b**	4	8	64	4	8	4	8	4	4	4	32	8
(±)-**5c**	8	32	>64	32	16	16	16	16	16	16	64	32
**6a**	8	16	64	8	16	8	8	8	8	8	8	2
**6b**	4	8	64	4	8	4	8	4	4	4	16	4
**6c**	8	8	>64	4	4	4	4	4	4	4	64	16
**7a**	- ^c^	1	64	2	2	1	2	0.5	0.5	2	8	1
**7b**	0.5	2	64	2	1	0.5	1	2	2	4	16	8
**8a**	16	16	64	16	16	8	16	8	8	8	8	2
**8b**	4	4	64	2	4	0.5	2	1	1	1	8	2
**9**	32	32	>64	16	32	32	16	16	16	16	>64	16
**14**	>64	>64	>64	>64	>64	>64	>64	64	64	8	>64	64
**15a**	>64	- ^c^	- ^c^	>64	>64	>64	>64	32	32	>64	>64	64
**15b**	32	64	>64	32	32	32	32	32	16	16	>64	8
**16**	>64	>64	>64	>64	>64	64	64	32	32	32	>64	8
**17a**	64	>64	>64	64	64	64	64	32	32	32	16	8
**17c**	8	8	>64	8	8	8	4	4	4	4	16	2
**18a**	32	64	>64	64	64	32	32	32	32	32	32	4
**18b**	4	4	64	4	4	2	4	2	2	4	16	2
**18c**	64	64	>64	64	64	64	16	32	32	64	>64	32
**18e**	8	8	>64	8	8	4	4	4	4	8	16	2
**18f**	4	4	>64	4	4	4	4	2	2	2	16	2
**18g**	8	8	64	4	4	4	4	2	2	4	8	2
**19a**	1	2	>64	2	2	1	2	0.5	0.5	4	16	8
**19b**	16	16	>64	16	16	8	16	8	8	8	32	8
**20**	4	8	>64	8	8	4	2	2	2	4	16	2
**22**	64	64	>64	32	64	32	32	32	32	32	8	4
**23a**	64	64	>64	64	32	32	64	64	64	>64	32	16
**26c**	>64	>64	>64	>64	>64	>64	64	>64	>64	>64	32	64
**26d**	64	64	>64	64	32	64	64	64	>64	>64	32	16
**29a**	32	>64	>64	64	32	>64	64	64	64	64	32	16
**30**	32	64	>64	16	32	64	64	64	>64	>64	32	16
**31a**	8	8	>64	8	4	8	8	8	8	8	64	8
**31b**	2	4	>64	4	4	4	8	4	4	4	64	4
**32**	1	1	>64	0.5	1	0.25	0.5	0.25	0.25	0.5	2	0.25
**Line^d^**	2	2	2	2	2	2	2	1	0.5	0.5	16	4
**Cip^d^**	0.12	0.5	0.5	0.12	**16**	1	**32**	1	1	1	0.5	≤0.06
**Amo^d^**	- ^c^	- ^c^	- ^c^	- ^c^	- ^c^	- ^c^	- ^c^	>0.03	**8**	- ^c^	- ^c^	- ^c^

Notes: a; Abbreviation; **S1**; *S. aureus* 1, ATCC13709 *in vivo* (methicillin sensitive), **S26**; *S. aureus* 26, ATCC25923 (vancomycin susceptible), **S26S**; *S. aureus* 26 in presence of 10% human serum, **S4**; *S. aureus 4*, Oxford, **S2**; *S. aureus* 2, MRSA *in vivo* (methicillin resistant), **E1**; *E. faecalis* 1, ATCC29212 VanS (vancomycin susceptible), **E2**; *E. faecium* 1, VanA (vancomycin resistant), **P1**; *S. pneumonia* 1, ATCC49619 (erythromycin susceptible), **P9**; *S. pneumonia* 9, PenR (penicillin and erythromycin resistant), **P9B**; *S. pneumonia* 9 in presence of 2.5% horse blood, **H3**; *H. influenzae* 3, ATCC31517 MMSA, **H4**; *H. influenzae* 4, LS2 Efflux knock-out, **Line**; linezolid, **Cip**; ciprofloxacin, **Amo**; amoxicillin, b; All analogues are inactive against *E. coli* 1, ATCC25922 (non Pathogenic strain), *E. coli* 50, Ec49 No Efflux and *P. aeruginosa* 1, ATCC27853 (MIC > 32 μg/mL), c; Not determined, d; reported in our previous papers [13,14,15,19], e; 3-Acyls **2a**–**c**, **3**, *cis*-**11**–**13**, **17b**, **25a**, (±)-**25b** and **26a**,**b**, 3-carboxamides **29b**,**c**, 3-enamines **33a**,**b**, **35** and **36**, 3-alkoxycarbonyls **42a**-**d**, O-acyl **41** and barbituric acid templates **40a**-**e** were inactive against all strains (MIC > 32 μg/mL), f; 3-Acyls **4**, (±)-**10**, **18d**, **21**, **23b** and **24** and 3-enamines **34** and (±)-**37** were only mild active against H4 (8 ≤ MIC ≤ 32 μg/mL) while they were inactive against the other strains (MIC > 32 μg/mL).

### 2.4. Structure-Activity Relationships

Among the 3-acylbarbiturates, 3-acetyl derivatives **2a**–**c** did not exhibit antibacterial activity, whereas branched alkyls (±)-**5a**–**c** possessing a bulky lipophilic group exhibited good antibacterial activity. In addition, the adamantylacetyl substituent, possessing a similar steric effect to the branched alkyl group, retained activity ((±)-**5a** *versus* **6a**) while methyl substitutions at R_1_ and R_2_ in the adamantyl group did not affect the activity (**6a**–**c**). In contrast, replacements of the branched alkyl to smaller *tert*-butyl ((±)-**5b** *versus* **4**) but also of adamantyl to the smaller cyclohexyl (**7a** *versus* **8a**) decreased activity. Although the steric effect of **3**, **9**, (±)-**10** and *cis*-**11** is similar to active compounds (±)-**5a**, **7a**, **8b** and **6c**, respectively, their activities were abolished, probably by the polar heteroatom in the 3-acyl group. However, the longer bridge between the 3-acyl and cyclic functionality provided better activity (**6a** *versus* **7a**; **8a** *versus* **8b**). In addition, longer and polar *N*-substituents on the barbituric core gave similar activity (**7a** *versus* **7b**; (±)-**5a**,**b** *versus* (±)-**5c**) while the longer and lipophilic *N*-diethyl group provided improved activity compared with *N*-dimethyl groups ((±)-**5a** *versus* (±)-**5b**; **18g** *versus* **19a**). On the other hand, compounds (±)-**10**-**16**, all possessing bigger and more polar substituents which had been expected to give reduction of PPB affinity, had no or only slight activity.

From the previous finding that 3-acyltetramic acids possessing substituted phenyl groups with a C3-C4 chain length bridge exhibited good antibacterial activity [14], the SAR of **17**–**19** were studied in detail. It was found that lipophilicity is a crucial factor for cell permeability in the whole-cell antibacterial assay (see below for details). The more lipophilic analogues **17c** (compared with **17a**,**b**), **18b** (compared with **17a** and **18a**) and **19a** (compared with **18g** and **19b**) all exhibited better activity. Of particular interest is that di-substituted phenyl **18e** (R_1_ and R_4_) and **18f**,**g** (R_1_ and R_2_) exhibited better activity than di-substituted phenyl **18c** (R_2_ and R_3_) and **18d** (R_3_ and R_4_), even though they all possess similar steric effects and lipophilicity. This result indicates that the activity is sensitive to the nature of phenyl substitution (especially at R_1_ position). In addition, compounds **21**–**24** possessing polar atoms on the 3-acyl group and/or shorter bridge than **17**–**19** exhibited poor activity, with the exception of compound **20**.

As mentioned above, *N*-unsubstituted or -monosubstituted **25**–**28** dropped in activity, even though the 3-acyl functionality of (±)-**25b** and **26b**,**d** is identical to that of active barbiturates (±)-**5a**-**c** and **18g**, respectively. On the other hand, alkyl carboxamides **31** and **32** (possessing similar functionality with active 3-acyl barbiturates as well as 3-acyl and 3-carboxamide tetramic acids [13,14,15]) tended to exhibit better activity than aryl carboxamides **29** and **30** (possessing similar functionality with active 3-carboxamide tetramic acids), while 3-enamines **33**–**37** had no or only weak antibacterial activity, similarly to 3-enamine tetramic acids [19].

### 2.5. Physicochemical Property-Antibacterial Activity Relationships

**Figure 5:**
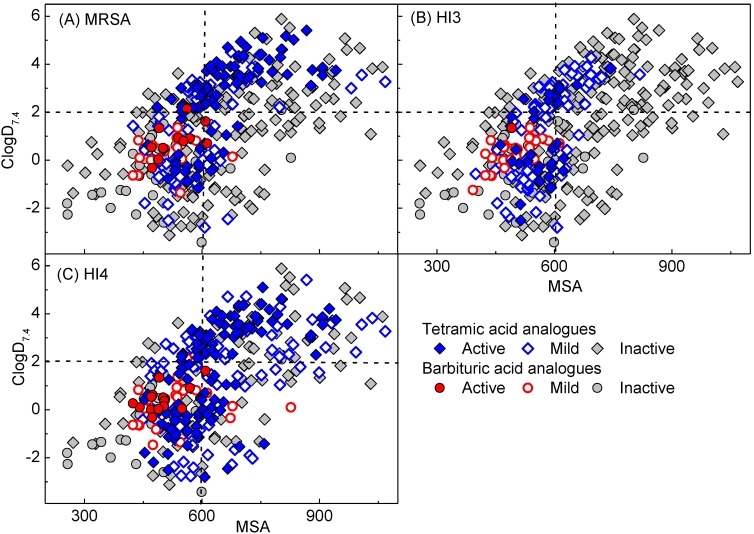
Plot of ClogD_7.4_ against MSA of barbituric acids **2**–**37** along with tetramic acids in our previous reports [13,14,15,19] against (**A**) MRSA; (**B**) *H. influenzae 3* and (**C**) efflux-negative *H. influenzae 4*. Active, mild and inactive mean that the values are MIC ≤ 4 µg/mL, 4 µg/mL < MIC ≤ 32 µg/mL and MIC > 32 µg/mL, respectively.

Examination of physicochemical property-activity relationships [35], especially in order to understand any trends for bacterial cell permeability, including transportation by efflux pump and PPB affinity, for antibiotic discovery was made. Figure 5 presents a plot of ClogD_7.4_ against molecular surface area (MSA) of 3-acyl (50 analogues), 3-carboxamide (7 analogues) and 3-enamine (6 analogues) barbituric acids **2**–**37** along with the corresponding tetramic acids (326 analogues) from our previous reports [13,14,15,19], classified into active (MIC ≤ 4 µg/mL), mild (4 < MIC ≤ 32 µg/mL) and inactive (MIC > 32 µg/mL) analogues. In addition, Supplementary Figures 3–5 in the Supporting Information presents the plots of ClogP, polar surface area (PSA) and relative-PSA (rel-PSA = PSA/MSA), respectively (see also Supplementary Table 1 in Supporting Information for physicochemical properties in detail). As shown in Figure 5A and Supplementary Figures 3A–5A, analogues with a wide range of physicochemical properties permeate Gram-positive bacteria whereas the cell permeability of Gram-negative bacteria is limited to a much narrower range of physicochemical properties (with an especially higher threshhold for lipophilicity). This is clearly indicated by the limited activity against efflux-positive *H. influenzae* H3 shown in Figure 5B and the inactivity against *P. aeruginosa* and *Escherichia coli*. Since analogues with a wider range of physicochemical properties exhibit better antibacterial activity against efflux-negative *H. influenzae* H3 than the efflux-positive strain (Figure 5B,C), and the fact that tetramic acids exhibited antibacterial activity against TolC negative *E. coli* and *Klebsiella pneumonia* in our previous study [13], the main obstacle to Gram-negative bacteria cell permeability appears to transportation by efflux-pumps.

In this analysis, barbiturates active against MRSA (red-filled circles) and efflux-negative H4 are positioned in the same range of ClogD_7.4_, ClogP, PSA and MSA for active tetramates (blue-filled circles) and tend to be less lipophilic, have higher PSA and smaller MSA character than active 3-acyltetramic acids [14,19] (Figure 5A,C and Supplementary Figures 3A,C and 4A,C); however, active barbiturates possess slightly higher rel-PSA than active tetramates (Supplementary Figure 5A,C). Of particular interest is that the active barbiturates possess similar physicochemical properties to clinical antibiotics for oral or injectable use (−3.0 < ClogD_7.4_ < 2.0; 0 < ClogP < 3.0; 60 < PSA < 120 Å^2^; 10 < rel-PSA < 30% and 270 < MSA < 650 Å^3^, see below) as well as acceptable molecular weight (<400 Da), rotatable bonds (usually less than 6) and the number of proton-donor (1–2) and -acceptor (4–6) for oral availability in the rule of five (Supplementary Table 1) [36].

In our previous analysis with tetramates [13,14,19] presented as blue-filled circles in Figure 5B and C and Supplementary Figure 3B,C, tetramates possessing less lipophilic (ClogD_7.4_ < 3.0 and ClogP < 4.0) and smaller (MSA < 620 Å^3^) characteristics tended to be less easily transported by efflux pumps in *H. influenzae*. Although the active barbiturates are in this zone of lipophilicity and MSA, they were slightly more easily transported than tetramates. This may be due to the fact that barbiturates possess higher PSA (78 < PSA < 100 Å^2^) than tetramates (65< PSA < 90 Å^2^), are in the same range of MSA (420 < MSA < 650 Å^3^) and this results in higher rel-PSA (15 < rel-PSA < 20% *versus* 10 < rel-PSA < 15%). This phenomena agrees with the observation that compounds possessing higher topological PSA are more easily transported by multidrug resistance-associated protein 1 (MRP1/ABBC1) [37].

### 2.6. Physicochemical Property-Plasma Protein Binding Affinity Relationships

**Figure 6:**
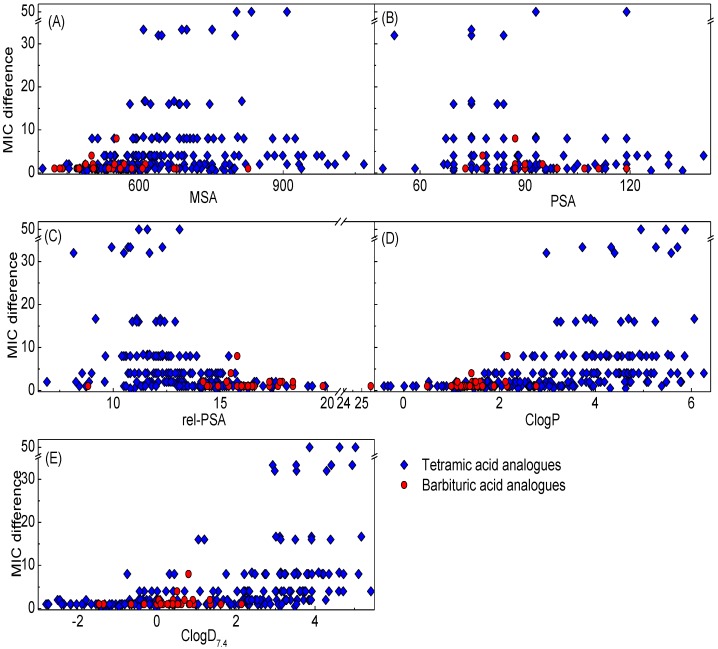
Plot of MIC difference against (**A**) MSA; (**B**) PSA; (**C**) rel-PSA; (**D**) ClogP (**E**) ClogD_7.4_ of barbituric acids along with tetramic acids in our previous reports [13,14,15,17]. The MIC difference is defined as MIC with 2.5% blood/ MIC without blood against *S. pneumonia* 9.

In order to investigate physicochemical property-PPB affinity relationships, plots of MIC difference against MSA, PSA, rel-PSA, ClogP and ClogD_7.4_ of barbiturates (32 analogues) used in this study along with tetramates (208 analogues) from our previous studies [13,14,15,17] were made (Figure 6). In this analysis, the MIC difference is defined as the value from MIC against *S. pneumonia* P9 in the presence of 2.5% blood divided by MIC without blood (inactive analogues against any one of these panels were not considered). Since PPB affinity is affected by multiple interactions in many proteins such as human serum albumin, lipoprotein, glycoprotein and globulins in blood, there is no linear correlation between the MIC difference and the physicochemical properties in Figure 6. However, this analysis, which uses a larger number of analogues than in our previous analysis [13,14], shows clearer trends (especially against MSA and PSA). As with the ClogP-PPB affinity relationship in the literature [38,39], PPB affinity is more closely related to lipophilicity as represented by rel-PSA, ClogP and ClogD_7.4_ (Figure 6C–E) than MSA and PSA (Figure 6A,B). It appears that less lipophilic analogues (rel-PSA > 16%, ClogP < 1.5 and ClogD_7.4_ < −0.69) exhibit higher probability of having lower PPB affinity (MIC difference ≤ 2) while those analogues with high PPB affinity (MIC difference > 30) are positioned in the area of highly lipophilic regions (rel-PSA < 13%, ClogP > 2.9 and ClogD_7.4_ > 2.9, Figure 6C–E). Although the trends of PPB affinity with steric effect (MSA) and PSA are weaker than lipophilicity, the analogues in the range of MSA between 600 and 900 Å^3^ and PSA being less than 100 Å^2^ exhibit a higher probability of possessing high PPB affinity (Figure 6A,B). However, the barbiturate library (red-filled circles) tended to exhibit lower PPB affinity compared to the tetramate library because of their less lipophilic (rel-PSA > 14%, ClogP < 2.0 and ClogD_7.4_ < 1.0) and smaller MSA (MSA < 650 Å^3^) character on average.

In fact, potency enhancements of 3-acyltetramic acids [13,14] arising from improvement of lipophilicity and molecular size (ClogD_7.4_ >2.0 and MSA >600 Å^3^) are unlikely to provide both lower PPB affinity as well as efflux pump transport. In contrast, we believe that active barbiturates and 3-carboxamide tetramic acids [13,15] exhibit amenable PPB affinity for *in vivo* activity because of their similar physicochemical properties with clinical antibiotics used for oral or injectable administration as well as being an anionic microspecies under weakly basic conditions (see below). Indeed, the ability to control PPB affinity by adjustment of physicochemical properties proved to be limited; therefore, although appropriate physicochemical properties might be necessary for overcoming PPB affinity, there appear to be other factors involved. One possible hypothesis is that the main core (tetramic and barbituric acids) with its inherent acidic character might be responsible for binding to serum albumin, the major protein in blood, at the sites for aromatic carboxylic acids such as salicylates and ibuprofen. In order to understand whether this is the case, computational and NMR study of serum albumin affinity with our analogues is under investigation.

### 2.7. Physicochemical Property-Activity Relationships of Small Molecule Antibacterial Agents

**Figure 7:**
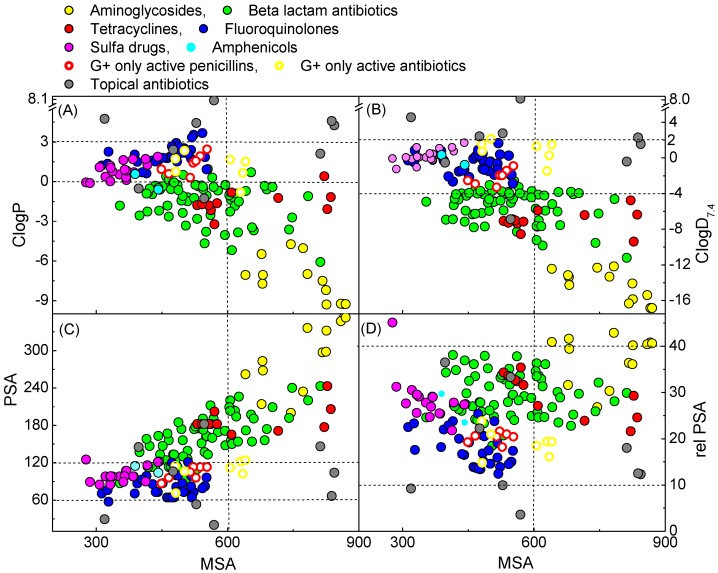
Plot of (**A**) ClogD_7.4_, (**B**) ClogP, (**C**) PSA and (**D**) rel-PSA against MSA of small molecule antibacterial agents.

Although physicochemical properties of antibacterial agents have been discussed in detail in the previous literature [1,40], an examination of the desirable properties for small molecule antibacterial agents, especially to understand cell permeability and PPB affinity, was made using cheminformatic analysis. In order to achieve a more reliable comparison with our library (Mw < 650 Da), antibiotic families with a large molecular weight (Mw > 600 Da), such as glycopeptides which act at the peptidoglycan layer and do not therefore require the penetration of a lipid membrane, and macrolides, have been excluded. In this study, 8 bins for each antibiotic family along with a separate bin for topical antibiotic agents but without consideration of their family, were created. The plots of lipophilicity descriptors (ClogD_7.4_, and ClogP) and polar surface descriptors (PSA and rel-PSA) against steric effect descriptor (MSA) were made (Figure 7, see also Supplementary Table 2–10 in Supporting Information for the physicochemical properties in detail). In this analysis, most antibiotics, with the exception of topical agents, tended to have a higher limit for lipophilicity (ClogD_7.4_ < 2.0 and ClogP < 3.0) and a lower limit for polar surface area (PSA > 60 Å^2^ and rel-PSA > 13%). Furthermore, it is noteworthy that the bigger antibiotics (MSA > 650 Å^3^) tend to have a stricter limit for lipophilicity (ClogD_7.4_ < −4.0 and ClogP < 0) and polar surface area (PSA > 120 Å^2^ and rel-PSA > 20%). That these margins might result from PPB affinity is supported by the fact that topical agents, for which PPB affinity is less important, are free from these boundaries, although of course other factors such as intrinsic antibacterial activity and ADMET are clearly important (for example, antibiotics with higher antibacterial activity compensate for higher PPB affinity for oral or injectable use). However, the lipophilicity (ClogD_7.4_ and ClogP) and the PSA are in inverse proportion to the MSA while the rel-PSA is not affected by the MSA, generally displaying values between 12%–38%. The analogues as shown in Figure 5 and Supplementary Figures 3–5 are opposite to this tendency. Since bigger tetramic acids (MSA > 600 Å^3^) usually possess more lipophilic character, it would be worth investigating antibacterial activity and PPB affinity of libraries with bigger and less lipophilic character (MSA > 700 Å^3^, ClogD_7.4_ < −4.0) in the future.

From a consideration of their physicochemical properties, on the other hand, the 8 bins for oral or injectable antibiotics can be classified into 3 sub-types. The first one is aminoglycosides populating the most hydrophilic (lowest ClogP and ClogD_7.4_ and highest PSA and rel-PSA) and the biggest (MSA > 600 Å^3^) regions. Due to this hydrophilicity (ClogD_7.4_ < −12), their oral administration has been generally limited as a result of problems with absorption. The second class includes Gram-negative active β-lactams and tetracyclines, and this class of antibiotics possesses lipophilicity and MSA between aminoglycosides and the third class of antibiotics. The third class includes antibiotics of natural origin such as Gram-positive only active penicillins and amphenicols as well as synthetic origin antibiotics such as fluoroquinolones, sulfa drugs and oxazolidinones, possessing higher lipophilic character and smaller MSA than both of the other classes. Of particular interest is that the third class displays a narrow zone of physicochemical properties (−3.0 < ClogD_7.4_ < 2.0; 0 < ClogP < 3.0; 60 < PSA < 120 Å^2^, 270 < MSA < 650 Å^3^) with the exception of rel-PSA, while the two other classes populate a wider range of the physicochemical space, with the preference for lower lipophilicity and bigger MSA than the third class. The physicochemical space populated by the third class is amenable to general drug design, and appears to be the most suitable for antibiotic development. As shown in Figure 5 and Supplementary Figures 3–5, our active barbiturates in this study and 3-carboxamide tetramic acids from the previous study [13,15] also satisfy these properties, in contrast to active 3-acyltetramic acids which generally possess higher ClogP and ClogD_7.4_ values [13,14]. From a consideration of MSA of the third class of antibacterial agents (MSA < 650) as well as from the fact that smaller molecules (MSA < 620 Å^3^) tend to be less easily transported by the efflux pump in *H. influenzae* as described above (Figure 5B,C), and that analogues with 600 < MSA < 900 Å^3^ exhibit a higher probability of higher PPB affinity (Figure 6A), both lipophilicity and molecular size might be crucial factors for antibiotic discovery in which smaller analogues (MSA < 600 Å^3^) are likely to have a benefit for both PPB affinity and cell permeability.

### 2.8. Ionic State of Small Molecule Antibacterial Agents

Since for bacterial cell permeability (including the transportation by efflux pump), ionic state (related to pKa) might be expected to be a major factor, the major microspecies at pH 7.4 were calculated (data not shown, see Supplementary Figures 6–14 for the structures of antibacterial agents). Of particular interest is that Gram-negative active agents favoured anionic(s) and zwitterionic(s) microspecies, while aminoglycosides exist as cationic forms, generally as a result of the presence of the amine functionality. Fluoroquinolones, tetracyclines and Gram-negative active β-lactams exist as various forms of zwitterionic and anionic microspecies. This might result from the fact that their key skeletons include an acidic carboxylic acid (fluoroquinolones and β-lactams) or two acidic enols and a basic amine group (tetracyclines), which make them easily able to form anionic or zwitterionic species under weakly basic conditions (pH = 7.4). In addition, sulfa drugs mainly exist as an anionic form on the nitrogen in the sulfone amide group, in some cases with a minor amount of the uncharged form. In this family, sulfaguanidine (used in the treatment of gastrointestinal infections) exceptionally exists as a neutral microspecies under weakly basic conditions but is cationic in acidic conditions (pH = 1.0)_._ In addition, amphenicols also exist as a mixture of a neutral and an anionic (on the nitrogen of amide functionality) microspecies. On the other hand, Gram-positive only active antibiotics generally exist as an anionic (rather than a zwitterionic, Gram-positive only β-lactams), neutral (oxazolidinones) or a mixture of a neutral and cationic (lincosamides and 2,4-diaminopyrimidines) microspecies, and tend to be less charged state than Gram-negative active agents. In general, Gram-negative antibacterial agents tend to be more charged than the Gram-positive only agents to penetrate outer membrane via porins as well as prevent efflux.

## 3. Experimental Section

### 3.1. General

All starting materials and 3-acyl *2a* were commercially available. Melting points were checked by STUART SCIENTIFIC SMP1. The ^1^H-, ^13^C- and HMBC-NMR spectra were obtained using a Bruker AVB500 (500 MHz for ^1^H-NMR and 126 MHz for ^13^C-NMR) or DPX400 (400 MHz ^1^H-NMR and 101 MHz for ^13^C-NMR). Mass spectra (MS) and high resolution MS were obtained with Micro Mass LCT and GCT spectrometers under the conditions of electrospray ionization (ESI) and chemical ionization (CI) respectively, and values were reported as a ratio of mass to charge in Daltons. The energies at ground state were computed with Density Functional B3LYP (6-31G*) in Spartan 02 in each enol form and the physicochemical properties were calculated with MarvinSketch version 5.3.8. in which VG method for ClogP and ClogD_7.4_, van der Waals method for MSA were selected and sulfur atoms were excluded in the calculation of PSA. *In vitro* antibacterial activity was performed using standard methodology as described in our previous paper [13].

### 3.2. Synthesis of Barbituric Acid Templates **40a**–**c**

#### 3.2.1. Synthesis of Compounds **40c** and **2c**

##### Synthesis of Urea **39c**

To the solution of 2-methoxy ethylamine **38** (3.2 g, 42.2 mmol) in dichloromethane (50 mL) was slowly added ethyl isocyanate (3.0 g, 42.2 mmol) at 0 °C under nitrogen atmosphere and the mixture was stirred for 30 min. Concentration in *vacuo* followed by precipitation in ethyl acetate and petrol solution gave urea **30c** (5.9 g, 40.2 mmol, 95% yield) as a solid (M.P.; 40.0 °C).


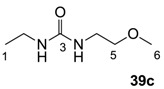


^1^H-NMR (400 MHz, CDCl_3_); 5.56 (brs, 1H, NH), 5.46 (brs, 1H, NH), 3.40 (t, 2H, *J* = 5.2 Hz, C5), 3.32–3.28 (m, 5H, C4 and C6), 3.17–1.10 (m, 2H, C2), 1.06 (t, 3H, *J* = 7.2 Hz, C1). ^13^C-NMR (100 MHz, CDCl_3_); 159.0 (C3), 72.2 (C5), 58.5 (C6), 39.9 (C4), 34.8 (C2), 15.3 (C1). MS (ES^+^); 169.08 (M+Na); HRMS (M+Na); calcd for C_6_H_14_N_2_Na_1_O_2_; 169.0947; found; 169.0950.

##### Synthesis of Barbituric Acid **40c** and 3-acetylbarbituric Acid **2c**

To the solution of urea **39c** (5.9 g, 40.15 mmol) in acetic acid (80 mL) was added malonic acid (3.9 g, 40.15 mmol) at room temperature. The mixture was slowly added acetic anhydride (60 mL) at 60 °C for 30 min and the mixture was stirred at 90 °C for 5 h. Concentration in vacuo followed by flash column chromatography gave barbituric acid **40c** (6.1 g, 28.5 mmol, 71%) as oil and 3-acetylbarbituric acid **2c** (1.0 g, 3.90 mmol, 10%) as oil.

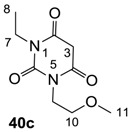

Compound **40c**; ^1^H-NMR (400 MHz, CDCl_3_); 4.01 (t, 2H, *J* = 5.6 Hz, C9), 3.84 (q, 2H, *J* = 6.8 Hz, C7), 3.59 (s, 2H, C3), 4.01 (t, 2H, *J* = 5.6 Hz, C10), 3.24 (s, 3H, C11), 1.11 (t, 3H, *J* = 6.8 Hz, C8). ^13^C NMR (100 MHz, CDCl_3_); 164.6 (C=O), 164.3 (C=O), 151.1 (C6), 68.9 (C10), 58.4 (C11), 40.3 (C9), 39.4 (C3), 37.0 (C7), 12.9 (C8). MS (ES^−^); 213.08 (M−H); MS (ES^+^); 237.09 (M+Na); HRMS (M+Na); calcd for C_9_H_14_N_2_Na_1_O_4_; 237.0846; found; 237.0848.

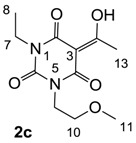

Compound **2c**; ^1^H-NMR (400 MHz, CDCl_3_); 4.17–4.12 (m, 2H, C9), 4.01–3.93 (m, 2H, C7), 3.62–3.56 (m, 2H, C10), 3.33 and 3.32 (2 of s, 3H, C11), 2.69 and 2.68 (2 of s, 3H, C13), 1.24–1.17 (m, 3H, C8). ^13^C-NMR (100 MHz, CDCl_3_); 196.3 and 196.1 (C12), 169.4 and 169.2 (C2), 160.9 and 160.7 (C4), 149.8 and 149.7 (C6), 96.0 and 95.9 (C3), 69.3 and 69.2 (C10), 58.7 and 58.6 (C11), 40.1 and 40.0 (C9), 36.7 and 36.6 (C7), 24.7 and 24.6 (C13), 13.1 (C8). MS (ES^−^); 255.11 (M−H); MS (ES^+^); 279.10 (M+Na); HRMS (M+Na); calcd for C_11_H_16_N_2_Na_1_O_5_; 279.0951; found; 279.0946.

#### 3.2.2. Synthesis of Barbituric Acid **40b** and 3-acetylbarbituric Acid **2b**

Barbituric acid **40b** (3.9 g, 21.2 mmol, 82%) as oil and 3-acetylbarbituric acid **2b** (660 mg, 2.93 mmol, 11%) as a solid (M.P.; 68 °C) were obtained from 1,3-diethyl urea **39b** (3.0 g, 25.8 mmol) and malonic acid (2.7 g, 25.8 mmol) as the same method with synthesis of compounds **40c** and **2c**.

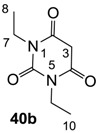

Compound **40b**; ^1^H-NMR (400 MHz, CDCl_3_); 3.87 (q, 4H, *J* = 7.2 Hz, C7 and C9), 3.60 (s, 2H, C3), 1.15 (t, 6H, *J* = 7.2 Hz, C8 and C10). ^13^C-NMR (100 MHz, CDCl_3_); 164.4 (C2 and C4), 150.9 (C6), 39.5 (C3), 37.0 (C7 and C9), 13.0 (C8 and C10). MS (ES^−^); 183.06 (M−H); HRMS (M−H); calcd for C_8_H_11_N_2_O_3_; 183.0775; found; 183.0771.

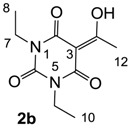

Compound **2b**; ^1^H-NMR (400 MHz, CDCl_3_); 4.00–3.92 (m, 4H, C7 and C9), 2.67 (s, 3H, C12), 1.23–1.16 (m, 6H, C8 and C10). ^13^C-NMR (100 MHz, CDCl_3_); 196.1 (C11), 169.2 (C2), 160.7 (C4), 149.5 (C6), 95.9 (C3), 36.5 (N-CH_2_), 36.4 (N-CH_2_), 24.7 (C12), 13.1 (C8 and C10). MS (ES^−^); 225.08 (M−H); HRMS (M−H); calcd for C_10_H_13_N_2_O_4_; 225.0881; found; 225.0882.

#### 3.2.3. Synthesis of Barbituric Acid **40a**

To the solution of N-allylurea **39a** (2.0 g, 20.0 mmol) in acetic acid (60 mL) was added malonic acid (2.1 g, 20.0 mmol) at room temperature. The mixture was slowly added acetic anhydride (40 mL) at 60 °C for 30 min and the mixture was stirred at 90 °C for 5 h. Concentration in *vacuo* followed by precipitation in ethyl acetate gave barbituric acid **40a** (960 mg, 5.71 mmol, 30%) as a solid (M.P.; 158 °C).


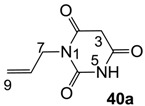


^1^H-NMR (400 MHz, DMSO, keto form); 11.35 (brs, 1H, NH), 5.82–5.72 (m, 1H, C8), 5.17 (dd, 1H, *J_1_* = 17.6 Hz, *J_2_* = 1.6 Hz, C9), 5.08 (dd, 1H, *J_1_* = 10.4 Hz, *J_2_* = 1.6 Hz, C9), 4.27 (d, 2H, *J* = 4.8 Hz, C7), 3.64 (s, 2H, C3). ^13^C-NMR (100 MHz, DMSO, keto form); 166.5 (C2 and C4), 151.4 (C6), 132.5 (C8), 116.2 (C9), 41.9 (C7), 39.9 (C3). ^1^H-NMR (400 MHz, CD_3_OD, enol form); 5.89–5.79 (m, 1H, C8), 5.23 (dd, 1H, *J_1_* = 17.2 Hz, *J_2_* = 1.2 Hz, C9), 5.14 (dd, 1H, *J_1_* = 10.0 Hz, *J_2_* = 1.2 Hz, C9), 4.84 (brs, 2H, NH, OH and C3), 4.40 (d, 2H, *J* = 5.6 Hz, C7). ^13^C-NMR (100 MHz, CD_3_OD, enol form, due to deuterium exchange, C3 signal is not appeared); 168.2 (C2 and C4), 152.9 (C6), 133.4 (C8), 118.0 (C9), 43.9 (C7). MS (ES^−^); 167.04 (M−H), HRMS (M−H); calcd for C_7_H_7_N_2_O_3_; 167.0462; found; 167.0454.

### 3.3. Synthesis of 3-alkoxycarbonyl Barbituric Acid Templates 41a–d

General procedure; To the solution of barbituric acid (1 eq) and DMAP (2.2 eq) in dichloromethane was slowly added butyl chloroformate (1.2 eq) at 0 °C under nitrogen atmosphere, and the mixture was stirred overnight at room temperature under nitrogen atmosphere. Then the mixture was washed with 2 M HCl. The organic layer was dried over MgSO_4_ and evaporated *in vacuo* to give 3-alkoxycarbonyl barbituric acid templates **41a**–**d** contained about 10%–20% impurity. The impure 3-alkoxycarbonyl barbituric acid template was used for next step without further purification.

#### 3.3.1. Synthesis of Compound **41a**


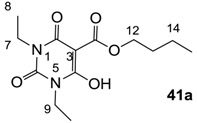


Yield; 85%; M.P. 33 °C; ^1^H-NMR (400 MHz, CDCl_3_); 4.33 (t, 2H, *J* = 6.8 Hz, C12), 4.03–3.92 (m, 4H, C7 and C9), 1.78–1.71 (m, 2H, C13), 1.48–1.39 (m, 2H, C14), 1.25 (t, 3H, *J* = 6.8 Hz, CH_3_), 1.17 (t, 3H, *J* = 6.8 Hz, CH_3_), 0.92 (t, 3H, *J* = 7.2 Hz, C15). ^13^C-NMR (100 MHz, CDCl_3_); 173.5 (C11), 168.7 (C2), 158.2 (C4), 148.9 (C6), 82.9 (C3), 66.5 (C12), 37.7 (N-CH_2_), 36.4 (N-CH_2_), 30.4 (C13), 18.9 (C14), 13.6 (C15), 13.3 (CH_3_), 12.9 (CH_3_). MS (ES^−^); 283.14 (M−H); MS (ES^+^); 307.13 (M+Na); HRMS (M+Na); calcd for C_13_H_20_N_2_Na_1_O_5_; 307.1264; found; 307.1256.

#### 3.3.2. Synthesis of Compound **41b**


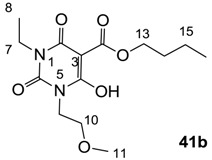


Yield; 65% (oil); ^1^H-NMR (400 MHz, CDCl_3_); 4.34 (t, 2H, *J* = 6.8 Hz, C13), 4.17–3.90 (m, 4H, C7 and C9), 3.61–3.54 (m, 2H, C10), 3.30 (s, 3H, C11), 1.78–1.70 (m, 2H, C14), 1.45–1.40 (m, 2H, C15), 1.25–1.14 (m, 3H, C8), 0.92 (t, 3H, *J* = 7.2 Hz, C16). ^13^C-NMR (100 MHz, CDCl_3_); 173.5 and 173.4 (C12), 169.0 and 168.8 (C2), 158.3 and 158.0 (C4), 149.3 and 149.1 (C6), 82.8 (C3), 69.1 (C10), 66.5 (C13), 58.5 (C11), 41.2 and 40.0 (C9), 37.8 and 36.5 (C7), 30.4 (C14), 18.9 (C15), 13.5 (C16), 13.2 (C8). MS (ES^−^); 313.15 (M−H); MS (ES^+^); 315.16 (M+H), 337.14 (M+Na); HRMS (M+Na); calcd for C_14_H_22_N_2_Na_1_O_6_; 337.1370; found; 337.1369.

#### 3.3.3. Synthesis of Compound **41c**


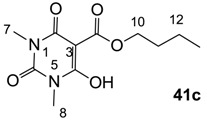


Yield; 90%; M.P.; 59 °C; ^1^H-NMR (500 MHz, CDCl_3_); 4.29 (t, 2H, *J* = 5.5 Hz, C10), 3.28 (brs, 6H, C7 and C8), 1.73–1.67 (m, 2H, C11), 1.44–1.37 (m, 2H, C12), 0.88 (t, 3H, *J* = 7.5 Hz, C13). ^13^C-NMR (125 MHz, CDCl_3_); 173.2 (C9), 168.7 (C2), 158.3 (C4), 149.6 (C6), 82.6 (C3), 66.4 (C10), 30.2 (C11), 28.4 (N-CH_3_), 27.8 (N-CH_3_), 18.8 (C12), 13.4 (C13). MS (ES^−^); 255.09 (M−H); HRMS (M−H); calcd for C_11_H_15_N_2_O_5_; 255.0986; found; 255.0987.

#### 3.3.4. Synthesis of Compound **41d**


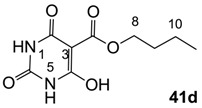


Yield; 56 %; M.P.; 259 °C; ^1^H-NMR (400 MHz, DMSO); 11.58 (brs, 2H, NH), 4.23 (t, 2H, *J* = 6.4 Hz, C8), 1.65–1.58 (m, 2H, C9), 1.40–1.35 (m, 2H, C10), 0.89 (t, 3H, *J* = 7.2 Hz, C11). ^13^C-NMR (125 MHz, DMSO); 171.9 (C7), 167.9 (C2), 165.0 (C4), 148.8 (C6), 82.2 (C3), 65.2 (C8), 30.2 (C9), 18.6 (C10), 13.6 (C11). MS (ES^−^); 227.07 (M−H); MS (ES^+^); 251.07 (M+Na); HRMS (M+Na); calcd for C_9_H_12_N_2_Na_1_O_5_; 251.0638; found; 251.0640.

### 3.4. Direct acylation of Barbituric Acid Templates

General procedure: To a solution of carboxilic acid (1.0 eq) in dichloromethane were added DCC (1.1 eq), barbituric acid (1.0 eq) and DMAP (1.2 eq) at room temperature. The mixture was stirred overnight at room temperature. The crude reaction mixture was filtered with dichloromethane. Concentration in vacuo followed by flash column chromatography gave metal-chelated barbituric acid. The crude product was dissolved in dichloromethane and washed with 1M HCl. The organic layer was dried with MgSO_4_ and concentrated in vacuo to give 3-acylbarbituric acid.

#### 3.4.1. Synthesis of Compound **3**


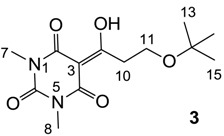


Yield; 51%; M.P.; 92 °C; ^1^H-NMR (400 MHz, CDCl_3_); 3.73 (t, 2H, *J* = 6.4 Hz, C11), 3.36 (t, 2H, *J* = 6.4 Hz, C10), 3.35 (s, 3H, C7), 3.31 (s, 3H, C8), 1.16 (s, 9H, C13–C15). ^13^C-NMR (100 MHz, CDCl_3_); 197.5 (C9), 169.7 (C2), 160.8 (C4), 150.3 (C6), 95.7 (C3), 73.2 (C12), 57.5 (C11), 38.3 (C10), 28.0 (N-CH_3_), 27.8 (N-CH_3_), 27.4 (C13–C15). MS (ES^−^); 283.14 (M−H), MS (ES^+^); 307.14 (M+Na), HRMS (M+Na); calcd for C_13_H_20_N_2_Na_1_O_5_; 307.1264; found; 307.1269.

#### 3.4.2. Synthesis of Compound **4**


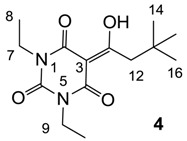


Yield; 57% (oil); ^1^H-NMR (400 MHz, CDCl_3_); 3.98–3.89 (m, 4H, C7 and C9), 3.14 (s, 2H, C12), 1.22–1.13 (m, 6H, C8 and C10), 1.02 (s, 9H, C14–C16). ^13^C-NMR (100 MHz, CDCl_3_); 198.5 (C11), 169.5 (C2), 160.6 (C4), 149.3 (C6), 96.8 (C3), 46.8 (C12), 36.5 (C7 and C9), 33.4 (C13), 29.9 (C14–C16), 13.1 (C8 and C10). MS (ES^−^); 281.15 (M−H); HRMS (M−H); calcd for C_14_H_21_N_2_O_4_; 281.1507; found; 281.1505.

#### 3.4.3. Synthesis of Compound (±)-**5b**


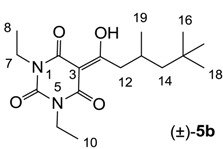


Yield; 71% (oil); ^1^H-NMR (400 MHz, CDCl_3_); 4.03–3.94 (m, 4H, C7 and C9), 3.08 (d, 2H, *J* = 7.2 Hz, C12), 2.22–2.12 (m, 1H, C13), 1.35 (dd, 1H, *J_1_* = 14.0 Hz, *J_2_* = 3.2 Hz, C14), 1.26–1.15 (m, 7H, C8, C10 and C14), 1.01 (d, 3H, *J* = 6.8 Hz, C19), 0.90 (s, 9H, C16-C18). ^13^C-NMR (100 MHz, CDCl_3_); 199.3 (C11), 169.6 (C2), 160.5 (C4), 149.7 (C6), 96.0 (C3), 50.7 (C14), 45.5 (C12), 36.6 (NCH_2_), 36.5 (NCH_2_), 31.1 (C15), 29.9 (C16–C18), 27.7 (C13), 22.7 (C19), 13.2 (C8 and C10). MS (ES^−^); 323.20 (M−H); HRMS (M−H); calcd for C_17_H_27_N_2_O_4_; 323.1976; found; 323.1980.

#### 3.4.4. Synthesis of Compound (±)-**5c**


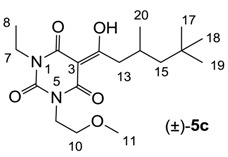


Yield; 42% (oil); ^1^H-NMR (400 MHz, CDCl_3_); 4.17–4.12 (m, 2H, C9), 4.00–3.92 (m, 2H, C7), 3.62–3.55 (m, 2H, C10), 3.33 and 3.32 (2 of s, 3H, C11), 3.06 (d, 2H, *J* = 7.2 Hz, C13), 2.19–2.12 (m, 1H, C14), 1.35–1.30 (m, 1H, C15), 1.24–1.12 (m, 4H, C8 and C15), 0.99 and 0.98 (2 of d, 3H, *J* = 6.8 Hz, C20), 0.87 (s, 9H, C17–C19). ^13^C-NMR (100 MHz, CDCl_3_); 199.3 and 199.2 (C12), 169.7 and 169.5 (C2), 160.6 and 160.3 (C4), 149.7 and 149.6 (C6), 95.9 and 95.8 (C3), 69.3 and 69.2 (C10), 58.7 and 58.6 (C11), 50.6 (C15), 45.4 and 45.3 (C13), 40.1 and 40.0 (C9), 36.7 and 36.6 (C7), 31.0 (C16), 29.8 (C17–C19), 27.7 (C14), 22.7 (C20), 13.1 (C8). MS (ES^−^); 353.22 (M−H); MS (ES^+^); 355.23 (M+H), 377.20 (M+Na); HRMS (M+Na); calcd for C_18_H_30_N_2_Na_1_O_5_; 377.2047; found; 377.2040.

#### 3.4.5. Synthesis of Compound **6a**


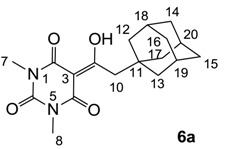


Yield; 90%; M.P.; 133 °C; ^1^H-NMR (500 MHz, CDCl_3_); 3.35 (s, 3H, C7), 3.31 (s, 3H, C8), 3.06 (s, 2H, C10), 1.99 (brs, 3H, C18–C20), 1.65–1.34 (m, 12H, C12–C17). ^13^C-NMR (125 MHz, CDCl_3_); 197.8 (C9), 169.7 (C2), 161.1 (C4), 150.2 (C6), 97.1 (C3), 47.9 (C10), 42.6 (CH_2_), 36.6 (CH_2_), 36.3 (C11), 28.8 (N-CH_3_), 28.1 (CH), 27.8 (CH). MS (ES^−^); 331.18 (M−H), HRMS (M−H); calcd for C_18_H_23_N_2_O_4_; 331.1663; found; 331.1657.

#### 3.4.6. Synthesis of Compound **6b**


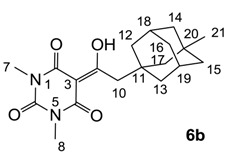


Yield; 54% (oil); ^1^H-NMR (400 MHz, CDCl_3_); 3.36 (s, 3H, C7), 3.31 (s, 3H, C8), 3.08 (s, 2H, C10), 1.99 (brs, 2H, C18 and C19), 1.65–1.34 (m, 12H, C12-C17), 0.78 (s, 3H, C21). ^13^C-NMR (100 MHz, CDCl_3_); 197.8 (C9), 169.7 (C2), 161.1 (C4), 150.2 (C6), 97.1 (C3), 49.6 (CH_2_), 47.6 (C10), 43.6 (CH_2_), 41.8 (CH_2_), 37.0 (C11), 35.8 (CH_2_), 30.9 (C21), 30.8 (C20), 29.3 (C18 and C19), 28.1 (N-CH_3_), 27.8 (N-CH_3_). MS (ES^−^); 345.20 (M−H), HRMS (M−H); calcd for C_19_H_25_N_2_O_4_; 345.1820; found; 345.1811.

#### 3.4.7. Synthesis of Compound **6c**


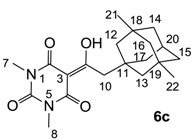


Yield; 39%; M.P.; 77 °C; ^1^H-NMR (400 MHz, CDCl_3_); 3.37 (s, 3H, C7), 3.32 (s, 3H, C8), 3.10 (s, 2H, C10), 2.05–2.02 (m, 1H, C20), 1.52–1.07 (m, 12H, C12–C17), 0.79 (s, 6H, C21 and C22). ^13^C-NMR (100 MHz, CDCl_3_); 197.8 (C9), 169.7 (C2), 161.1 (C4), 150.2 (C6), 97.1 (C3), 50.8 (CH_2_), 48.9 (CH_2_), 47.3 (C10), 42.9 (CH_2_), 41.0 (CH_2_), 37.7 (C18 and C19), 31.5 (C11), 30.5 (C21 and C22), 29.8 (C20), 28.2 (N-CH_3_), 27.8 (N-CH_3_). MS (ES^−^); 359.18 (M−H), HRMS (M-H); calcd for C_20_H_27_N_2_O_4_; 359.1976; found; 359.1965.

#### 3.4.8. Synthesis of Compound **7a**


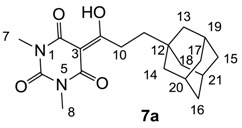


Yield; 50%; M.P.; 130 °C; ^1^H-NMR (400 MHz, CDCl_3_); 3.36 (s, 3H, C7), 3.33 (s, 3H, C8), 3.11–3.07 (m, 2H, C10), 1.98 (brs, 3H, C19–C21), 1.73–1.54 (m, 12H, C13–C18), 1.45–1.41 (m, 2H, C11). ^13^C-NMR (100 MHz, CDCl_3_); 201.2 (C9), 169.7 (C2), 160.8 (C4), 150.4 (C6), 95.2 (C3), 42.0 (C10), 40.0 (CH_2_), 37.0 (CH_2_), 32.4 (C12), 30.8 (CH_2_), 28.6 (N-CH_3_), 28.0 (CH), 27.8 (CH). MS (ES^−^); 345.19 (M−H), HRMS (M−H); calcd for C_19_H_25_N_2_O_4_; 345.1820; found; 345.1815.

#### 3.4.9. Synthesis of Compound **7b**


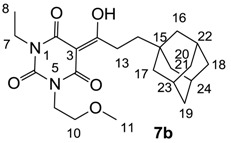


Yield; 44% (oil); ^1^H-NMR (400 MHz, CDCl_3_); 4.18–4.14 (m, 2H, C9), 4.02–3.95 (m, 2H, C7), 3.63–3.57 (m, 2H, C10), 3.34 and 3.33 (2 of s, 3H, C11), 3.11–3.07 (m, 2H, C13), 1.96 (brs, 3H, C22–C24), 1.71–1.61 (m, 6H, C16, C17 and C20), 1.52 (brs, 6H, C18, C19 and C21), 1.44–1.40 (m, 2H, C14), 1.26–1.18 (m, 3H, C8). ^13^C-NMR (100 MHz, CDCl_3_); 201.3 and 201.2 (C12), 169.7 and 169.5 (C2), 160.5 and 160.3 (C4), 149.8 and 149.7 (C6), 95.3 and 95.2 (C3), 69.4 and 69.2 (C10), 58.7 and 58.6 (C11), 42.0 (C16, C17 and C20), 40.1 and 40.0 (C9), 39.8 and 39.7 (C13), 37.0 (C18, C19 and C21), 36.7 and 36.6 (C7), 32.3 (C15), 30.8 and 30.7 (C14), 28.6 (C22–C24), 13.2 and 13.1 (C8). MS (ES^−^); 403.23 (M-H); MS (ES^+^); 427.21 (M+Na); HRMS (M+Na); calcd for C_22_H_32_N_2_Na_1_O_5_; 427.2203; found; 427.2191.

#### 3.4.10. Synthesis of Compound **8a**


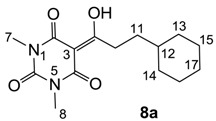


Yield; 25%; M.P.; 55 °C; ^1^H-NMR (400 MHz, CDCl_3_); 3.34 (s, 3H, C7), 3.31 (s, 3H, C8), 3.14–3.10 (m, 2H, C10), 1.76–1.62 (m, 5H, CH_2_), 1.58–1.52 (m, 2H, C11), 1.37–1.27 (m, 1H, C12), 1.23–1.11 (m, 3H, CH_2_), 0.98–0.89 (m, 2H, CH_2_). ^13^C-NMR (100 MHz, CDCl_3_); 200.3 (C9), 169.7 (C2), 160.7 (C4), 150.3 (C6), 95.1 (C3), 37.6 (C12), 34.4 (C10), 33.1 (C11), 32.9 (CH_2_), 28.0 (N-CH_3_), 27.7 (N-CH_3_), 26.5 (CH_2_), 26.2 (CH_2_). MS (ES^−^); 293.16 (M−H), HRMS (M−H); calcd for C_15_H_21_N_2_O_4_; 293.1507; found; 293.1510.

#### 3.4.11. Synthesis of Compound **8b**


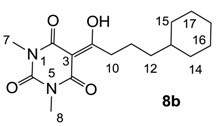


Yield; 53% (oil); ^1^H-NMR (400 MHz, CDCl_3_); 3.34 (s, 3H, C7), 3.30 (s, 3H, C8), 3.09 (t, 2H, *J* = 7.6 Hz, C10), 1.71–1.60 (m, 7H, C13 and CH_2_), 1.30–1.09 (m, 6H, CH_2_), 0.91–0.81 (m, 2H, CH_2_). ^13^C-NMR (100 MHz, CDCl_3_); 199.9 (C9), 169.7 (C2), 160.7 (C4), 150.3 (C6), 95.1 (C3), 37.3 (C13), 37.1 (CH_2_), 36.9 (CH_2_), 33.1 (CH_2_), 27.9 (N-CH_3_), 27.7 (N-CH_3_), 26.6 (CH_2_), 26.2 (CH_2_), 23.1 (CH_2_). MS (ES^−^); 307.16 (M−H); HRMS (M−H); calcd for C_16_H_23_N_2_O_4_; 307.1663; found; 307.1661.

#### 3.4.12. Synthesis of Compound **9**


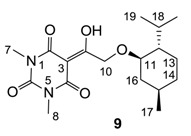


Yield; 84%; M.P.; 67 °C; ^1^H-NMR (400 MHz, CDCl_3_); 5.00 (d, 1H, *J* = 20.4 Hz, C10), 4.87 (d, 1H, *J* = 20.4 Hz, C10), 3.33 (s, 3H, C7), 3.27 (s, 3H, C8), 3.21–3.15 (m, 1H, C11), 2.29–2.21 (m, 1H, C15), 2.09–2.05 (m, 1H, C16), 1.62–1.57 (m, 2H, CH_2_), 1.35–1.27 (m, 2H, C12 and C18), 1.00–0.80 (m, 11H, C13, C14, C16, C19 and C20), 0.74 (d, 3H, *J* = 6.8 Hz, C17). ^13^C-NMR (100 MHz, CDCl_3_); 197.1 (C9), 169.4 (C2), 160.5 (C4), 150.1 (C6), 93.5 (C3), 80.5 (C11), 69.3 (C10), 47.9 (C12), 39.9 (C16), 34.3 (C14), 31.4 (N-CH_3_), 27.7 (N-CH_3_), 25.5 (C15), 23.2 (C13), 22.1 (C18), 20.8 (C19 and C20), 16.2 (C17). MS (ES^−^); 351.19 (M-H); MS (ES^+^); 375.22 (M+H); HRMS (M−H); calcd for C_18_H_27_N_2_O_5_; 351.1925; found; 351.1922.

#### 3.4.13. Synthesis of Compound (±)-**10**


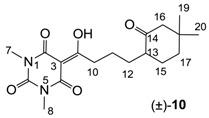


Yield; 51% (oil); ^1^H-NMR (500 MHz, CDCl_3_); 3.32 (s, 3H, C7), 3.28 (s, 3H, C8), 3.15–3.05 (m, 2H, C10), 2.25–2.21 (m, 1H, C13), 2.18 (d, 1H, *J* = 12.5 Hz, C16), 2.07 (d, 1H, *J* = 12.5 Hz, C16), 2.02–1.97 (m, 1H, C17), 1.88–1.81 (m, 1H, C12), 1.69–1.63 (m, 2H, C11), 1.62–1.43 (m, 3H, C15 and C17), 1.30–1.21 (m, 1H, C12), 1.01 (s, 3H, CH_3_), 0.83 (s, 3H, CH_3_). ^13^C-NMR (125 MHz, CDCl_3_); 212.4 (C14), 199.3 (C9), 169.6 (C2), 160.7 (C4), 150.3 (C6), 95.1 (C3), 54.8 (C16), 49.1 (C13), 37.9 (C10), 36.9 (C17), 36.6 (C18), 31.3 (CH_3_), 29.4 (C12 or C15), 28.8 (C12 or C15), 27.9 (N-CH_3_), 27.7 (N-CH_3_), 25.5 (CH_3_), 23.3 (C11). MS (ES^−^); 349.19 (M−H); MS (ES^+^); 351.21 (M+H), 373.19 (M+Na); HRMS (M+Na); calcd for C_18_H_26_N_2_Na_1_O_5_; 373.1734; found; 373.1723.

#### 3.4.14. Synthesis of Compound *cis*-**11**


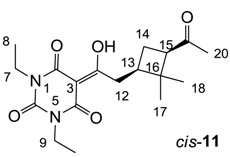


Yield; 46 % (oil); ^1^H-NMR (400 MHz, CDCl_3_); 3.99–3.91 (m, 4H, C7 and C9), 3.20 (dd, 1H, *J_1_* = 15.2 Hz, *J_2_* = 6.4 Hz, C12), 3.05 (dd, 1H, *J_1_* = 15.2 Hz, *J_2_* = 8.0 Hz, C12), 2.84 (dd, 1H, *J_1_* = 10.0 Hz, *J_2_* = 7.6 Hz, C15), 2.43–2.34 (m, 1H, C13), 2.11–2.03 (m, 1H, C14), 2.01 (s, 3H, C20), 1.92–1.85 (m, 1H, C14), 1.31 (s, 3H, C17), 1.23–1.16 (m, 6H, C8 and C10), 0.90 (s, 3H, C17). ^13^C NMR (100 MHz, CDCl_3_); 207.3 (C20), 198.8 (C11), 169.5 (C2), 160.4 (C4), 149.4 (C6), 95.2 (C3), 54.2 (C15), 43.7 (C16), 38.5 (C13), 37.3 (C12), 36.6 (NCH_2_), 36.5 (NCH_2_), 30.0 (C18 and C20), 23.2 (C14), 17.5 (C17), 13.1 (C8 and C10). MS (ES^−^); 349.18 (M−H); MS (ES^+^); 373.18 (M+Na); HRMS (M+Na); calcd for C_18_H_26_Na_1_N_2_O_5_; 373.1734; found; 373.1728.

#### 3.4.15. Synthesis of Compound (±)-**12**


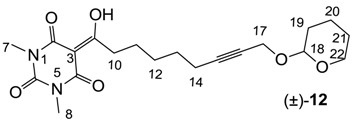


Yield; 35% (oil); ^1^H-NMR (400 MHz, CDCl_3_); 4.73 (brs, 1H, C18), 4.21 (d, 1H, *J* = 15.6 Hz, C17), 4.12 (d, 1H, *J* = 15.6 Hz, C17), 3.79–3.74 (m, 1H, C22), 3.47–3.43 (m, 1H, C22), 3.30 (s, 3H, C7), 3.25 (s, 3H, C8), 3.07 (t, 2H, *J* = 7.2 Hz, C10), 2.28–2.16 (m, 2H, C14), 1.78–1.45 (m, 10H, CH_2_). ^13^C-NMR (100 MHz, CDCl_3_); 199.3 (C9), 169.6 (C2), 160.6 (C4), 150.1 (C6), 96.3 (C18), 95.0 (C3), 86.0 (C15), 75.9 (C16), 61.7 (C22), 54.4 (C17), 36.4 (C10), 30.1 (CH_2_), 28.4 (CH_2_), 28.0 (CH_2_), 27.8 (N-CH_3_), 27.6 (N-CH_3_), 25.2 (CH_2_), 25.1 (CH_2_), 18.9 (CH_2_), 18.5 (CH_2_). MS (ES^−^); 391.19 (M−H); HRMS (M+Na); calcd for C_20_H_28_N_2_Na_1_O_6_; 415.1840; found; 415.1821.

#### 3.4.16. Synthesis of Compound **13**


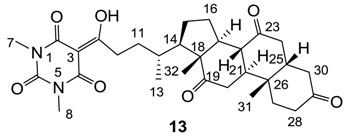


Yield; 87%; M.P.; 245 °C (decomposed); ^1^H-NMR (400 MHz, CDCl_3_); 3.35 (s, 3H, C7), 3.32 (s, 3H, C8), 3.20–3.05 (m, 2H), 2.94–2.81 (m, 3H), 2.37–1.81 (m, 15H), 1.70–1.57 (m, 2H), 1.39 (s, 3H, C32), 1.33–1.24 (m, 2H), 1.07 (s, 3H, C31), 0.92 (d, 3H, *J* = 6.4 Hz, C13). ^13^C-NMR (100 MHz, CDCl_3_); 211.8 (C=O), 209.0 (C=O), 208.7 (C=O), 200.1 (C=O), 169.7 (C2), 160.7 (C4), 150.3 (C6), 95.2 (C3), 56.9 (C18), 51.7 (CH), 48.9 (CH), 46.8 (CH), 45.7 (CH), 45.5 (CH), 44.9 (CH_2_), 42.7 (CH_2_), 38.6 (CH_2_), 36.4 (CH_2_), 36.3 (C12), 35.9 (C26), 35.2 (CH_2_), 34.3 (CH_2_), 33.9 (CH_2_), 31.5 (CH_2_), 28.0 (N-CH_3_), 27.8 (N-CH_3_), 27.6 (CH_2_), 25.6 (CH_2_), 25.1 (CH_2_), 24.9 (CH_2_), 21.8 (C13), 18.7 (C32), 11.8 (C31). MS (ES^−^); 539.30 (M−H), HRMS (M-H); calcd for C_30_H_39_N_2_O_7_; 539.2763; found; 539.2763.

#### 3.4.17. Synthesis of Compound **14**


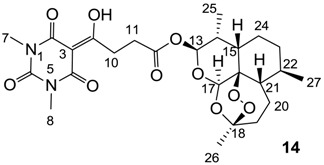


Yield; 66%; M.P. 96 °C; ^1^H-NMR (500 MHz, CDCl_3_); 5.79 (d, 1H, *J* = 10.0 Hz, C13), 5.42 (s, 1H, C17), 3.62–3.56 (m, 1H, C11), 3.50–3.44 (m, 1H, C11), 3.36 (s, 3H, C7), 3.32 (s, 3H, C8), 2.81 (t, 2H, *J* = 6.5 Hz, C10), 2.59–2.52 (m, 1H, C14), 2.36 (td, 1H, *J_1_* = 13.5 Hz, *J_2_* = 3.5 Hz, CH_2_), 2.05–2.00 (m, 1H, CH_2_), 1.91–1.85 (m, 1H, CH_2_), 1.79–1.69 (m, 2H, C24 and CH_2_), 1.63–1.59 (m, 1H, C15), 1.51–1.40 (m, 1H, CH_2_), 1.43 (s, 3H, C26), 1.38–1.24 (m, 3H, C21, C22 and C24), 1.04–0.99 (m, 1H, CH_2_), 0.95 (d, 3H, *J* = 6.0 Hz, C27), 0.85 (d, 3H, *J* = 7.0 Hz, C25). ^13^C-NMR (125 MHz, CDCl_3_); 197.4 (C9), 171.0 (C12), 169.7 (C2), 160.7 (C4), 150.2 (C6), 104.4 (C18), 95.2 (C3), 92.2 (C13), 91.4 (C17), 80.0 (C16), 51.5 (C21), 45.2 (C15), 37.2 (C22), 36.1 (CH_2_), 34.0 (CH_2_), 31.9 (C11), 31.8 (C14), 28.6 (C10), 27.9 (N-CH_3_), 27.8 (N-CH_3_), 25.9 (C26), 24.5 (CH_2_), 21.9 (C24), 20.2 (C27), 12.0 (C25). MS (ES^−^); 521.22 (M−H); HRMS (M−H); calcd for C_25_H_33_N_2_O_10_; 521.2141; found; 521.2148.

#### 3.4.18. Synthesis of Compound **15a**


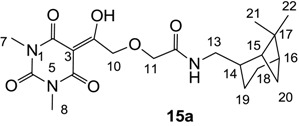


Yield; 27%; M.P.; 118 °C; ^1^H-NMR (400 MHz, CDCl_3_); 6.83 (brs, 1H, NH), 4.98 (s, 2H, CH_2_), 4.07 (s, 2H, CH_2_), 3.38 (s, 3H, C7), 3.34–3.28 (m, 5H, C8 and C13), 2.38–2.32 (m, 1H, C18), 2.16–2.18 (m, 1H, C14), 1.98–1.81 (m, 5H, C15, C16, C19 and C20), 1.54–1.44 (m, 1H, C19), 1.18 (s, 3H, C21), 1.03 (s, 3H, C22), 0.89 (d, 1H, *J* = 9.6 Hz, C18). ^13^C-NMR (100 MHz, CDCl_3_); 195.2 (C9), 169.5 (C12), 168.6 (C2), 160.4 (C4), 150.0 (C6), 93.9 (C3), 72.1 (CH_2_), 71.3 (CH_2_), 44.4 (C13), 43.7 (CH), 41.2 (CH), 41.2 (CH), 38.6 (C17), 33.1 (CH_2_), 28.0 (CH_3_), 27.9 (CH_3_), 27.8 (CH_3_), 25.9 (CH_2_), 23.1 (CH_3_), 19.7 (CH_2_). MS (ES^−^); 406.20 (M−H); HRMS (M−H); calcd for C_20_H_28_N_3_O_6_; 406.1984; found; 406.1986.

#### 3.4.19. Synthesis of Compound **15b**


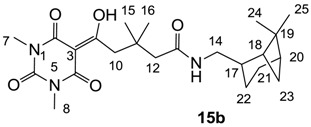


Yield; 43% (oil); ^1^H-NMR (500 MHz, CDCl_3_); 5.95 (brs, 1H, NH), 3.34–3.16 (m, 10H, C7, C8, C10 and C14), 2.30–2.27 (m, 1H, C21), 2.25 (s, 2H, C12), 2.17–2.10 (m, 1H, C17), 1.92–1.77 (m, 5H, C18, C20, C22 and C23), 1.47–1.41 (m, 1H, C22), 1.12 (s, 9H, CH_3_), 0.98 (s, 3H, CH_3_), 0.84 (d, 1H, *J* = 9.5 Hz, C21). ^13^C-NMR (125 MHz, CDCl_3_); 198.4 (C9), 170.7 (C13), 169.9 (C2), 160.8 (C4), 149.9 (C6), 96.8 (C3), 48.3 (CH_2_), 45.0 (CH_2_), 44.5 (CH_2_), 43.8 (CH), 41.3 (CH), 41.2 (CH), 38.5 (C19), 35.4 (C11), 33.0 (CH_2_), 28.1 (CH_3_), 28.1 (CH_3_), 27.8 (CH_3_), 25.8 (CH_2_), 23.1 (CH_3_), 19.8 (CH_2_). MS (ES^−^); 432.24 (M−H); MS (ES^+^); 434.29 (M+H), 456.27 (M+Na); HRMS (M+H); calcd for C_23_H_36_N_3_O_5_; 434.2649; found; 434.2634.

#### 3.4.20. Synthesis of Compound **16**


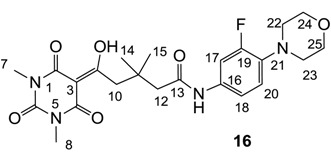


Yield; 49%; M.P.; 102 °C; ^1^H-NMR (500 MHz, CDCl_3_); 8.39 (brs, 1H, ArH), 7.39 (brs, 1H, ArH), 7.14 (d, 1H, *J* = 7.5 Hz, C20), 3.98 (brs, 4H, C24 and C25), 3.43 (s, 2H, C10), 3.37 (s, 3H, C7), 3.31 (s, 3H, C8), 3.21 (brs, 4H, C22 and C23), 2.43 (s, 2H, C12), 1.22 (s, 6H, C14 and C15). ^13^C-NMR (125 MHz, CDCl_3_); 198.2 (C9), 170.0 (C2), 162.7 (C13), 162.0 (C4), 155.5 (d, *J_C-F_* = 245 Hz, C19), 149.9 (C6), 135.6 (C21), 120.3 (C16), 115.5 (Ar-*tert* C), 115.5 (Ar-*tert* C), 109.0 (d, *J_C-F_* = 25.5 Hz, C17), 97.0 (C3), 66.0 (C24 and C25), 51.8 (C22 and C23), 48.2 (CH_2_), 44.4 (CH_2_), 36.0 (C11), 28.3 (C14 and C15), 28.2 (N-CH_3_), 28.0 (N-CH_3_). MS (ES^−^); 475.20 (M−H), MS (ES^+^); 499.23 (M+Na), HRMS (M+Na); calcd for C_23_H_29_F_1_N_4_Na_1_O_6_; 499.1963; found; 499.1964.

#### 3.4.21. Synthesis of Compound **17a**


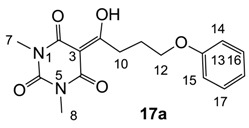


Yield; 84%; M.P.; 95 °C; ^1^H-NMR (500 MHz, CDCl_3_); 7.27–7.23 (m, 2H, C16 and C17), 6.91 (t, 1H, *J* = 7.0 Hz, C18), 6.85 (d, 2H, *J* = 8.0 Hz, C14 and C15), 4.05 (t, 2H, *J* = 6.0 Hz, C12), 3.37–3.34 (m, 5H, C10 and C7), 3.29 (s, 3H, C8), 2.23–2.17 (m, 2H, C11). ^13^C-NMR (125 MHz, CDCl_3_); 198.8 (C9), 169.6 (C2), 160.7 (C4), 158.6 (C13), 150.1 (C6), 129.2 (C16 and C17), 120.6 (C18), 114.3 (C14 and C15), 95.3 (C3), 66.7 (C12), 33.6 (C10), 27.8 (N-CH_3_), 27.7 (N-CH_3_), 25.1 (C11). MS (ES^−^); 317.13 (M−H), HRMS (M−H); calcd for C_16_H_17_N_2_O_5_; 317.1143; found; 317.1141.

#### 3.4.22. Synthesis of Compound **17b**


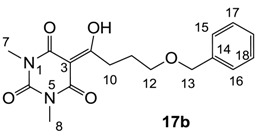


Yield; 63%; M.P.; 84 °C; ^1^H-NMR (400 MHz, CDCl_3_); 7.34–7.23 (m, 5H, C15-C19), 4.49 (s, 2H, C13), 3.58 (t, 2H, *J* = 6.0 Hz, C12), 3.33 (s, 3H, C7), 3.31 (s, 3H, C8), 3.27 (t, 2H, *J* = 6.0 Hz, C10), 2.07–2.00 (m, 2H, C11). ^13^C-NMR (100 MHz, CDCl_3_); 199.4 (C9), 169.6 (C2), 160.8 (C4), 150.2 (C6), 138.3 (C14), 128.2 (C17 and C18), 127.5 (C15 and C16), 127.4 (C19), 95.2 (C3), 72.8 (C13), 69.4 (C12), 33.8 (C10), 27.9 (N-CH_3_), 27.7 (N-CH_3_), 25.7 (C11). MS (ES^−^); 331.13 (M−H); MS (ES^+^); 355.14 (M+Na); HRMS (M+Na); calcd for C_17_H_20_N_2_Na_1_O_5_; 355.1264; found; 355.1262.

#### 3.4.23. Synthesis of Compound **17c**


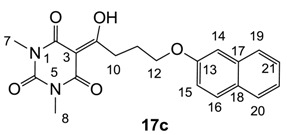


Yield; 70%; M.P.; 142 °C; ^1^H-NMR (400 MHz, CDCl_3_); 7.77–7.70 (m, 3H, C16, C19 and C20), 7.44 (dd, 1H, *J_1_=J_2_* = 6.8 Hz, C21), 7.33 (dd, 1H, *J_1_=J_2_* = 6.8 Hz, C22), 7.12–7.11 (m, 2H, C14 and C15), 4.20 (t, 2H, *J* = 5.6 Hz, C12), 3.41 (t, 2H, *J* = 5.6 Hz, C10), 3.36 (s, 3H, C7), 3.29 (s, 3H, C8), 2.31–2.25 (m, 2H, C11). ^13^C-NMR (100 MHz, CDCl_3_); 198.9 (C9), 169.7 (C2), 160.8 (C4), 156.6 (C13), 150.2 (C6), 134.5 (quart C), 129.3 (tert C), 128.9 (quart C), 127.6 (tert C), 126.6 (tert C), 126.3 (tert C), 123.6 (tert C), 118.8 (tert C), 95.4 (C3), 66.9 (C12), 33.7 (C10), 27.9 (N-CH_3_), 27.8 (N-CH_3_), 25.2 (C11). MS (ES^−^); 367.14 (M−H); MS (ES^+^); 369.19 (M+H), 391.14 (M+Na); HRMS (M+Na); calcd for C_20_H_20_N_2_Na_1_O_5_; 391.1264; found; 391.1261.

#### 3.4.24. Synthesis of Compound **18a**


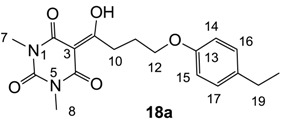


Yield; 75%; M.P.; 87 °C; ^1^H-NMR (400 MHz, CDCl_3_); 7.09 (d, 2H, *J* = 8.0 Hz, C16 and C17), 6.80 (d, 2H, *J* = 8.0 Hz, C14 and C15), 4.05 (t, 2H, *J* = 6.0 Hz, C12), 3.38–3.34 (m, 5H, C7 and C10), 3.31 (s, 3H, C8), 2.58 (q, 2H, *J* = 7.6 Hz, C19), 2.23–2.17 (m, 2H, C11), 1.21 (t, 3H, *J* = 7.6 Hz, C20). ^13^C-NMR (100 MHz, CDCl_3_); 199.0 (C9), 169.7 (C2), 160.8 (C4), 156.7 (C13), 150.3 (C6), 130.5 (C18), 128.6 (C16 and C17), 114.3 (C14 and C15), 95.4 (C3), 66.9 (C12), 33.7 (C10), 28.0 (N-CH_3_), 27.9 (C19), 27.8 (N-CH_3_), 25.3 (C11), 15.8 (C20). MS (ES^−^); 345.15 (M−H); MS (ES^+^); 369.16 (M+Na); HRMS (M+Na); calcd for C_18_H_22_N_2_Na_1_O_5_; 369.1421; found; 369.1412.

#### 3.4.25. Synthesis of Compound **18b**


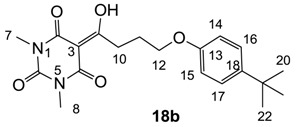


Yield; 58%; M.P.; 92 °C; ^1^H-NMR (400 MHz, CDCl_3_); 7.29 (d, 2H, *J* = 8.8 Hz, C16 and C17), 6.81 (d, 2H, *J* = 8.8 Hz, C14 and C15), 4.06 (t, 2H, *J* = 6.4 Hz, C12), 3.39–3.35 (m, 5H, C7 and C10), 3.31 (s, 3H, C8), 2.24–2.17 (m, 2H, C11), 1.30 (s, 9H, C20–C22). ^13^C-NMR (100 MHz, CDCl_3_); 199.0 (C9), 169.7 (C2), 160.8 (C4), 156.4 (C13), 150.2 (C6), 143.4 (C18), 126.1 (C16 and C17), 113.4 (C14 and C15), 95.4 (C3), 66.9 (C12), 34.0 (C19), 33.7 (C10), 31.5 (C20–C22), 27.9 (N-CH_3_), 27.8 (N-CH_3_), 25.3 (C11). MS (ES^−^); 373.19 (M−H); MS (ES^+^); 397.20 (M+Na); HRMS (M+Na); calcd for C_20_H_26_N_2_Na_1_O_5_; 397.1734; found; 397.1740.

#### 3.4.26. Synthesis of Compound **18c**


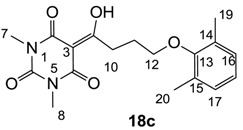


Yield; 67%; M.P.; 150 °C; ^1^H-NMR (400 MHz, CDCl_3_); 7.00 (d, 2H, *J* = 7.6 Hz, C16 and C17), 6.91 (dd, 1H, *J_1_* = 7.6 Hz, *J_2_*= 7.6 Hz, C18), 3.87 (t, 2H, *J* = 6.4 Hz, C12), 3.44 (t, 2H, *J* = 6.4 Hz, C10), 3.39 (s, 3H, C7), 3.35 (s, 3H, C8), 2.29 (s, 6H, C19 and C20), 2.25–2.18 (m, 2H, C11). ^13^C-NMR (100 MHz, CDCl_3_); 199.1 (C9), 169.7 (C2), 160.8 (C4), 155.7 (C13), 150.3 (C6), 130.8 (C14 or C15), 128.8 (C16 and C17), 123.7 (C18), 95.3 (C3), 70.9 (C12), 33.7 (C10), 28.0 (N-CH_3_), 27.8 (N-CH_3_), 26.1 (C11), 16.3 (C19 and C20). MS (ES^−^); 345.16 (M−H); MS (ES^+^); 347.20 (M+H), 369.17 (M+Na); HRMS (M+Na); calcd for C_18_H_22_N_2_Na_1_O_5_; 369.1421; found; 369.1415.

#### 3.4.27. Synthesis of Compound **18d**


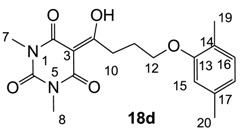


Yield; 72%; M.P.; 139 °C; ^1^H-NMR (400 MHz, CDCl_3_); 7.00 (d, 1H, *J* = 7.2 Hz, C16), 6.67 (d, 1H, *J* = 7.2 Hz, C18), 6.63 (s, 1H, C15), 4.07 (t, 2H, *J* = 6.0 Hz, C12), 3.42–3.38 (m, 5H, C7 and C10), 3.33 (s, 3H, C8), 2.32 (s, 6H, C20), 2.27–2.20 (m, 2H, C11), 2.17 (s, 6H, C19). ^13^C-NMR (100 MHz, CDCl_3_); 199.0 (C9), 169.7 (C2), 160.8 (C4), 156.6 (C13), 150.3 (C6), 136.4 (C17), 130.3 (C16), 123.5 (C14), 120.8 (C18), 111.8 (C15), 95.3 (C3), 66.7 (C12), 33.7 (C10), 27.9 (N-CH_3_), 27.8 (N-CH_3_), 25.3 (C11), 21.3 (C20), 15.7 (C19). MS (ES^−^); 345.15 (M−H); MS (ES^+^); 347.18 (M+H), 369.16 (M+Na); HRMS (M+Na); calcd for C_18_H_22_N_2_Na_1_O_5_; 369.1421; found; 369.1407.

#### 3.4.28. Synthesis of Compound **18e**


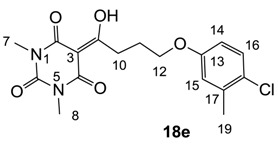


Yield; 43%; M.P.; 127 °C; ^1^H-NMR (400 MHz, CDCl_3_); 7.17 (d, 1H, *J* = 8.8 Hz, C16), 6.72 (d, 1H, *J* = 2.8 Hz, C15), 6.62 (dd, 1H, *J_1_* = 8.8 Hz, *J_2_*= 2.8 Hz, C14), 4.01 (t, 2H, *J* = 6.0 Hz, C12), 3.36–3.29 (m, 8H, C7, C8 and C10), 2.30 (s, 3H, C19), 2.21–2.14 (m, 2H, C11). ^13^C-NMR (100 MHz, CDCl_3_); 198.7 (C9), 169.7 (C2), 160.7 (C4), 157.2 (C13), 150.2 (C6), 136.8 (C17 or C18), 129.4 (C16), 125.7 (C17 or C18), 116.9 (C14 or C15), 112.9 (C14 or C15), 95.3 (C3), 67.1 (C12), 35.6 (C10), 27.9 (N-CH_3_), 27.8 (N-CH_3_), 25.1 (C11), 20.2 (C19). MS (ES^−^); 365.11 (M−H), HRMS (M+Na); calcd for C_17_H_19_Cl_1_N_2_Na_1_O_5_; 389.0875; found; 389.0863.

#### 3.4.29. Synthesis of Compound **18f**


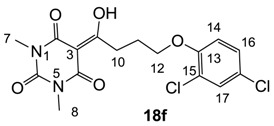


Yield; 70%; M.P.; 123 °C; ^1^H-NMR (400 MHz, CDCl_3_); 7.31 (s, 1H, C17), 7.14 (d, 1H, *J* = 8.8 Hz, C16), 6.81 (d, 1H, *J* = 8.8 Hz, C14), 4.09 (t, 2H, *J* = 6.0 Hz, C12), 3.39–3.35 (m, 5H, C10 and C7), 3.29 (s, 3H, C8), 2.28–2.21 (m, 2H, C11). ^13^C-NMR (100 MHz, CDCl_3_); 198.5 (C9), 169.7 (C2), 160.8 (C4), 153.0 (C13), 150.2 (C6), 129.8 (C17), 127.4 (C16), 125.6 (C18), 123.6 (C15), 113.8 (C14), 95.4 (C3), 68.4 (C12), 33.5 (C10), 27.9 (N-CH_3_), 27.8 (N-CH_3_), 24.9 (C11). MS (ES^−^); 385.06 (M−H), HRMS (M−H); calcd for C_16_H_15_Cl_2_N_2_O_5_; 385.0364; found; 385.0368.

#### 3.4.30. Synthesis of Compound **18g**


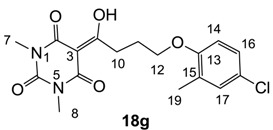


Yield; 56%; M.P.; 148 °C; ^1^H-NMR (400 MHz, CDCl_3_); 7.09–7.07 (m, 2H, C16 and C17), 6.70 (d, 1H, *J* = 8.0 Hz, C14), 4.04 (t, 2H, *J* = 6.0 Hz, C12), 3.40–3.36 (m, 5H, C10 and C7), 3.33 (s, 3H, C8), 2.26–2.20 (m, 2H, C11), 2.18 (s, 3H, C19). ^13^C-NMR (100 MHz, CDCl_3_); 198.8 (C9), 169.8 (C2), 160.8 (C4), 155.4 (C13), 150.3 (C6), 130.3 (C17), 128.7 (C15), 126.2 (C16), 125.0 (C18), 111.8 (C14), 95.4 (C3), 67.2 (C12), 33.7 (C10), 28.0 (N-CH_3_), 27.8 (N-CH_3_), 25.2 (C11), 16.0 (C19). MS (ES^−^); 365.10 (M−H), HRMS (M−H); calcd for C_17_H_18_Cl_1_N_2_O_5_; 365.0910; found; 365.0901.

#### 3.4.31. Synthesis of Compound **19a**


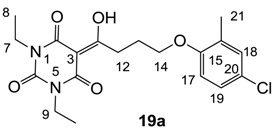


Yield; 37%; M.P. 77 °C; ^1^H-NMR (400 MHz, CDCl_3_); 7.07–7.05 (m, 2H, C18 and C19), 6.69 (d, 1H, *J* = 8.4 Hz, C17), 4.04–3.94 (m, 6H, C7, C9 and C14), 3.37 (t, 2H, *J* = 7.2 Hz, C12), 2.25–2.18 (m, 2H, C13), 2.17 (s, 3H, C21), 1.25 (t, 3H, *J* = 7.6 Hz, CH_3_), 1.20 (t, 3H, *J* = 7.6 Hz, CH_3_). ^13^C-NMR (100 MHz, CDCl_3_); 198.9 (C11), 169.5 (C2), 160.4 (C4), 155.4 (C15), 149.3 (C6), 130.3 (C18), 128.6 (quart C), 126.2 (C19), 124.9 (quart C), 111.7 (C17), 95.4 (C3), 67.1 (C14), 36.7 (N-CH_2_), 36.5 (N-CH_2_), 33.7 (C12), 25.0 (C13), 16.0 (C21), 13.2 (CH_3_), 13.1 (CH_3_). MS (ES^−^); 393.12 (M−H); HRMS (M−H); calcd for C_19_H_22_Cl_1_N_2_O_5_; 393.1223; found; 393.1215.

#### 3.4.32. Synthesis of Compound **19b**


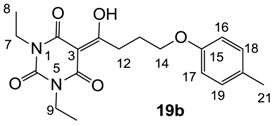


Yield; 40% (oil); ^1^H-NMR (400 MHz, CDCl_3_); 7.04 (d, 2H, *J* = 8.4 Hz, C18 and C19), 6.76 (d, 2H, *J* = 8.4 Hz, C17 and C16), 4.05–3.93 (m, 6H, C7, C9 and C14), 3.35 (t, 2H, *J* = 7.2 Hz, C12), 2.27 (s, 3H, C21), 2.23–2.16 (m, 2H, C13), 1.28–1.17 (m, 6H, C8 and C10). ^13^C-NMR (100 MHz, CDCl_3_); 199.1 (C11), 169.5 (C2), 160.4 (C4), 156.5 (C15), 149.3 (C6), 129.8 (C20), 129.7 (C18 and C19), 114.1 (C16 and C17), 95.4 (C3), 66.9 (C14), 36.6 (N-CH_2_), 36.5 (N-CH_2_), 33.7 (C12), 25.2 (C13), 20.3 (C21), 13.1 (CH_3_), 13.0 (CH_3_). MS (ES^−^); 359.17 (M−H); HRMS (M−H); calcd for C_19_H_23_N_2_O_5_; 359.1612; found; 359.1611.

#### 3.4.33. Synthesis of Compound **20**


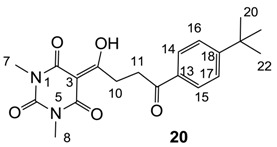


Yield; 15% (oil); ^1^H-NMR (400 MHz, CDCl_3_); 7.93 (d, 2H, *J* = 8.4 Hz, C16 and C17), 7.49 (d, 2H, *J* = 8.4 Hz, C14 and C15), 3.63 (t, 2H, *J* = 6.0 Hz, C11), 3.41 (t, 2H, *J* = 6.0 Hz, C10), 3.37 (s, 3H, C7), 3.34 (s, 3H, C8), 1.34 (s, 9H, C20–C22). ^13^C-NMR (100 MHz, CDCl_3_); 198.6 (C9), 197.7 (C12), 169.7 (C2), 160.9 (C4), 157.0 (C18), 150.3 (C6), 133.7 (C13), 128.0 (C14 and C15), 125.5 (C16 and C17), 95.2 (C3), 35.1 (C19), 32.9 (CH_2_), 31.3 (CH_2_), 31.0 (C20-C22), 28.0 (N-CH_3_), 27.8 (N-CH_3_). MS (ES^−^); 371.17 (M−H), HRMS (M+Na); calcd for C_20_H_24_N_2_Na_1_O_5_; 395.1577; found; 395.1567.

#### 3.4.34. Synthesis of Compound **21**


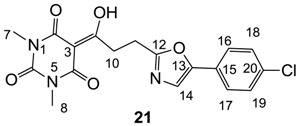


Yield; 70%; M.P.; 173 °C; ^1^H-NMR (400 MHz, CDCl_3_); 7.52 (d, 2H, *J* = 8.0 Hz, C16 and C17), 7.36 (d, 2H, *J* = 8.0 Hz, C18 and C19), 7.20 (s, 1H, C14), 3.73 (t, 2H, *J* = 7.2 Hz, C11), 3.37 (s, 3H, C7), 3.32 (s, 3H, C8), 3.24 (t, 2H, *J* = 7.2 Hz, C10). ^13^C-NMR (100 MHz, CDCl_3_); 196.9 (C9), 169.7 (C2), 162.6 (quart C), 160.7 (C4), 150.3 (C6), 150.2 (quart C), 133.9 (C20), 129.1 (C18 and C19), 126.5 (C15), 125.2 (C16 and C17), 122.3 (C14), 95.4 (C3), 33.9 (C10), 28.0 (N-CH_3_), 27.9 (N-CH_3_), 23.2 (C11). MS (ES^−^); 388.08 (M−H), HRMS (M−H); calcd for C_18_H_15_Cl_1_N_3_O_5_; 388.0706; found; 388.0689.

#### 3.4.35. Synthesis of Compound **22**


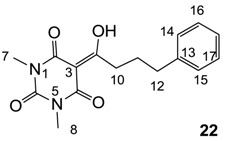


Yield; 69% (oil); ^1^H-NMR (400 MHz, CDCl_3_); 7.31–7.27 (m, 2H, C16 and C17), 7.22–7.18 (m, 3H, C14, C15 and C18), 3.37 (s, 3H, C7), 3.34 (s, 3H, C8), 3.20 (t, 2H, *J* = 8.0 Hz, C10), 2.75 (t, 2H, *J* = 8.0 Hz, C10), 2.08–2.01 (m, 2H, C11). ^13^C-NMR (100 MHz, CDCl_3_); 199.3 (C9), 169.7 (C2), 160.8 (C4), 150.3 (C6), 141.3 (C13), 128.4 (C16 and C17), 128.3 (C14 and C15), 126.0 (C18), 95.3 (C3), 36.3 (C10), 35.5 (C12), 28.0 (N-CH_3_), 27.8 (N-CH_3_), 27.2 (C11). MS (ES^−^); 301.13 (M−H), HRMS (M−H); calcd for C_16_H_17_N_2_O_4_; 301.1194; found; 301.1193.

#### 3.4.36. Synthesis of Compound **23a**


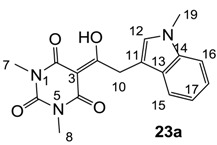


Yield; 20%; M.P.; 156 °C; ^1^H-NMR (400 MHz, CDCl_3_); 7.77 (d, 1H, *J* = 8.0 Hz, C15), 7.31–7.15 (m, 4H, C12 and C16-C18), 4.62 (s, 2H, C10), 3.77 (s, 3H, C19), 3.39 (s, 3H, C7), 3.33 (s, 3H, C8). ^13^C-NMR (100 MHz, CDCl_3_); 196.7 (C9), 169.8 (C2), 160.7 (C4), 150.2 (C6), 136.7 (C14), 128.6 (C12), 127.8 (C13), 121.7 (C18), 119.3 (C15 or C17), 119.2 (C15 or C17), 109.2 (C16), 106.5 (C11), 94.6 (C3), 32.6 (C19), 32.2 (C10), 28.0 (N-CH_3_), 27.7 (N-CH_3_). MS (ES^−^); 326.13 (M−H), HRMS (M+Na); calcd for C_17_H_17_N_3_Na_1_O_4_; 350.1111; found; 350.1099.

#### 3.4.37. Synthesis of Compound **23b**


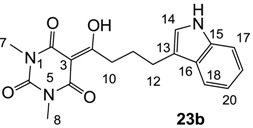


Yield; 57%; M.P.; 182 °C; ^1^H-NMR (400 MHz, CDCl_3_); 7.97 (brs, 1H, NH), 7.62 (d, 1H, *J* = 8.0 Hz, C18), 7.35 (d, 1H, *J* = 8.4 Hz, C17), 7.19 (dd, 1H, *J_1_* = *J_2_* = 8.0 Hz, C19 or C20), 7.12 (dd, 1H, *J_1_* = *J_2_* = 8.0 Hz, C19 or C20), 7.04 (s, 1H, C14), 3.36 (s, 3H, C7), 3.33 (s, 3H, C8), 3.26 (t, 2H, *J* = 7.6 Hz, C10), 2.91 (t, 2H, *J* = 7.6 Hz, C12), 2.19–2.12 (m, 2H, C11). ^13^C-NMR (100 MHz, CDCl_3_); 199.7 (C9), 169.7 (C2), 160.8 (C4), 150.4 (C6), 136.3 (C15), 127.4 (C16), 121.9 (C19 or C20), 121.5 (C19 or C20), 119.2 (C17 or C18), 118.9 (C17 or C18), 115.6 (C13), 111.0 (C14), 95.3 (C3), 36.6 (C10), 28.0 (N-CH_3_), 27.8 (N-CH_3_), 26.0 (C12), 24.9 (C11). MS (ES^−^); 340.14 (M−H), HRMS (M−H); calcd for C_18_H_18_N_3_O_4_; 340.1303; found; 340.1295.

#### 3.4.38. Synthesis of Compound **24**


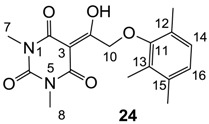


Yield; 77%; M.P.; 184 °C; ^1^H-NMR (400 MHz, CDCl_3_); 6.91 (d, 1H, *J* = 7.6 Hz, C14), 6.86 (d, 1H, *J* = 7.6 Hz, C16), 5.24 (s, 2H, C10), 3.42 (s, 3H, C7), 3.30 (s, 3H, C8), 2.26 (s, 3H, CH_3_), 2.23 (s, 3H, CH_3_), 2.20 (s, 3H, CH_3_). ^13^C-NMR (100 MHz, CDCl_3_); 194.7 (C9), 169.6 (C2), 160.5 (C4), 155.0 (C11), 150.1 (C6), 136.0 (quart C), 129.2 (quart C), 127.9 (C14), 127.8 (quart C), 125.8 (C16), 93.6 (C3), 72.5 (C10), 27.9 (N-CH_3_), 27.8 (N-CH_3_), 19.8 (CH_3_), 16.1 (CH_3_), 12.3 (CH_3_). MS (ES^−^); 331.15 (M−H), HRMS (M+Na); calcd for C_17_H_20_N_2_Na_1_O_5_; 355.1264; found; 355.1271.

#### 3.4.39. Synthesis of Compound **25a**


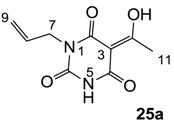


Yield; 58%; M.P.; 181 °C; Mixture of two exo-enol tautomers (E1: E2 = 40: 60); ^1^H-NMR (500 MHz, CDCl_3_); 8.84 (brs, 1H, NH E1), 8.54 (brs, 1H, NH E2), 5.92–5.83 (m, 1H, C8), 5.32–5.20 (m, 2H, C9), 4.53–4.50 (m, 2H, C7), 2.73 (s, 3H, C11). ^13^C-NMR (125 MHz, CDCl_3_); 196.9 (C10 E1), 196.4 (C10 E2), 170.1 (C2 E2), 169.0 (C4 E1), 161.2 (C2 E1), 161.0 (C4 E2), 149.0 (C6 E1), 148.8 (C6 E2), 131.5 (C8 E1), 130.8 (C8 E2), 118.8 (C9 E2), 118.1 (C9 E1), 95.7 (C3 E2), 95.6 (C3 E1), 42.8 (C7 E1), 42.7 (C7 E2), 24.5 (C11 E1), 24.4 (C11 E2). MS (ES^−^); 209.07 (M−H); HRMS (M−H); calcd for C_9_H_9_N_2_O_4_; 209.0568; found; 209.0570.

#### 3.4.40. Synthesis of Compound (±)-**25b**


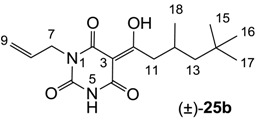


Yield; 40%; M.P.; 76 °C; Mixture of two exo-enol tautomers (E1:E2 = 40:60); ^1^H-NMR (400 MHz, CDCl_3_); 9.94 (brs, 1H, NH E1), 9.39 (brs, 1H, NH E2), 5.92–5.81 (m, 1H, C8), 5.30–5.16 (m, 2H, C9), 4.53–4.50 (m, 2H, C7), 3.09–3.07 (m, 2H, C11), 2.11–2.13 (m, 1H, C12), 1.36 (dd, 1H, *J_1_* = 6.4 Hz, *J_2_* = 6.0 Hz, C13 E1), 1.33 (dd, 1H, *J_1_* = 6.0 Hz, *J_2_* = 4.0 Hz, C13 E2), 1.19 (dd, 1H, *J_1_* = 6.0 Hz, *J_2_* = 4.0 Hz, C13 E2), 1.16 (dd, 1H, *J_1_* = 6.4 Hz, *J_2_* = 6.0 Hz, C13 E1), 1.02–1.00 (m, 3H, C18), 0.89 (s, 9H, C15-C17). ^13^C-NMR (125 MHz, CDCl_3_); 200.1 (C10 E1), 199.5 (C10 E2), 170.4 (C2 E2), 169.7 (C4 E1), 161.2 (C2 E1), 160.9 (C4 E2), 149.6 (C6 E1), 149.0 (C6 E2), 131.6 (C8 E1), 130.9 (C8 E2), 118.6 (C9 E2), 117.8 (C9 E1), 95.7 (C3 E2), 95.6 (C3 E1), 50.6 (CH_2_ E2), 50.5 (CH_2_ E1), 45.2 (CH_2_ E2), 45.1 (CH_2_ E1), 42.6 (C7 E1), 42.5 (C7 E2), 31.0 (C14), 29.9 (C15-C17), 28.1 (CH_3_ E2), 27.8 (CH_3_ E1), 22.7 (CH_3_ E1), 22.6 (CH_3_ E2). MS (ES^−^); 307.19 (M−H); HRMS (M−H); calcd for C_16_H_23_N_2_O_4_; 307.1663; found; 307.1657.

#### 3.4.41. Synthesis of Compound **26a**


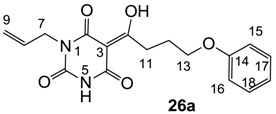


Yield; 56%; M.P.; 131 °C; Mixture of two exo-enol tautomers (E1: E2 = 40: 60); ^1^H-NMR (500 MHz, CDCl_3_); 9.36 (brs, 1H, NH E1), 8.90 (brs, 1H, NH E2), 7.29–7.25 (m, 2H, C17 and C18), 6.96–6.92 (m, 1H, C19), 6.87 (d, 2H, *J* = 8.0 Hz, C15 and C16), 5.93–5.80 (m, 1H, C8), 5.33–5.18 (m, 2H, C9), 4.53 (d, 2H, *J* = 6.0 Hz, C7 E2), 4.48 (d, 2H, *J* = 6.0 Hz, C7 E1), 4.09–4.06 (m, 2H, C13), 3.38 (t, 2H, *J* = 7.5 Hz, C11), 2.25–2.20 (m, 2H, C12). ^13^C-NMR (125 MHz, CDCl_3_); 199.9 (C10 E1), 199.3 (C10 E2), 170.4 (C2 E2), 169.5 (C4 E1), 160.9 (C4 E2 and C2 E1), 158.6 (C14), 149.2 (C6 E1), 148.8 (C6 E2), 131.5 (C8 E1), 130.8 (C8 E2), 129.4 (C17 and C18 E1), 129.4 (C17 and C18 E2), 120.7 (C19), 118.8 (C9 E2), 118.0 (C9 E1), 114.4 (C15 and C16), 95.2 (C3 E2), 95.2 (C3 E1), 66.7 (C13), 42.7 (C7 E2), 42.7 (C7 E1), 33.6 (C11 E1), 33.5 (C11 E2), 25.3 (C12 E2), 25.1 (C12 E1). MS (ES^−^); 329.11 (M−H); HRMS (M−H); calcd for C_17_H_17_N_2_O_5_; 329.1143; found; 329.1143.

#### 3.4.42. Synthesis of Compound **26b**


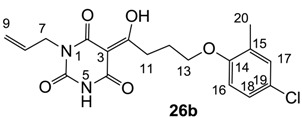


Yield; 59%; M.P.; 128 °C; Mixture of two exo-enol tautomers (E1: E2 = 40: 60); ^1^H-NMR (400 MHz, CDCl_3_); 9.56 (brs, 1H, NH E1), 9.10 (brs, 1H, NH E2), 7.09–7.07 (m, 2H, C17 and C18), 6.71 (d, 1H, *J* = 8.4 Hz, C16), 5.93–5.80 (m, 1H, C8), 5.32–5.18 (m, 2H, C9), 4.53 (d, 2H, *J* = 5.6 Hz, C7 E2), 4.50 (d, 2H, *J* = 5.6 Hz, C7 E1), 4.04 (t, 2H, *J* = 6.0 Hz, C11), 3.38 (t, 2H, *J* = 7.6 Hz, C13), 2.26–2.19 (m, 2H, C12), 2.18 (s, 3H, C20 E1), 2.17 (s, 3H, C20 E2). ^13^C-NMR (100 MHz, CDCl_3_); 199.7 (C10 E1), 199.1 (C10 E2), 170.4 (C2 E2), 169.5 (C4 E1), 161.1 (C4 E2), 160.9 (C2 E1), 155.4 (C14), 149.3 (C6 E1), 148.8 (C6 E2), 131.5 (C8 E1), 130.8 (C8 E2), 130.3 (C17), 128.6 (quart C), 126.2 (C18), 125.0 (quart C), 118.9 (C9 E2), 118.1 (C9 E1), 111.8 (C16), 95.2 (C3), 66.1 (C13 E2), 67.1 (C13 E1), 42.7 (C7), 33.6 (C11 E1), 33.5 (C11 E2), 25.2 (C12 E2), 24.9 (C12 E1), 16.0 (C20). MS (ES^−^); 377.11 (M−H); HRMS (M−H); calcd for C_18_H_18_Cl_1_N_2_O_5_; 377.0910; found; 377.0908.

#### 3.4.43. Synthesis of Compound **26c**


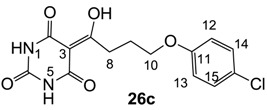


Yield; 37%; M.P.; 201 °C; ^1^H-NMR (500 MHz, DMSO); 11.86 (brs, 1H, NH), 11.05 (brs, 1H, NH), 7.30 (d, 2H, *J* = 9.0 Hz, C14 and C15), 6.93 (d, 2H, *J* = 9.0 Hz, C12 and C13), 4.02 (t, 2H, *J* = 6.5 Hz, C10), 3.20 (t, 2H, *J* = 6.5 Hz, C8), 2.07–2.01 (m, 2H, C9). ^13^C-NMR (125 MHz, DMSO); 197.9 (C7), 157.3 (C11), 149.0 (C2, C4 and C6), 129.2 (C14 and C15), 124.2 (C16), 116.2 (C12 and C13), 94.9 (C3), 67.2 (C10), 32.8 (C8), 24.4 (C9). MS (ES^−^); 323.07 (M−H); HRMS (M−H); calcd for C_14_H_12_N_2_O_5_; 323.0440; found; 323.0436.

#### 3.4.44. Synthesis of Compound **26d**


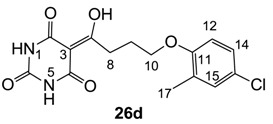


Yield; 44%; M.P.; 207 °C; ^1^H-NMR (400 MHz, DMSO); 11.86 (brs, 1H, NH), 11.06 (brs, 1H, NH), 7.17–7.14 (m, 2H, C14 and C15), 6.91 (d, 1H, *J* = 8.8 Hz, C12), 4.01 (t, 2H, *J* = 6.0 Hz, C10), 3.24 (t, 2H, *J* = 6.0 Hz, C8), 2.12–2.03 (m, 5H, C9 and C17). ^13^C-NMR (125 MHz, DMSO); 198.0 (C7), 155.4 (C11), 149.1 (C2, C4 and C6), 129.9 (C15), 128.3 (tert-C), 126.5 (C14), 123.7 (tert-C), 112.7 (C12), 94.9 (C3), 67.2 (C10), 32.9 (C8), 24.5 (C9), 15.6 (C17). MS (ES^−^); 337.08 (M−H); HRMS (M−H); calcd for C_15_H_14_N_2_O_5_; 337.0597; found; 337.0587.

#### 3.4.45. Synthesis of Compound **27**


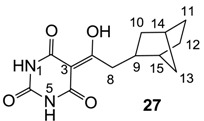


Yield; 50%; M.P.; over 260 °C; ^1^H-NMR (500 MHz, mixture of CDCl_3_ and CD_3_OD); 2.90 (dd, 1H, *J_1_* = 14.0 Hz, *J_2_* = 8.0 Hz, C8), 2.84 (dd, 1H, *J_1_* = 14.0 Hz, *J_2_* = 8.0 Hz, C8), 2.07 (brs, 1H, C14), 1.87 (brs, 1H, C15), 1.83–1.78 (m, 1H, C9), 1.39–1.25 (m, 4H, CH_2_), 1.06–0.95 (m, 4H, CH_2_). ^13^C-NMR (125 MHz, mixture of CDCl_3_ and CD_3_OD); 199.1 (C7), 171.8 (C2), 163.1 (C4), 150.0 (C6), 95.8 (C3), 42.8 (C8), 41.5 (C15), 39.6 (C9), 38.1 (CH_2_), 37.2 (C14), 35.7 (CH_2_), 30.1 (CH_2_), 29.0 (CH_2_). MS (ES^−^); 263.12 (M−H), HRMS (M−H); calcd for C_13_H_15_N_2_O_4_; 263.1037; found; 263.1040.

#### 3.4.46. Synthesis of Compound *cis*-**28**


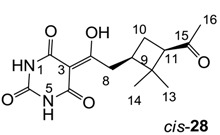


Yield; 61%; M.P.; 192 °C; ^1^H-NMR (500 MHz, CD_3_OD); 3.20 (dd, 1H, *J_1_* = 14.0 Hz, *J_2_* = 6.5 Hz, C11), 3.06–2.97 (m, 2H, C8), 2.45–2.40 (m, 1H, C9), 2.09–2.03 (m, 4H, C10 and C16), 1.91–1.86 (m, 1H, C10), 1.34 (s, 3H, C13), 0.92 (s, 3H, C14). ^13^C-NMR (125 MHz, CD_3_OD); 210.5 (C15), 199.3 (C7), 173.2 (C2), 164.3 (C4), 151.1 (C6), 96.2 (C3), 55.4 (C11), 45.1 (C12), 40.4 (C9), 37.7 (C8), 30.4 (C16), 30.3 (C13), 24.4 (C10), 18.0 (C14). MS (ES^−^); 293.12 (M−H); MS (ES^+^); 317.13 (M+Na); HRMS (M+Na); calcd for C_14_H_18_N_2_Na_1_O_5_; 317.1108; found; 317.1099.

### 3.5. Synthesis of Compound 5a Via O-Acylation Followed by Acyl Migration

#### *3.5.1. Synthesis of Compound* ***41***

To the mixture of barbituric acid **40d** (1.0 g, 6.40 mmol) and triethylamine (0.78 g, 7.70 mmol) in dichloromethane (50 mL) was slowly added 3,5,5-trimethylhexanoyl chloride (1.20 g, 6.72 mmol) at 0 °C under nitrogen atmosphere and the mixture was stirred for 3 h at room temperature. After completion of the reaction, the mixture was washed with 2 M HCl. The organic layer was dried over MgSO_4_ and evaporated *in vacuo* to give crude product. Further purification was carried out by recrystallization in the mixture of ethyl acetates and petrol giving pure compound **41** (1.56 g, 5.37 mmol, 84% yield) as a solid (M.P.; 49 °C)


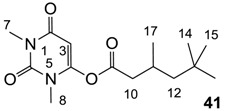


^1^H-NMR (400 MHz, CDCl_3_); 5.56 (s, 1H, C3), 3.27 (s, 3H, C8), 3.25 (s, 3H, C7), 2.57 (dd, 1H, *J_1_* = 15.6, *J_2_* = 6.0, C10), 2.37 (dd, 1H, *J_1_* = 15.6, *J_2_* = 8.0, C10), 2.14–2.01 (m, 1H, C11), 1.02 (d, 3H, *J* = 6.4 Hz, C12), 0.88–0.85 (m, 12H, C14–C17). ^13^C-NMR (100 MHz, CDCl_3_); 167.5 (C9), 162.3 (C2), 153.4 (C4), 151.1 (C6), 91.3 (C3), 50.2 (C12), 43.2 (C10), 30.9 (C13), 29.7 (C14-C16), 29.5 (C7), 28.0 (C8), 26.7 (C11), 22.3 (C17). MS (ES^−^); 295.18 (M−H), MS (ES^+^); 297.21 (M+H), 319.19 (M+Na), HRMS (M+Na); calcd for C_15_H_24_N_2_Na_1_O_4_; 319.1628; found; 319.1621.

#### 3.5.2. Synthesis of Compound (±)-**5a**

To the solution of compound 41 (500 mg, 1.69 mmol) in dichloromethane (30 mL) was added DMAP (250 mg, 2.02 mmol) at room temperature and the mixture was stirred overnight. After completion of the reaction, the mixture was washed with 2 M HCl and the organic layer was evaporated *in vacuo.* Short flash column chromatography of the crude material gave 3-acylbarbituric acid **5a** (245 mg, 0.845 mmol, 49 %) as oil.


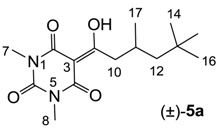


^1^H-NMR (400 MHz, CDCl_3_); 3.33 (s, 3H, C7), 3.29 (s, 3H, C8), 3.05 (d, 2H, *J* = 7.6 Hz, C10), 2.19–2.11 (m, 1H, C11), 1.32 (dd, 1H, *J_1_* = 14.0 Hz, *J_2_* = 3.6 Hz, C12), 1.15 (dd, 1H, *J_1_* = 14.0 Hz, *J_2_* = 6.8 Hz, C12), 0.98 (d, 3H, *J* = 6.4 Hz, C17), 0.87 (s, 9H, C14-C16). ^13^C-NMR (100 MHz, CDCl_3_); 199.1 (C9), 169.7 (C2), 160.7 (C4), 150.3 (C6), 95.8 (C3), 50.6 (C12), 45.2 (C10), 31.0 (C13), 29.8 (C14-C16), 27.9 (N-CH_3_), 27.8 (N-CH_3_), 27.7 (C11), 22.7 (C17). MS (ES^−^); 295.18 (M−H), HRMS (M−H); calcd for C_15_H_23_N_2_O_4_; 295.1663; found; 295.1678.

### 3.6. Synthesis of 3-Carboxamide an 3-Enamine Barbituric Acids

General procedure: To the solution of 3-alkoxycarbonyl or 3-acylbarbituric acid (1.0 equivalent) in toluene (or methanol for compound **36**) was added primary amine (1.0 equivalent) and the mixture was refluxed for 4–24 h checking TLC. After completion of the reaction, the solution was evaporated and column chromatography (or precipitation in methanol for compound **36**) gave metal-chelated 3-carboxamide tetramic acid or pure 3-enamine tetramic acid. In case of 3-carboxamide tetramic acid, the compound was dissolved in dichloromethane (50 mL) and washed with 1 N HCl (20 mL) to make metal free form. The organic layer was dried with MgSO_4_ and concentrated *in vacuo* to give metal free 3-carboxamide tetramic acid.

#### 3.6.1. Synthesis of Compound **29a**


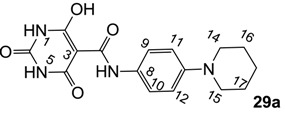


Yield; 63%; M.P.: >250 °C; ^1^H-NMR (400 MHz, DMSO); 11.54 (brs, 2H, NH), 11.37 (s, 1H, NH), 7.32 (d, 2H, *J* = 8.4 Hz, C9 and C10), 6.93 (d, 2H, *J* = 8.4 Hz, C11 and C12), 3.11 (brs, 4H, C14 and C15), 1.60 (brs, 4H, C16 and C17), 1.53 (brs, 2H, C18). ^13^C-NMR; not determined because of peak broading and solubility problems. MS (ES^−^); 329.13 (M−H); MS (ES^+^); 331.15 (M+H), 353.13 (M+Na); HRMS (M+H); calcd for C_16_H_19_N_4_O_4_; 331.1401; found; 331.1392.

#### 3.6.2. Synthesis of Compound **29b**


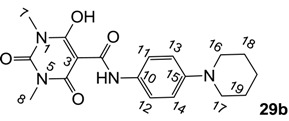


Yield; 77%; M.P.; 180 °C; ^1^H-NMR (500 MHz, CD_3_OD); 7.78 (d, 2H, *J* = 9.5 Hz, C11 and C12), 7.68 (d, 2H, *J* = 9.5 Hz, C13 and C14), 3.62 (t, 4H, *J* = 6.0 Hz, C16 and C17), 3.34 (s, 6H, C7 and C8), 2.07–2.03 (m, 4H, C18 and C19), 1.81 (brs, 2H, C20). ^13^C-NMR; not determined because of peak broading and solubility problems. MS (ES^−^); 357.16 (M−H); HRMS (M−H); calcd for C_18_H_21_N_4_O_4_; 357.1568; found; 357.1568.

#### 3.6.3. Synthesis of Compound **29c**


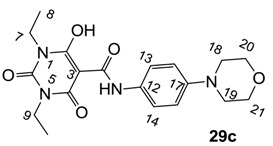


Yield; 48%; M.P.: 167 °C; ^1^H-NMR (400 MHz, CDCl_3_); 11.82 (brs, 1H, NH or OH), 7.41 (d, 2H, *J* = 8.8 Hz, C13 and C14), 6.91 (d, 2H, *J* = 8.8 Hz, C15 and C16), 4.02 (q, 4H, *J* = 6.8 Hz, C7 and C9), 3.86 (t, 4H, *J* = 4.8 Hz, C20 and C21), 3.16 (t, 4H, *J* = 4.8 Hz, C18 and C19), 1.29–1.24 (m, 6H, C8 and C9). ^13^C-NMR (100 MHz, CDCl_3_); 168.9 (C11), 168.4 (C4), 163.0 (C2), 149.4 (C6), 149.2 (C17), 127.8 (C12), 122.9 (C13 and C14), 115.9 (C15 and C16), 80.5 (C3), 66.7 (C20 and C21), 49.1 (C18 and C19), 36.6 (N-CH_2_), 36.3 (N-CH_2_), 13.3 (CH_3_), 13.2 (CH_3_). MS (ES^−^); 387.17 (M−H); HRMS (M+Na); calcd for C_19_H_24_N_4_Na_1_O_5_; 411.1639; found; 411.1627.

#### 3.6.4. Synthesis of Compound **30**


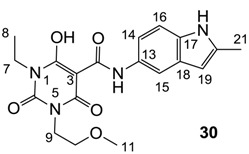


Yield; 53%; M.P. 160 °C; ^1^H-NMR (400 MHz, DMSO); 11.79 (s, 1H, NH or OH), 11.04 (s, 1H, indole NH), 7.55 (s, 1H, C15), 7.27 (d, 1H, *J* = 8.8 Hz, C14), 7.04 (dd, 1H, *J_1_* = 8.8 Hz, *J_2_* = 2.0 Hz, C16), 6.12 (s, 1H, C19), 3.99 (t, 2H, *J* = 6.0 Hz, C9), 3.85 (q, 2H, *J* = 6.8 Hz, C7), 3.50 (t, 2H, *J* = 6.0 Hz, C10), 3.25 (s, 3H, C11), 2.37 (s, 3H, C21), 1.13 (t, 3H, *J* = 6.8 Hz, C8). ^13^C-NMR (100 MHz, DMSO); 168.3 (C2, C4 and C12), 149.1 (C6), 137.3 (Ar quart-C), 134.2 (Ar quart-C), 128.7 (Ar quart-C), 126.7 (Ar quart-C), 115.1 (Ar tert-C), 112.5 (Ar tert-C), 110.8 (Ar tert-C), 99.5 (C19), 79.7 (C3), 68.4 (C10), 58.0 (C11), 39.5 (C9, overlapping with DMSO peak, confirmed in DEPT NMR), 35.9 (C7), 13.4 (CH_3_), 13.0 (CH_3_). MS (ES^−^); 385.16 (M−H); HRMS (M+Na); calcd for C_19_H_22_N_4_Na_1_O_5_; 409.1482; found; 409.1474.

#### 3.6.5. Synthesis of Compound **31a**


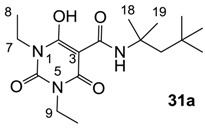


Yield; 67%; M.P.: 92 °C; ^1^H-NMR (400 MHz, CDCl_3_); 10.16 (brs, 1H, NH or OH), 3.97–3.90 (m, 4H, C7 and C9), 1.78 (s, 2H, C13), 1.37 (s, 6H, C18 and C19), 1.22–1.16 (m, 6H, C8 and C10), 0.98 (s, 9H, C15-C17). ^13^C-NMR (100 MHz, CDCl_3_); 170.4 (C11), 168.2 (C4), 163.1 (C2), 149.6 (C6), 80.1 (C3), 56.6 (C12), 51.7 (C13), 36.2 (N-CH_2_), 36.0 (N-CH_2_), 31.6 (C14), 31.2 (C15-C17), 29.5 (C18 and C19), 13.3 (CH_3_), 13.2 (CH_3_). MS (ES^−^); 338.22 (M−H); MS (ES^+^); 340.21 (M+H), 362.21 (M+Na); HRMS (M+Na); calcd for C_17_H_29_N_3_Na_1_O_4_; 362.2050; found; 362.2037.

#### 3.6.6. Synthesis of Compound **31b**


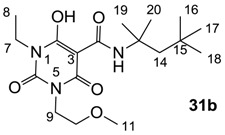


Yield; 68 % (oil); ^1^H-NMR (400 MHz, CDCl_3_); 10.21 and 10.14 (2 of s, 1H, NH or OH), 4.15–4.11 (m, 2H, C9), 3.98–3.92 (m, 2H, C7), 3.63–3.57 (m, 2H, C10), 3.35 and 3.34 (2 of s, 3H, C11), 1.79 (s, 2H, C14), 1.49 (s, 6H, C19 and C20), 1.24–1.18 (m, 3H, C8), 0.99 (s, 9H, C16-C18). ^13^C-NMR (100 MHz, CDCl_3_); 170.4 and 170.3 (C12), 168.4 and 168.2 (C4), 163.2 and 163.1 (C2), 149.9 and 149.8 (C6), 80.0 (C3), 69.5 and 69.4 (C10), 58.7 (C11), 56.7 and 56.6 (C13), 51.7 and 51.6 (C14), 39.9 and 39.8 (C9), 36.3 and 36.1 (C7), 31.6 (C15), 31.2 (C16-C18), 29.4 (C19 and C20), 13.3 and 13.2 (C8). MS (ES^−^); 368.23 (M−H); MS (ES^+^); 370.24 (M+H), 392.21 (M+Na); HRMS (M+Na); calcd for C_18_H_31_N_3_ Na_1_O_5_; 392.2156; found; 392.2141.

#### 3.6.7. Synthesis of Compound **32**


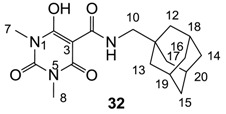


Yield; 74%; M.P.: 132 °C; ^1^H-NMR (500 MHz, CDCl_3_); 10.09 (brs, 1H, NH or OH), 3.33–3.32 (m, 6H, C7 and C8), 3.09 (d, 2H, *J* = 6.5 Hz, C10), 2.00 (brs, 3H, C18-C20), 1.73–1.62 (m, 6H, CH_2_), 1.53 (brs, 6H, CH_2_). ^13^C-NMR (125 MHz, CDCl_3_); 170.8 (C9), 168.2 (C4), 163.4 (C2), 150.6 (C6), 79.7 (C3), 51.6 (C10), 40.0 (C12, C13 and C17), 36.6 (C14–C16), 33.6 (C11), 28.0 (C18–C20), 27.5 (CH_3_), 27.4 (CH_3_). MS (ES^−^); 346.17 (M−H); HRMS (M−H); calcd for C_18_H_24_N_3_O_4_; 346.1772; found; 346.1772.

#### 3.6.8. Synthesis of Compound **33a**


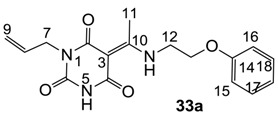


Yield; 96%; M.P.; 142 °C; Mixture of two exo-enol tautomers (E1: E2 = 45: 55); ^1^H-NMR (400 MHz, CDCl_3_); 12.77 (brs, 1H, NH E2), 12.71 (brs, 1H, NH E1), 8.68 (brs, 1H, NH), 7.33–7.27 (m, 2H, C17 and C18), 7.02–6.97 (m, 1H, C19), 6.92 (d, 2H, *J* = 8.8 Hz, C15 and C16), 5.94–5.84 (m, 1H, C8), 5.22 (d, 1H, *J* = 17.2 Hz, C9), 5.16 (d, 1H, *J* = 10.4 Hz, C9), 4.50 (d, 2H, J = 5.2 Hz, C7), 4.20 (t, 2H, *J* = 5.2 Hz, C13), 3.89–3.85 (m, 2H, C12), 2.79 (s, 3H, C11 E2), 2.77 (s, 3H, C11 E1). ^13^C-NMR (100 MHz, CDCl_3_); 175.2 (C10 E1), 174.7 (C10 E2), 167.0 (C2 E2), 166.3 (C4 E1), 163.2 (C2 E1), 163.1 (C4 E2), 157.9 (C14), 149.8 (C6), 132.6 (C8 E1), 132.4 (C8 E2), 129.6 (C17 and C18), 121.7 (C19), 116.9 (C9 E2), 116.7 (C9 E1), 114.6 (C15 and C16), 90.3 (C3), 65.7 (C13), 43.2 (C12 E1), 43.1 (C12 E2), 42.4 (C7 E1), 42.2 (C7 E2), 17.8 (C11). MS (ES^−^); 328.15 (M−H); MS (ES^+^); 352.15 (M+Na); HRMS (M+Na); calcd for C_17_H_19_N_3_Na_1_O_4_; 352.1268; found; 352.1254.

#### 3.6.9. Synthesis of Compound **33b**


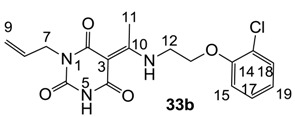


Yield; 84%; M.P.; 173 °C; Mixture of two exo-enol tautomers (E1: E2 = 45: 55); ^1^H-NMR (500 MHz, CDCl_3_); 12.82 (brs, 1H, NH E2), 12.73 (brs, 1H, NH E1), 8.34 (brs, 1H, NH E1), 8.25 (brs, 1H, NH E2), 7.39–7.37 (m, 1H, C18), 7.24–7.20 (m, 1H, C17), 6.98–6.92 (m, 2H, C15 and C19), 5.93–5.85 (m, 1H, C8), 5.24–5.14 (m, 2H, C9), 4.50 (d, 2H, *J* = 5.5 Hz, C7), 4.24 (t, 2H, *J* = 5.0 Hz, C13), 3.97–3.93 (m, 2H, C12), 2.85 (s, 3H, C11 E2), 2.83 (s, 3H, C11 E1). ^13^C-NMR (125 MHz, CDCl_3_); 175.7 (C10 E1), 175.1 (C10 E2), 167.1 (C2 E2), 166.2 (C4 E1), 163.2 (C2 E1), 162.8 (C4 E2), 153.6 (C14), 149.8 (C6 E1), 149.7 (C6 E2), 132.6 (C8 E1), 132.3 (C8 E2), 130.6 (C18), 127.8 (C17 E1), 127.6 (C17 E2), 123.4 (C16 E2), 123.3 (C16 E1), 122.6 (C19 E2), 122.6 (C19 E1), 117.0 (C9 E2), 116.8 (C9 E1), 113.9 (C15 E2), 113.9 (C15 E1), 90.4 (C3), 67.4 (C13 E1), 67.3 (C13 E2), 43.1 (C12 E1), 43.0 (C12 E2), 42.4 (C7 E1), 42.2 (C7 E2), 17.8 (C11 E1), 17.8 (C11 E2). MS (ES^−^); 362.11 (M−H); MS (ES^+^); 386.11 (M+Na); HRMS (M+Na); calcd for C_17_H_18_Cl_1_N_3_Na_1_O_4_; 386.0878; found; 386.0876.

#### 3.6.10. Synthesis of Compound **34**


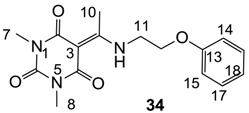


Yield; 63%; M.P.; 129 °C; ^1^H-NMR (400 MHz, CDCl_3_); 12.90 (brs, 1H, NH), 7.29 (dd, 2H, *J_1_* = *J_2_* = 7.6 Hz, C16 and C17), 6.99 (t, 1H, *J* = 7.6 Hz, C18), 6.92 (d, 2H, *J* = 7.6 Hz, C14 and C15), 4.19 (t, 2H, *J* = 5.2 Hz, C12), 3.87–3.83 (m, 2H, C11), 3.30 (s, 6H, C7 and C8), 2.76 (s, 3H, C10). ^13^C-NMR (100 MHz, CDCl_3_); 174.5 (C9), 166.3 (C4), 162.9 (C2), 157.9 (C13), 151.3 (C6), 129.5 (C16 and C17), 121.6 (C18), 114.6 (C14 and C15), 90.7 (C3), 65.7 (C12), 43.1 (C11), 27.8 (NCH_3_), 27.5 (NCH_3_), 17.9 (C10). MS (ES^−^); 316.15 (M−H); MS (ES^+^); 318.17 (M+H), 340.15 (M+Na); HRMS (M+Na); calcd for C_16_H_19_N_3_Na_1_O_4_; 340.1268; found; 340.1256.

#### 3.6.11. Synthesis of Compound **35**


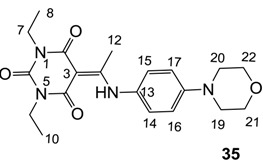


Yield; 66%; M.P.; 209 °C; ^1^H-NMR (400 MHz, CDCl_3_); 7.03 (d, 2H, *J* = 8.8 Hz, C16 and C17), 6.90 (d, 2H, *J* = 8.8 Hz, C14 and C15), 4.01–3.93 (m, 4H, C7 and C9), 3.84–3.82 (m, 4H, C21 and C22), 3.17–3.15 (m, 4H, C19 and C20), 2.59 (s, 3H, C12), 1.23–1.17 (m, 6H, C8 and C10). ^13^C-NMR (100 MHz, CDCl_3_); 173.3 (C11), 166.0 (C4), 162.5 (C2), 150.5 (C18), 150.4 (C6), 127.9 (C13), 126.5 (C14 and C15), 115.6 (C16 and C17), 91.3 (C3), 66.6 (C21 and C22), 48.6 (C19 and C20), 36.1 (N-CH_2_), 36.0 (N-CH_2_), 20.1 (C12), 13.35 (CH_3_), 13.33 (CH_3_). MS (ES^-^); 385.19 (M−H); HRMS (M−H); calcd for C_20_H_25_N_4_O_4_; 385.1881; found; 385.1888.

#### 3.6.12. Synthesis of Compound **36**


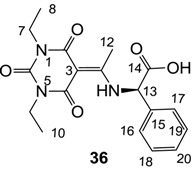


Yield; 60%; M.P.; 203 °C; ^1^H-NMR (400 MHz, CDCl_3_); 13.55 (d, 1H, *J* = 6.4 Hz, NH), 9.04 (brs, 1H, OH), 7.41–7.38 (m, 5H, C16-C20), 5.44 (d, 1H, *J* = 6.4 Hz, C13), 4.01 (q, 2H, *J* = 6.8 Hz, NCH_2_), 3.93 (q, 2H, *J* = 6.8 Hz, NCH_2_), 2.55 (s, 3H, C12), 1.21 (t, 3H, *J* = 6.8 Hz, CH_3_), 1.14 (t, 3H, *J* = 6.8 Hz, CH_3_). ^13^C-NMR (100 MHz, CDCl_3_); 173.8 (C11), 171.8 (C14), 165.9 (C4), 162.8 (C2), 150.5 (C6), 134.9 (C15), 129.5 (C16 and C17), 129.3 (C20), 127.0 (C18 and C19), 92.0 (C3), 60.5 (C13), 36.4 (N-CH_2_), 36.3 (N-CH_2_), 19.1 (C12), 13.4 (CH_3_), 13.3 (CH_3_).

#### 3.6.13. Synthesis of Compound (±)-**37**


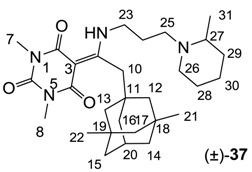


Yield; 82% (oil); ^1^H-NMR (400 MHz, CDCl_3_); 4.54–4.51 (m, 1H, NH), 3.65 (brs, 1H, C23), 3.43 (brs, 1H, C23), 3.29 (s, 3H, C7), 3.27 (s, 3H, C8), 2.75–2.68 (m, 2H, C10), 2.27 (brs, 3H, C26 and C27), 2.07–1.97 (m, 2H, C25), 1.77–1.75 (m, 2H, CH_2_), 1.61–0.99 (m, 21H, C12-C17, C20 and C24, 6H of CH_2_), 0.74 (s, 6H, C21 and C22). ^13^C-NMR (100 MHz, CDCl_3_); 175.0 (C9), 166.3 (C2), 163.2 (C4), 151.2 (C6), 91.2 (C3), 55.9 (C27), 51.6 (CH_2_), 50.6 (CH_2_), 50.4 (CH_2_), 50.1 (CH_2_), 48.9 (CH_2_), 43.1 (CH_2_), 42.7 (CH_2_), 40.6 (CH_2_), 39.4 (CH_2_), 38.7 (C18 and C19), 34.4 (CH_2_), 31.5 (C11), 30.5 (C21 and C22), 29.7 (C20), 28.0 (N-CH_3_), 27.6 (N-CH_3_), 26.6 (CH_2_), 26.1 (CH_2_), 23.4 (CH_2_), 18.4 (C31). MS (ES^−^); 497.38 (M−H); MS (ES^+^); 499.36 (M+H); HRMS (M+H); calcd for C_29_H_47_N_4_O_3_; 499.3643; found; 499.3629.

## 4. Conclusions

In this paper, we report the synthesis, tautomerism and antibacterial activity with SAR study of novel barbiturates inspired from antibacterial tetramates. Efficient synthetic methodologies leading to 3-acyl, 3-carboxamide and 3-enamine barbiturates permitted a wide range of substituent diversity on the core heterocycle. Computational studies of the favoured tautomeric forms reveals that all of 3-acyl, 3-alkoxycarbonyl, 3-carboxamido and 3-enamine barbituric acids in solution are favored to be the enol-tautomeric form rather than the keto-form, in which the *exo*-enol form for 3-acyl, 3-carboxamide and 3-enamine and *endo*-enol form for 3-alkoxycarbonyl barbiturate are more stable than the alternative enol-form. It was found that SAR of substituted barbiturates is similar to tetramates. Thus, 3-acyl and 3-carboxamide barbiturates depending on their substituents (N-disubstitution preferred), along with the equivalent tetramates, exhibited antibacterial activity, especially against resistant and susceptible Gram-positive strains, such as MRSA, VSE, VRE and MDRSP. Of particular interest is that the active barbiturates possess amenable molecular weight, rotatable bonds and the number of proton-donors/acceptors for drug design as well as less lipophilic character, and which are similar to antibiotic agents for oral and injectable use. In addition, it was found that not only lipophilicity and MSA, as discussed in our previous papers [13,14,15,19], but also PSA affected the efficiency of transportation by efflux pump in *H. influenzae.* Barbiturates tended to be more easily transported than tetramates because of their higher PSA. As expected, replacement of the tetramic acid by barbituric acid gave a positive effect for PPB affinity. Unfortunately, the reduction of PPB affinity by the barbituric core is not sufficient to achieve activity *in vivo*, although the physicochemical properties and ionic state are similar to antibiotic agents for oral and injectable use. Further optimization to reduce PPB affinity or elevate antibiotic potency is therefore required. On the other hand, physicochemical property-activity relationship analysis of clinical antibiotics shows that oral and injectable antibiotics may have a higher margin for lipophilicity depending on molecular size (MSA) in order to achieve low PPB affinity, while topical antibiotics are free from such constraints. In addition, Gram-negative active antibiotics tended to exist as more ionized microspecies than Gram-positive antibiotics to penetrate outer membrane. We believe that these systems offer unusual opportunities for antibiotic drug discovery, and these results suggest that a strategy based upon the use of natural products is viable [41,42], and highlights the importance of continuing development of methodologies to access tetramate-like systems [43]. The work contained here-in, and another recently reported study [44], indicate that barbiturates offer a core template of promise and worthy of further investigation.

## Supplementary Materials

Supplementary materials can be accessed at: http://www.mdpi.com/1420-3049/20/03/3582/s1.
