# Supplementary material for: Antibacterial Barbituric Acid Analogues Inspired from Natural 3-Acyltetramic Acids; Synthesis, Tautomerism and Structure and Physicochemical Property-Antibacterial Activity Relationships
Source: Molecules. 2015 Feb 20;20(3):3582–627. doi: 10.3390/molecules20033582 (PMC6272196; doi:10.3390/molecules20033582)
Supplement: Supplementary file 1 [file molecules-20-03582-s001.pdf]

Supplementary

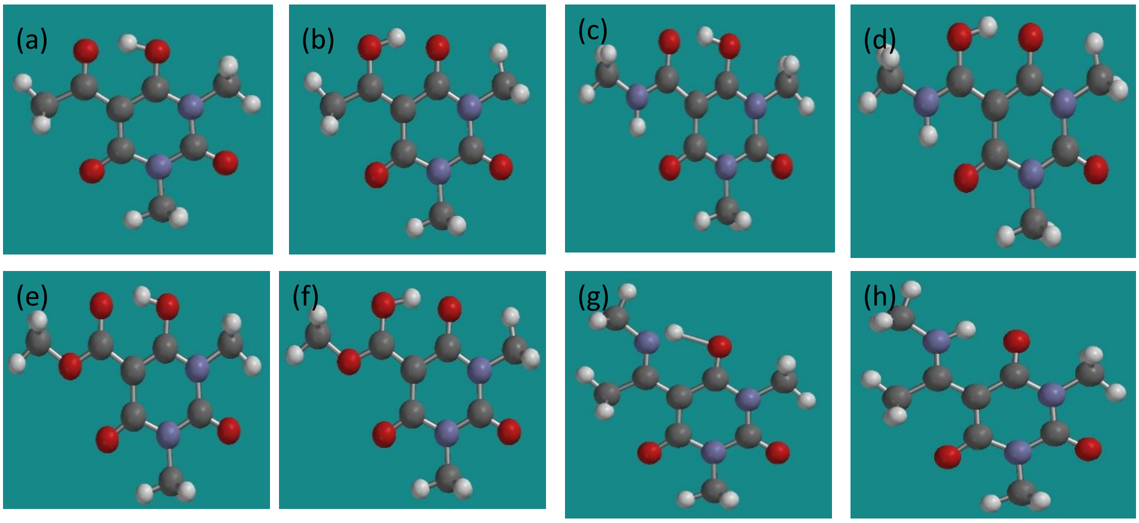

Figure S1. Most stable geometry at ground state in computational calculation; 3-acyl **2a** (a) *endo*-enol, (b) *exo*-enol, 3-carboxamide **43a** (c) *endo*-enol, (d) *exo*-enol, 3-alkoxycarbonyl **43b** (e) *endo*-enol, (f) *exo*-enol, 3-enamine **43c** (g) *endo*-enol and (h) *exo*-enol.

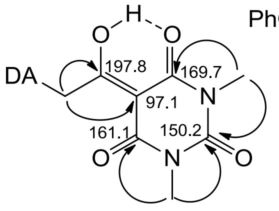

6a *exo*-enol

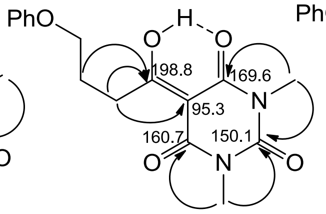

17a *exo*-enol

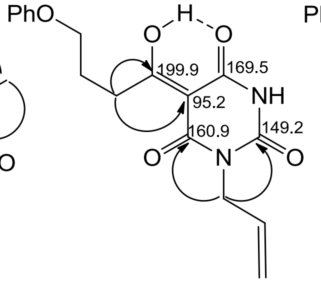

26a *exo*-enol 1

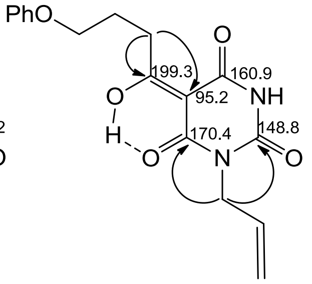

26a *exo*-enol 2

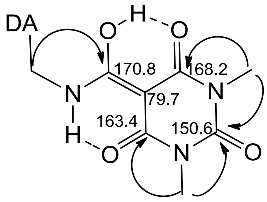

32 *exo*-enol

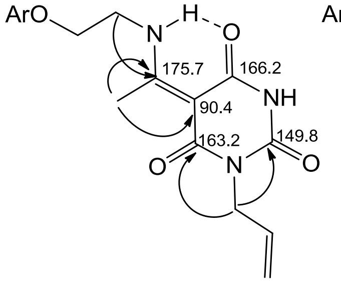

33b *exo*-enol 1

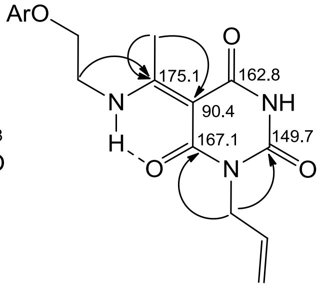

33b *exo*-enol 2

Figure S2. HMBC correlation of representative analogues.

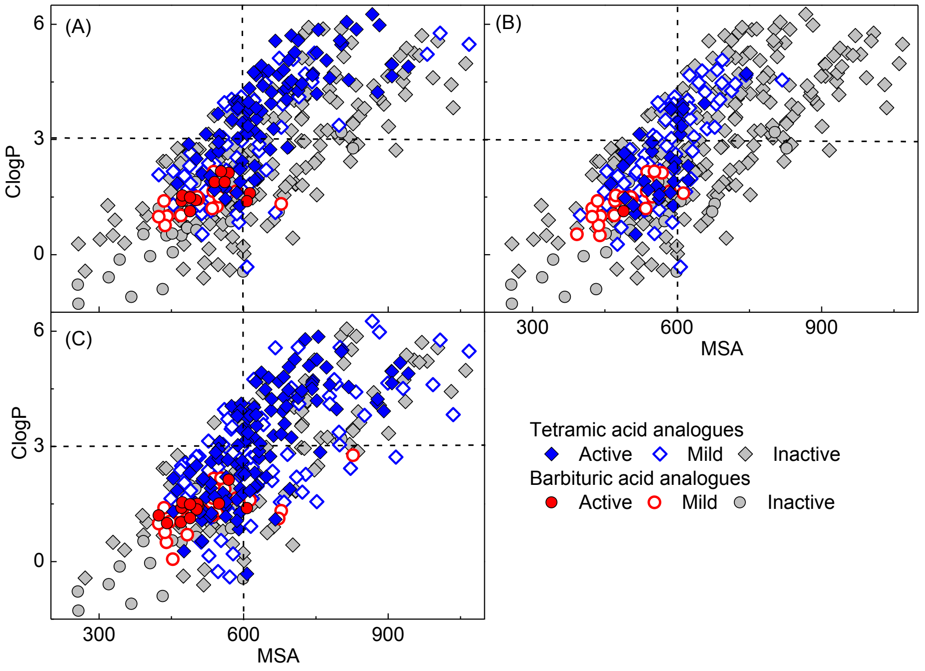

Figure S3. Plot of ClogP against MSA of barbituric acids 2-37 along with tetramic acids in our previous reports [1–4] against (A) MRSA, (B) *H. influenzae 3* and (C) efflux-negative *H. influenzae 4*. Active, mild and inactive mean that the values are MIC ≤ 4 µg/mL, 4 µg/mL < MIC ≤ 32 µg/mL and MIC > 32 µg/mL, respectively.

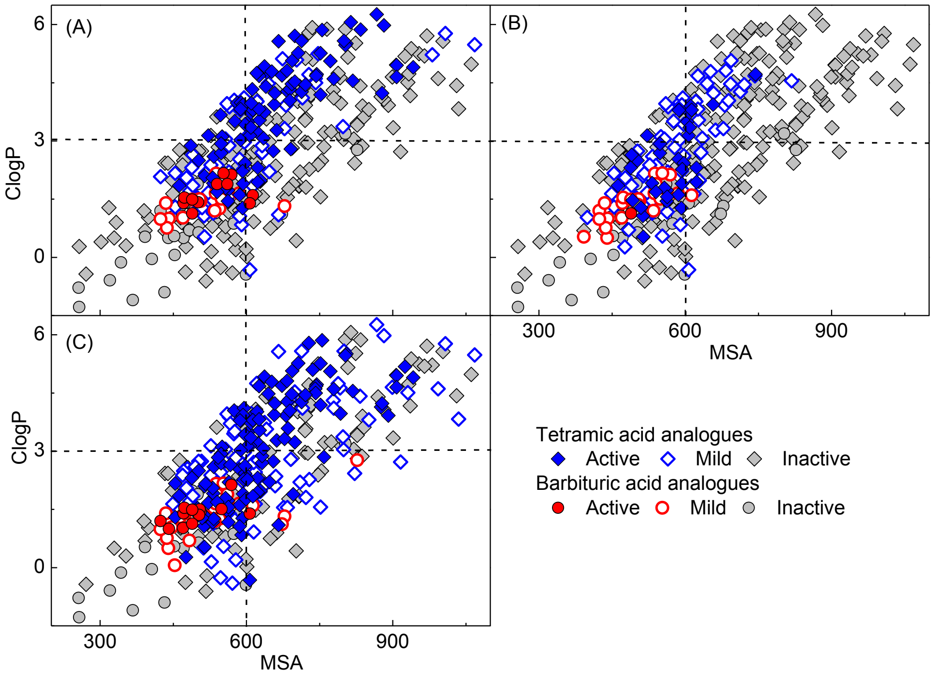

Figure S4. Plot of PSA against MSA of barbituric acids 2-37 along with tetramic acids in our previous reports [1–4] against (A) MRSA, (B) *H. influenzae 3* and (C) efflux-negative *H. influenzae 4*. Active, mild and inactive mean that the values are MIC ≤ 4 µg/mL, 4 µg/mL < MIC ≤ 32 µg/mL and MIC > 32 µg/mL, respectively.

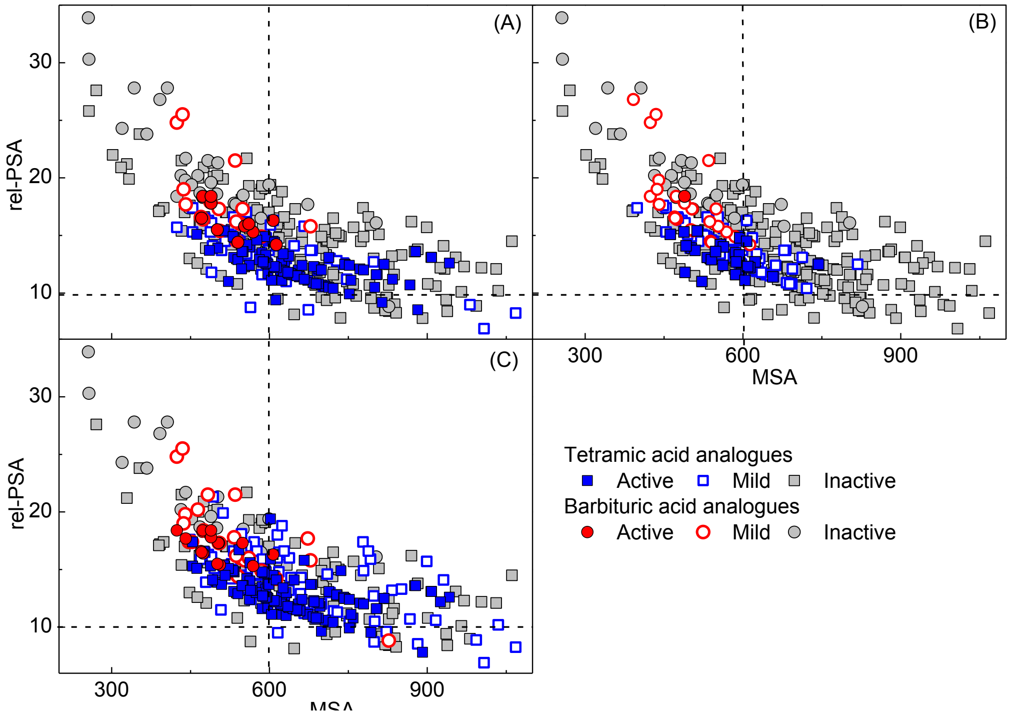

Figure S5. Plot of rel-PSA against MSA of barbituric acids **2–37** along with tetramic acids in our previous reports [1–4] against (**A**) MRSA; (**B**) *H. influenzae 3* and (**C**) efflux-negative *H. influenzae 4*. Active, mild and inactive mean that the values are MIC ≤ 4 µg/mL, 4 µg/mL < MIC ≤ 32 µg/mL and MIC > 32 µg/mL, respectively.

Table S1. Physicochemical properties of barbiturates 2–37 [a,b].

	Mw	MSA	PSA	re-PSA	ClogP	ClogD$_{7.4}$	H-D/H-A	RB
**2a**	198	257	77.9	30.3	−1.28	−2.26	1/4	0
**2b**	226	320	77.9	24.3	−0.59	−1.44	1/4	2
**2c**	256	367	87.2	23.8	−1.10	−1.99	1/5	4
**3**	284	432	87.2	20.2	−0.90	−2.26	1/5	4
**4**	282	447	77.9	17.4	1.04	0.11	1/4	4
**5a**	296	474	77.9	16.4	1.48	0.62	1/4	4
**5b**	324	538	77.9	14.5	2.16	1.36	1/4	6
**5c**	354	585	87.2	14.9	1.65	0.81	1/5	8
**6a**	332	470	77.9	16.6	1.02	0.00	1/4	2
**6b**	346	506	77.9	15.4	1.45	0.46	1/4	2
**6c**	360	540	77.9	14.4	1.89	0.93	1/4	2
**7a**	346	501	77.9	15.5	1.42	0.51	1/4	3
**7b**	404	613	87.2	14.2	1.60	0.70	1/5	7
**8a**	294	441	77.9	17.7	1.00	0.10	1/4	3
**8b**	308	471	77.9	16.5	1.40	0.54	1/4	4
**9**	352	545	87.2	16.0	1.26	−1.35	1/5	4
**10**	350	533	95.0	17.8	1.54	0.61	1/5	4
**11**	350	536	95.0	17.7	1.14	0.15	1/5	5
**12**	392	584	96.4	16.5	1.05	−0.06	1/6	9
**13**	541	803	129	16.1	3.19	2.10	1/7	4
**14**	523	-[b]	141	-[b]	1.71	0.17	1/9	5

Table S1. *Cont.*

	Mw	MSA	PSA	re-PSA	ClogP	$ClogD_{7.4}$	H-D/H-A	RB
**15a**	407	599	116	19.4	−0.44	−3.42	2/6	6
**15b**	434	678	107	15.8	1.32	0.15	2/5	6
**16**	476	673	119	17.7	1.12	−0.34	2/7	6
**17a**	318	440	87.2	19.8	0.50	−0.65	1/5	5
**17b**	332	471	87.2	18.5	0.50	−0.59	1/5	6
**17c**	368	504	87.2	17.3	1.50	0.37	1/5	5
**18a**	346	503	87.2	17.3	1.36	0.15	1/5	6
**18b**	374	569	87.2	15.3	2.13	0.91	1/5	6
**18c**	346	504	87.2	17.3	1.44	0.41	1/5	5
**18d**	346	505	87.2	17.3	1.44	0.40	1/5	5
**18e**	367	488	87.2	17.9	1.49	0.03	1/5	5
**18f**	387	473	87.2	18.4	1.54	−0.32	1/5	5
**18g**	367	489	87.2	17.8	1.49	0.03	1/5	5
**19a**	395	553	87.2	15.8	2.17	0.80	1/5	7
**19b**	360	537	87.2	16.2	1.65	0.53	1/5	7
**20**	372	549	95.0	17.3	1.51	0.05	1/5	5
**21**	390	483	104	21.5	0.70	−0.82	1/5	4
**22**	302	424	77.9	18.4	1.20	0.27	1/4	4
**23a**	327	437	82.9	19.0	0.76	−0.63	1/4	2
**23b**	341	464	93.7	20.2	1.30	0.42	2/4	4
**24**	332	475	87.2	18.4	1.40	−1.46	1/5	3
**25a**	210	256	86.7	33.9	−0.78	−1.80	2/4	2
**25b**	308	473	86.7	18.3	1.97	1.05	2/4	6
**26a**	330	441	95.9	21.7	1.00	−0.21	2/5	7
**26b**	379	489	95.9	19.6	1.98	0.48	2/5	7
**26c**	325	392	105	26.8	0.53	−1.24	3/5	5
**26d**	339	424	105	24.8	0.99	−0.63	3/5	5
**27**	264	343	95.5	27.8	−0.13	−1.34	3/4	2
**28**	294	406	113	27.8	−0.04	−1.28	3/5	3
**29a**	330	438	111	25.3	1.40	0.83	4/6	3
**29b**	358	502	93.2	18.6	1.89	1.51	2/6	3
**29c**	388	552	102	18.5	1.51	1.23	2/7	5
**30**	386	536	115	21.5	1.20	0.93	3/6	6
**31a**	339	562	90.0	16.0	1.89	2.14	2/5	6
**31b**	369	609	99.2	16.3	1.39	1.62	2/6	8
**32**	347	491	90.0	18.3	1.13	1.34	2/5	3
**33a**	329	452	87.7	19.4	0.55	0.46	2/5	7
**33b**	364	468	87.7	18.7	1.07	0.92	2/5	7
**34**	317	453	79.0	17.4	0.06	0.06	1/5	5
**35**	386	574	82.2	14.3	0.87	0.87	1/6	5
**36**	359	502	107	21.3	0.64	−2.60	2/6	6
**37**	499	827	73.0	8.83	2.77	0.10	1/5	7

a; **Mw**; molecular weight, **MSA**; molecular surface area, **PSA**; polar surface area, **%PAS**; relative polar surface area = (PSA/MSA) × 100, **ClogP**; calculated partition coefficient, **$ClogD_{7.4}$**; calculated distribution coefficient at pH 7.4, **HD**; hydrogen bond donor count, **HA**; hydrogen bond acceptor count, **RB**; rotatable bond count, b; could not be calculated.

Table S2. Physical properties of topical antibiotics [a,b].

Entry	Name	Mw	MSA	PSA	%PSA	ClogP	ClogD$_{7.4}$	HD/HA	RB
1	Fusidic acid	517	844	104	12.3	4.26	1.57	3/5	6
2	Clofoctol	365	569	20.2	3.6	8.06	8.06	1/1	5
3	Triclosan	290	319	29.5	9.24	4.75	4.56	1/1	2
4	Mupirocin	501	812	146	18.0	2.12	−0.41	4/8	17
5	Retapamulin	518	838	66.8	12.5	4.59	2.31	1/4	6
6	REP8839	450	529	53.2	9.94	4.44	2.76	3/4	8
7	Torezolid	370	476	106	22.2	2.41	2.41	1/7	4
8	Azidamfenicol	295	397	145	36.5	−0.53	−0.54	3/7	7
9	Meclocycline	477	547	182	33.3	−1.27	−6.89	6/9	2

a; see foot note in Table S1 for the abbreviation, b; current antibiotics, see reference [5–8];

Table S3. Physical properties of Gram-positive only antibiotics.

Entry	Name	Mw	MSA	PSA	%PSA	ClogP	ClogD$_{7.4}$	HD/HA	RB
				Penicillins					
1	Cloxacillin	435	538	113	21.0	1.94	−1.43	2/5	4
2	Dicloxacillin	469	553	113	20.4	2.45	−0.91	2/5	3
3	Oxacillin	401	522	113	21.6	1.42	−1.95	2/5	4
4	Penicillin G	334	449	86.7	19.3	0.93	−2.53	2/4	4
5	Penicillin V	350	466	95.9	20.6	0.59	−2.93	2/5	5
6	Nafcillin	400	528	95.9	18.2	1.56	−1.97	2/5	4
7	Meticillin	380	515	105	20.4	0.31	−3.30	2/6	5
				Lincosamides, Oxazolidinones & 2,4-Diaminopyrimidines					
8	Lincomycin	407	630	122	19.4	−0.80	−1.47	5/7	7
9	Clindamycin	425	635	102	16.1	0.65	0.27	4/6	7
**10**	**Linezolid**	**337**	**482**	**71.1**	**14.8**	**0.76**	**0.76**	**1/5**	**4**
11	Ranbezolid	461	641	124	19.4	1.52	1.50	1/7	7
12	Radezolid	438	606	112	18.5	1.67	1.32	3/6	8
13	Iclaprim	354	502	106	21.0	2.34	2.16	2/7	5
14	Trimethoprim	344	481	115	23.8	1.73	1.55	2/8	5

Table S4. Physical properties of Gram positive and negative penicillins and other antibiotics.

Entry	Name	Mw	MSA	PSA	%PSA	ClogP	$ClogD_{7.4}$	HD/HA	RB
				Penicillins					
1	Carbenicillin	378	481	124	25.8	0.72	−6.20	3/6	5
2	Ticarcillin	384	460	124	27.0	0.39	−6.35	3/6	5
3	Ampicillin	349	466	113	24.2	0.15	−3.55	3/5	4
4	Cyclacillin	341	486	113	23.3	−0.25	−4.11	3/5	3
5	Amoxicillin	365	476	133	27.9	−0.13	−3.84	4/6	4
6	Azlocillin	461	595	148	24.9	−0.25	−3.74	4/6	5
7	Mezlocillin	540	690	174	25.2	−0.59	−4.07	3/8	5
8	Piperacillin	518	685	156	22.8	−0.33	−3.81	3/7	6
				Penems and carbapenems					
9	Faropenem	285	354	87.1	24.6	−1.82	−4.92	2/5	3
10	Ertapenem	476	608	156	25.7	−1.39	−7.73	5/8	7
11	Doripenem	421	551	162	29.4	−3.79	−6.88	5/8	6
12	Meropenem	383	531	110	20.7	−2.82	−5.91	3/6	5
13	Imipenem	313	417	114	27.3	−2.27	−5.30	4/6	6
14	Tomopenem	538	741	192	25.9	−3.63	−9.54	6/10	8
				Amphenicols					
15	Chloramphenicol	323	389	115	29.7	0.57	0.34	3/5	6
16	Thiamphenicol	356	442	104	23.5	−0.61	−0.79	3/5	6

Table S5. Physical properties of cephalosporins (1st and 2nd generations).

Entry	Name	Mw	MSA	PSA	%PSA	ClogP	$ClogD_{7.4}$	HD/HA	RB
1	Cefalexin (1st)	347	435	113	26.0	−0.31	−3.97	3/5	4
2	Cefadroxil (1st)	363	446	133	29.8	−0.60	−4.26	4/6	4
3	Cefaloglycin (1st)	405	509	139	27.3	−1.18	−4.87	3/6	7
4	Cefradine (1st)	349	428	113	26.4	−0.81	−4.51	3/5	4
5	Cefroxadine (1st)	365	444	122	27.5	−2.20	−5.93	3/6	5
6	Cefapirin (1st)	423	509	126	24.8	−1.48	−4.93	2/6	8
7	Cefalotin (1st)	396	471	113	24.0	−0.58	−4.01	2/5	7
8	Cefatrizine (1st)	463	534	175	32.8	−0.35	−4.08	5/8	7
9	Cefalonium (1st)	459	548	137	25.0	−4.68	−5.05	2/5	7
10	Cefaloridine (1st)	415	505	93.4	18.5	−3.33	−3.70	1/4	6
11	Ceftezole (1st)	440	466	156	33.5	−2.65	−6.27	2/9	7
12	Cefazolin (1st)	455	498	156	31.3	−2.36	−5.96	2/9	7
13	Cefazedone (1st)	548	555	133	24.0	0.96	−2.61	2/8	7
14	Cefazaflur (1st)	470	499	130	26.1	0.50	−3.05	2/7	8
15	Cefacetrile (1st)	339	399	137	34.3	−2.56	−6.94	2/6	6
16	Cerbacephem (2nd)	350	428	113	26.4	−0.96	−4.68	3/5	4
17	Cefaclor (2nd)	368	419	113	27.0	−0.83	−4.56	3/5	4
18	Cefprozil (2nd)	389	478	133	27.8	−0.06	−3.71	4/6	5
19	Cefamandole (2nd)	463	541	151	27.9	−0.30	−3.83	3/8	7
20	Cefonicid (2nd)	543	611	205	33.6	−1.15	−7.00	4/11	9

Table S5. *Cont.*

Entry	Name	Mw	MSA	PSA	%PSA	ClogP	$ClogD_{7.4}$	HD/HA	RB
21	Cefuroxime (2nd)	424	495	174	35.2	−1.44	−5.01	3/7	8
22	Cefoxin (2nd)	464	561	212	37.8	−2.54	−8.30	5/9	11
23	Cefotetan (2nd)	576	606	220	36.3	−0.87	−7.64	4/11	9
24	Flomoxef (2nd)	496	578	169	29.2	−0.67	−4.22	3/10	11
25	Cefminox (2nd)	520	617	203	32.9	−1.84	−7.68	4/11	11
26	Cefotiam (2nd)	526	642	172	26.8	−0.71	−5.18	3/10	10
27	Cefmetazole (2nd)	472	552	163	29.5	−1.06	−4.60	2/9	9
28	Cefuzonam (2nd)	514	545	173	31.7	−0.58	−4.22	3/10	8
29	Cefbuperazone (2nd)	614	748	240	32.1	−2.71	−6.25	5/12	9
30	Ceforanide (2nd)	520	620	194	31.3	−0.91	−9.83	4/10	10

Table S6. Physical properties of cephalosporins (3rd–5th generations).

Entry	Name	Mw	MSA	PSA	%PSA	ClogP	$ClogD_{7.4}$	HD/HA	RB
1	Ceftizoxime (3rd)	383	422	147	34.8	−0.52	−4.09	3/8	5
2	Cefetamet (3rd)	397	452	147	32.5	−0.36	−3.92	3/8	5
3	Cefpodoxime (3rd)	427	498	156	31.3	−1.09	−4.64	3/9	7
4	Cefdaloxime (3rd)	413	463	167	36.1	−1.82	−7.80	4/9	6
5	Cefditroren (3rd)	507	565	160	28.3	0.31	−3.26	3/9	7
6	Cefixime (3rd)	453	488	185	37.9	−0.64	−7.69	4/10	8
7	Cefdinir (3rd)	395	415	158	38.1	−0.91	−6.89	4/8	5
8	Cefotaxime (3rd)	455	524	174	33.2	−1.24	−4.80	3/9	8
9	Ceftibuten (3rd)	410	442	163	36.9	−0.48	−7.15	4/8	6
10	Cefcapene (3rd)	453	527	178	33.8	−0.24	−3.76	4/7	8
11	Cefmenoxime (3rd)	512	568	191	33.6	−0.40	−3.99	3/11	8
12	Cefpiramide (3rd)	613	703	209	29.7	−0.41	−3.96	5/11	9
13	Cefoperazone (3rd)	646	784	220	28.1	−1.11	−4.63	4/11	9
14	Cefteram (3rd)	479	544	191	35.1	−0.38	−3.98	3/11	7
15	Cefodizime (3rd)	585	654	197	30.1	0.71	−6.03	4/11	10
16	Ceftiolene (3rd)	595	626	226	36.1	−1.50	−5.93	4/12	10
17	Ceftriaxone (3rd)	555	604	209	34.6	−1.02	−5.70	4/12	8
18	Ceftazime (3rd)	547	660	191	28.9	−3.70	−7.54	3/10	9
19	Cefpimizole (3rd)	671	812	243	29.9	−6.09	−11.21	5/11	12
20	Cefepime (4th)	481	610	150	24.6	−5.19	−4.14	2/8	7
21	Cefquinome (4th)	529	656	154	23.5	−3.46	−3.83	2/8	7
22	Cefpirome (4th)	515	624	154	24.7	−3.85	−4.22	2/8	7
23	Cefozopran (4th)	515	594	171	28.8	−4.05	−4.42	2/10	7
24	Ceftobiprole (5th)	535	606	203	33.5	−2.26	−9.23	5/11	6

Table S7. Physical properties of tetracyclines.

Entry	Name	Mw	MSA	PSA	%PSA	ClogP	ClogD$_{7.4}$	HD/HA	RB
1	Tetracycline	444	562	182	32.4	−2.16	−7.39	6/9	2
2	Doxycycline	444	557	182	32.7	−1.81	−7.00	6/9	2
3	Oxytetracycline	460	570	202	35.4	−3.23	−8.56	7/10	2
4	Metacycline	442	531	182	34.3	−1.79	−7.08	6/9	2
5	Chlortetracycline	479	576	182	31.6	−1.64	−7.16	6/9	2
6	Minocycline	457	609	165	27.1	−0.83	−5.90	5/9	3
7	Demeclocycline	465	542	182	33.6	−1.72	−7.28	6/9	2
8	PTK 0796	557	821	177	21.6	0.41	−4.78	6/10	7
9	Rolitetracycline	528	716	171	23.9	−1.24	−6.42	6/10	4
10	Tigecycline	586	836	206	24.6	−1.17	−6.36	7/11	7
11	Lymecycline	603	828	243	29.3	−2.08	−9.40	9/13	10

Table S8. Physical properties of sulfa drugs.

Entry	Name	Mw	MSA	PSA	%PSA	ClogP	ClogD$_{7.4}$	HD/HA	RB
1	Sulfadiazine	250	321	98.0	30.5	0.37	−0.11	2/5	2
2	Sulfamerazine	264	353	98.0	27.8	0.54	0.06	2/5	2
3	Sulfadimidine	278	386	98.0	25.4	0.71	0.24	2/5	2
4	Sulfametoxydiazine	280	369	107	29.0	0.12	−0.32	2/6	3
5	Sulfachlorpyradizine	284	337	98.0	29.1	0.77	0.07	2/5	2
6	Sulfamethoxypyridazine	280	368	107	29.1	0.33	−0.24	2/6	3
7	Sulfadimethoxine	310	417	116	27.8	1.26	0.74	2/7	4
8	Sulfamonomethoxine	280	369	107	29.0	0.84	0.44	2/6	3
9	Sulfapyridine	249	332	85.1	25.6	1.21	1.18	2/4	2
10	Sulfanitran	335	441	121	27.4	1.89	1.70	2/5	4
11	Sulfaquinoxaline	300	384	98.0	25.5	1.70	1.10	2/5	2
12	Sulfaphenazole	314	413	90.0	21.8	1.72	1.19	2/4	3
13	Sulfathiazole	255	308	85.1	27.6	1.08	0.05	2/4	2
14	Sulfamethizole	270	332	98.0	29.5	0.03	−1.05	2/5	2
15	Sulfamethoxazole	253	333	98.2	29.5	1.04	0.13	2/4	2
16	Sulfafurazole	267	365	98.2	26.9	0.91	−0.11	2/4	2
17	Sulfaguanidine	214	277	125	45.1	−0.06	−0.06	3/6	1
18	Sulfacetamide	214	286	89.3	31.2	−0.11	−1.24	2/4	1
19	Sulfabenzamide	276	362	89.3	24.7	1.63	0.49	2/4	2

Table S9. Physical properties of fluoroquinolones.

Entry	Name	Mw	MSA	PSA	%PSA	ClogP	ClogD$_{7.4}$	HD/HA	RB
1	Flumequine	261	328	57.6	17.6	2.26	0.85	1/4	1
2	Nalidixic acid	232	313	70.5	22.5	1.22	−0.27	1/5	2
3	Oxolinic acid	261	326	76.1	23.3	1.35	−0.47	1/6	2
4	Piromidic acid	288	394	86.6	22.0	1.50	−0.29	1/7	3
5	Pipemidic acid	303	415	98.7	23.8	0.62	−2.66	2/8	3
6	Rosoxacin	294	388	70.5	18.2	2.04	0.77	1/5	3
**7**	**Ciprofloxacin**	**331**	**441**	**72.9**	**16.5**	**1.57**	**−1.38**	**2/6**	**3**
8	Enoxacin	320	429	85.8	20.0	1.60	−1.51	2/7	3
9	Fleroxacin	369	478	64.1	13.4	1.83	0.11	1/6	4
10	Lomefloxacin	365	501	72.9	14.6	2.53	−0.42	2/6	3
11	Nadifloxacin	360	483	81.1	16.8	1.77	0.48	2/6	2
12	Norfloxacin	319	436	72.9	16.7	1.51	−1.42	2/6	3
13	Ofloxacin	361	485	73.3	15.1	1.51	−0.33	1/7	2
14	Rufloxacin	363	462	64.1	13.9	1.76	−0.10	1/6	2
15	Pefloxacin	333	468	64.1	13.7	1.87	0.20	1/6	3
16	Balofloxacin	389	548	82.1	15.0	1.89	−1.97	2/7	5
17	Grepafloxacin	359	500	72.9	14.6	2.45	−0.47	2/6	3
18	Pazufloxacin	318	405	92.9	22.9	0.70	−2.09	2/6	2
19	Sparfloxacin	392	517	98.9	19.1	2.40	−0.66	3/7	3
20	Temafloxacin	417	523	72.9	13.9	3.54	0.37	2/6	3
21	Tosufloxacin	404	483	99.8	20.7	2.93	−1.23	2/7	3
22	Clinafloxacin	365	452	86.9	19.2	1.80	−2.15	2/6	3
23	Gatifloxacin	375	517	82.1	15.9	1.73	−1.33	2/7	4
24	Gemifloxacin	389	510	121	23.8	2.09	−1.89	2/9	5
25	Moxifloxacin	401	549	82.1	15.0	1.85	−1.88	2/7	4
26	Sitafloxacin	410	497	86.9	17.5	2.18	−1.48	2/6	3
27	DX-619	401	552	96.1	17.4	0.92	−2.87	2/7	5
28	Danofloxacin	357	474	64.1	13.5	1.94	0.27	1/6	3
29	Garenoxacin	426	543	78.9	14.5	3.68	0.26	2/6	5
30	Trovafloxacin	416	487	99.8	20.5	2.69	−1.32	2/7	3
31	Difloxacin	399	519	64.1	12.4	3.35	1.65	1/6	3
32	ABT-492	441	473	120	25.4	2.66	0.88	3/8	3

Table S10. Physical properties of aminoglycosides.

Entry	Name	Mw	MSA	PSA	%PSA	ClogP	$ClogD_{7.4}$	HD/HA	RB
1	Gentamicin	494	773	234	30.3	−5.03	−13.35	9/13	7
2	Kanamycin	484	680	283	41.6	−7.73	−13.39	11/15	6
3	Netilmicin	476	745	200	26.8	−4.74	−12.33	8/12	8
4	Sisomicin	448	678	214	31.6	−5.49	−13.16	8/12	6
5	Ribostamycin	454	641	262	40.9	−7.06	−12.45	10/14	6
6	Amikacin	586	826	332	40.2	−9.31	−15.88	13/17	10
7	Arbekacin	553	817	297	36.4	−7.51	−16.35	11/15	10
8	Tobramycin	468	681	268	39.4	−7.06	−14.27	10/14	6
9	Framycetin	615	866	353	40.8	−9.27	−16.87	13/19	9
10	Streptomycin	582	783	336	42.9	−6.99	−12.19	12/19	9
11	Isepamicin	570	825	298	36.1	−8.20	−14.10	12/16	9
12	Paromomycin	616	859	347	40.4	−9.23	−15.41	13/19	9
13	Neomycin C	615	870	353	40.6	−9.27	−16.87	13/19	9

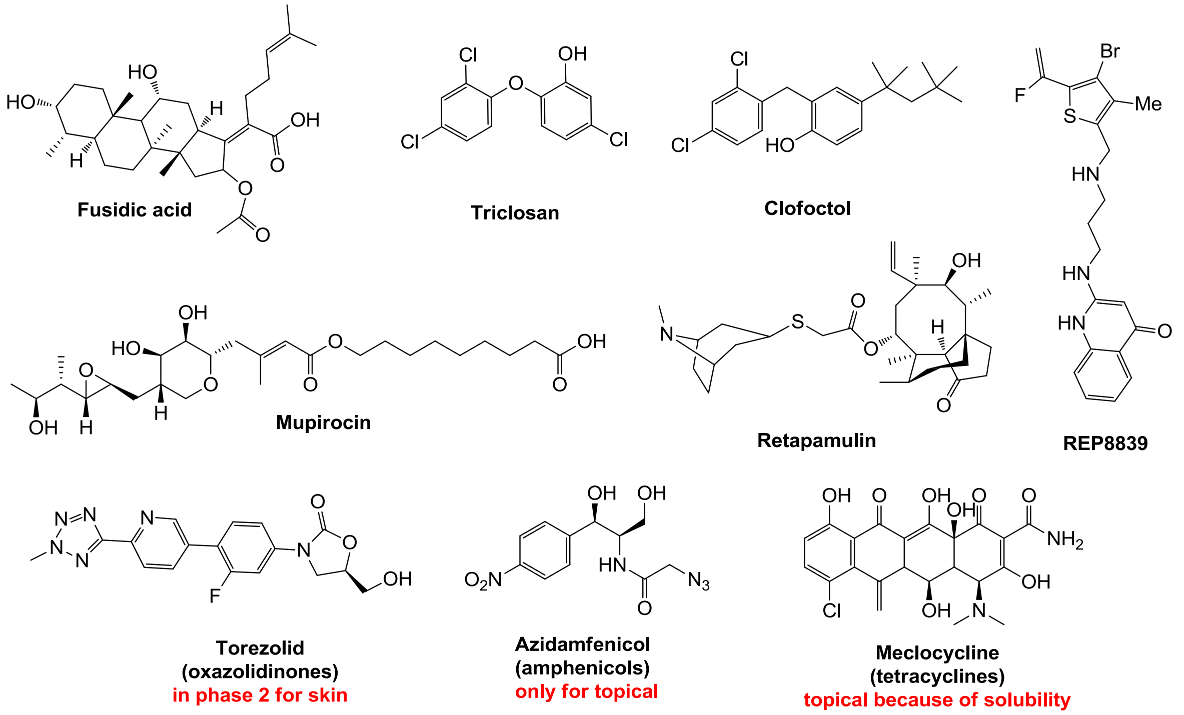

Figure S6. Structures of topical antibiotics.

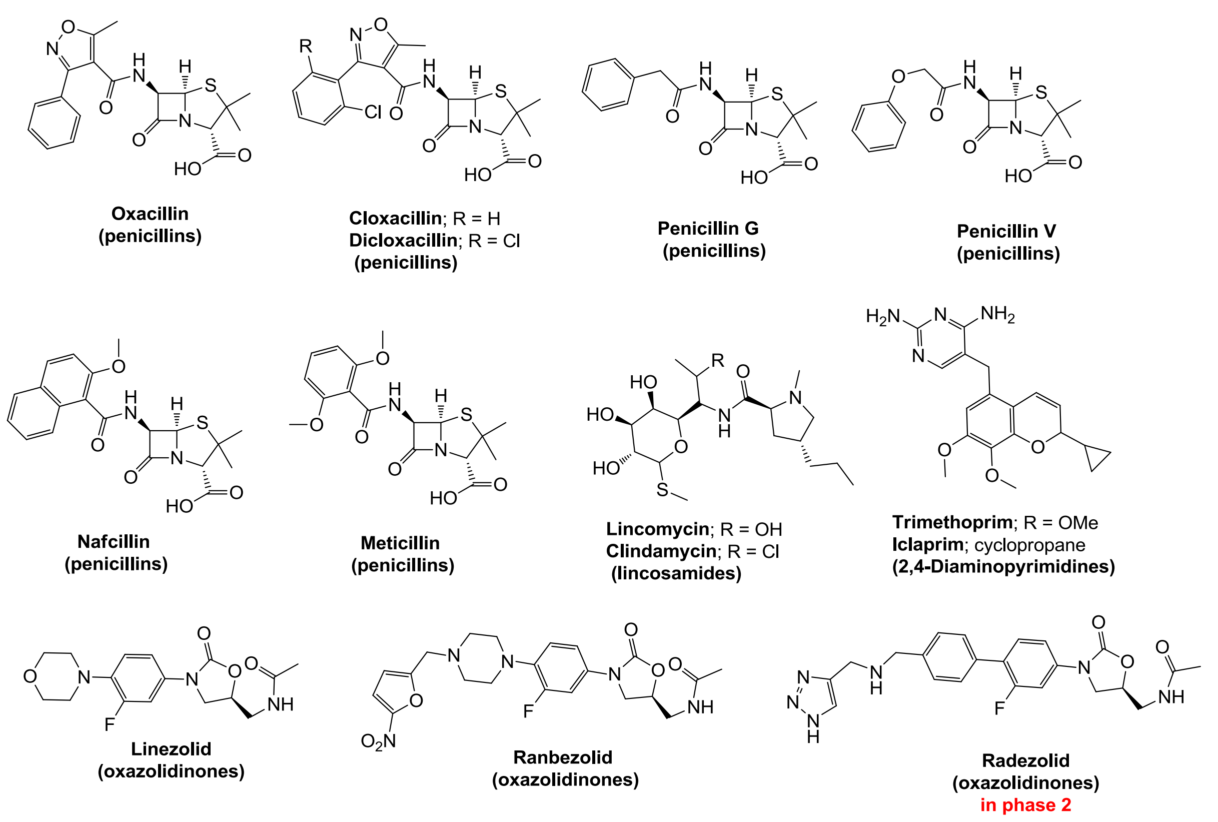

Figure S7. Gram-positive only active penicillins, lincosamides and oxazolidinones.

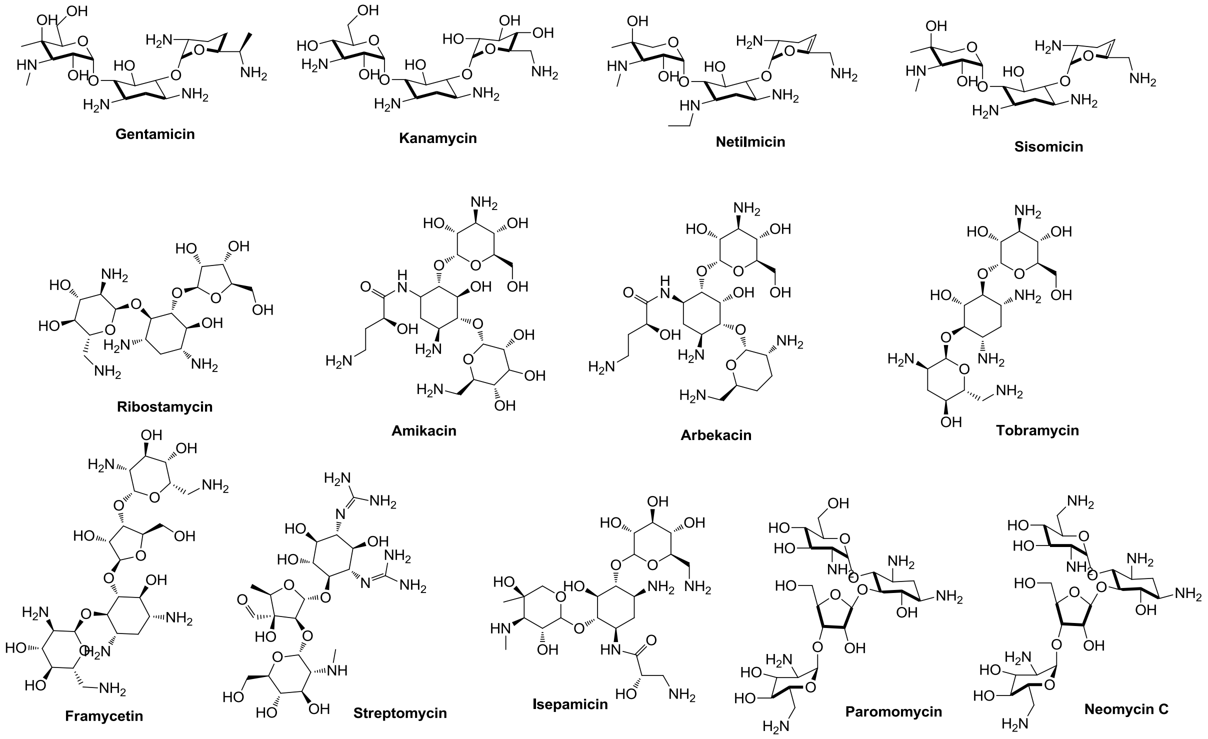

Figure S8. Structures of aminoglycosides.

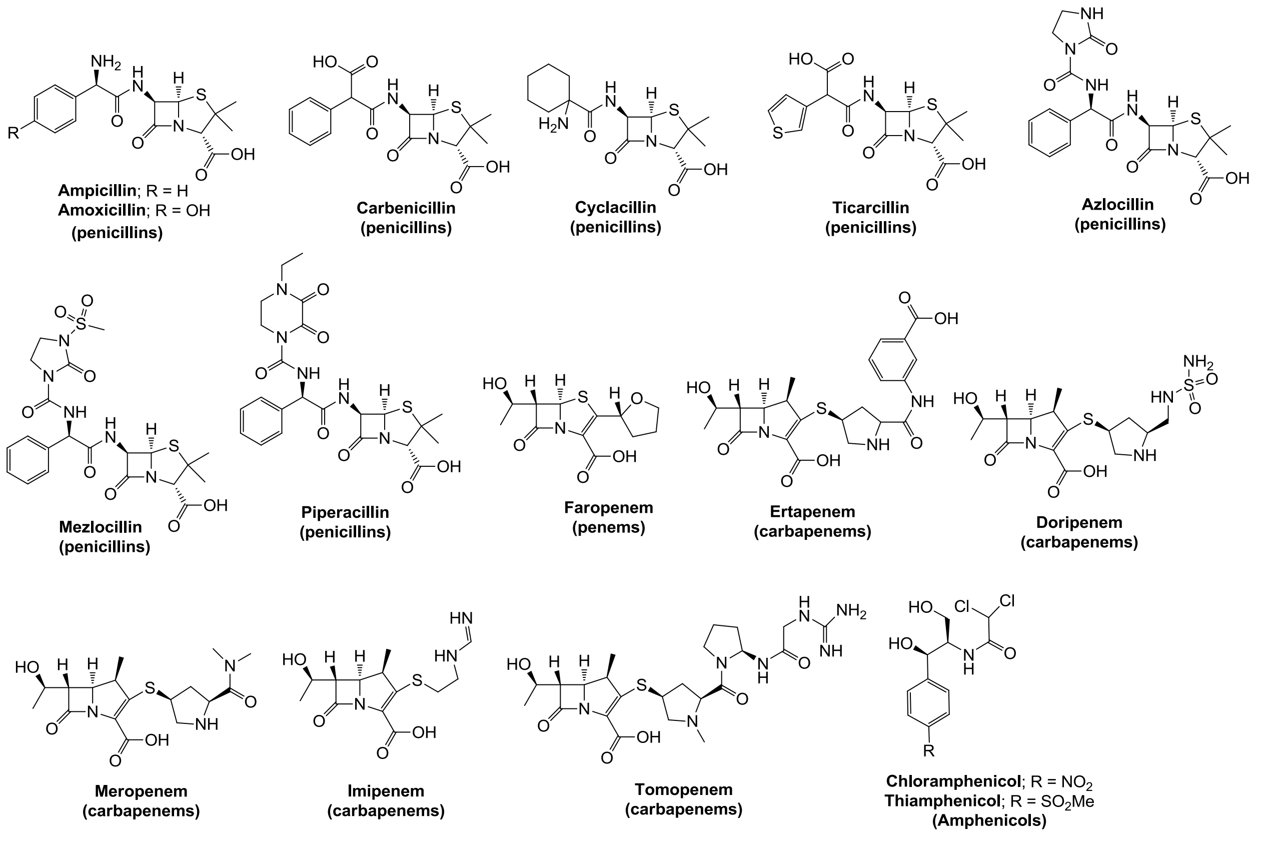

Figure S9. Structures of Gram positive and negative penicillins and amphenicols.

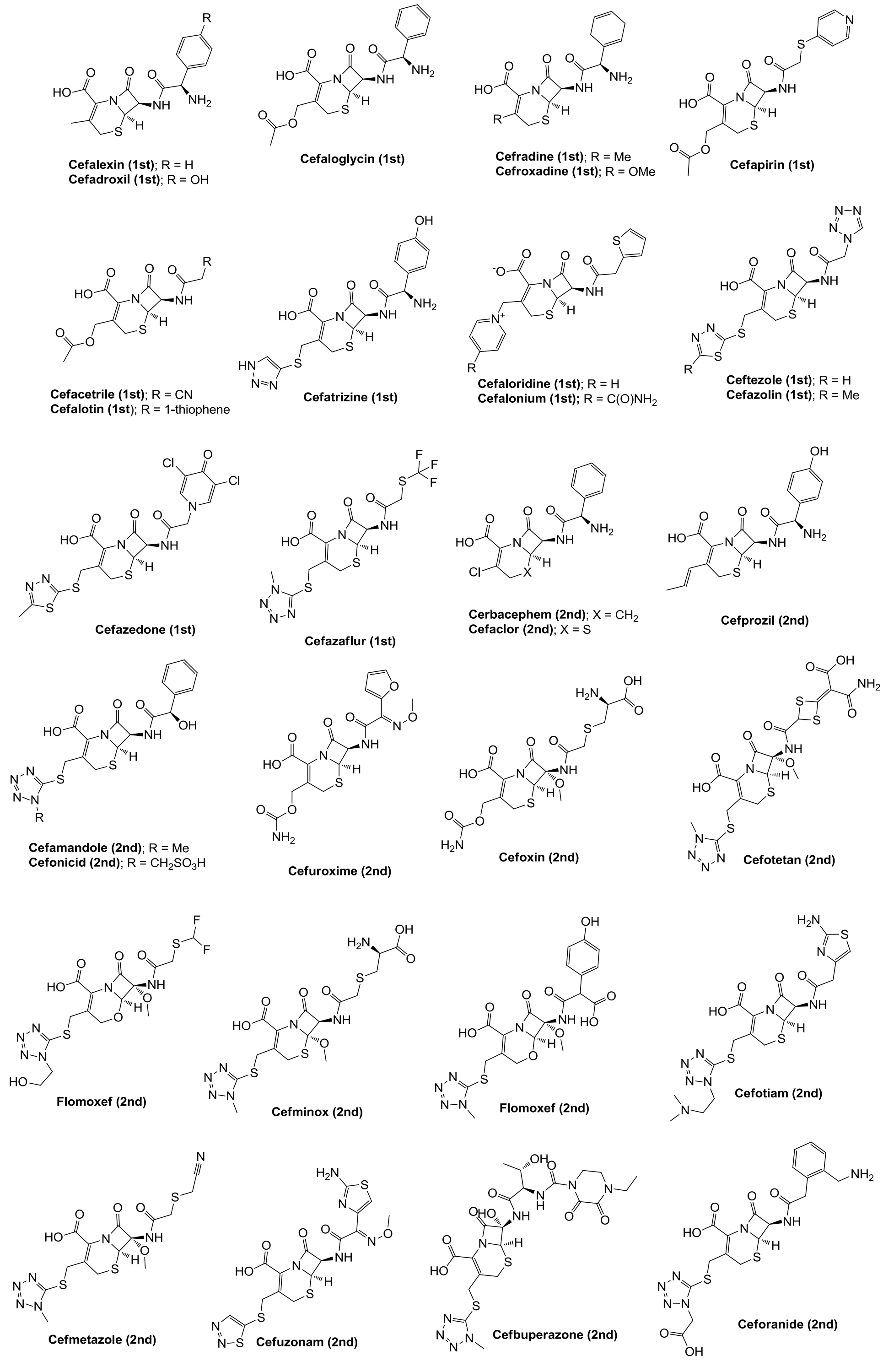

Figure S10. Structures of Cephalosporin antibiotics (1st and 2nd generations).

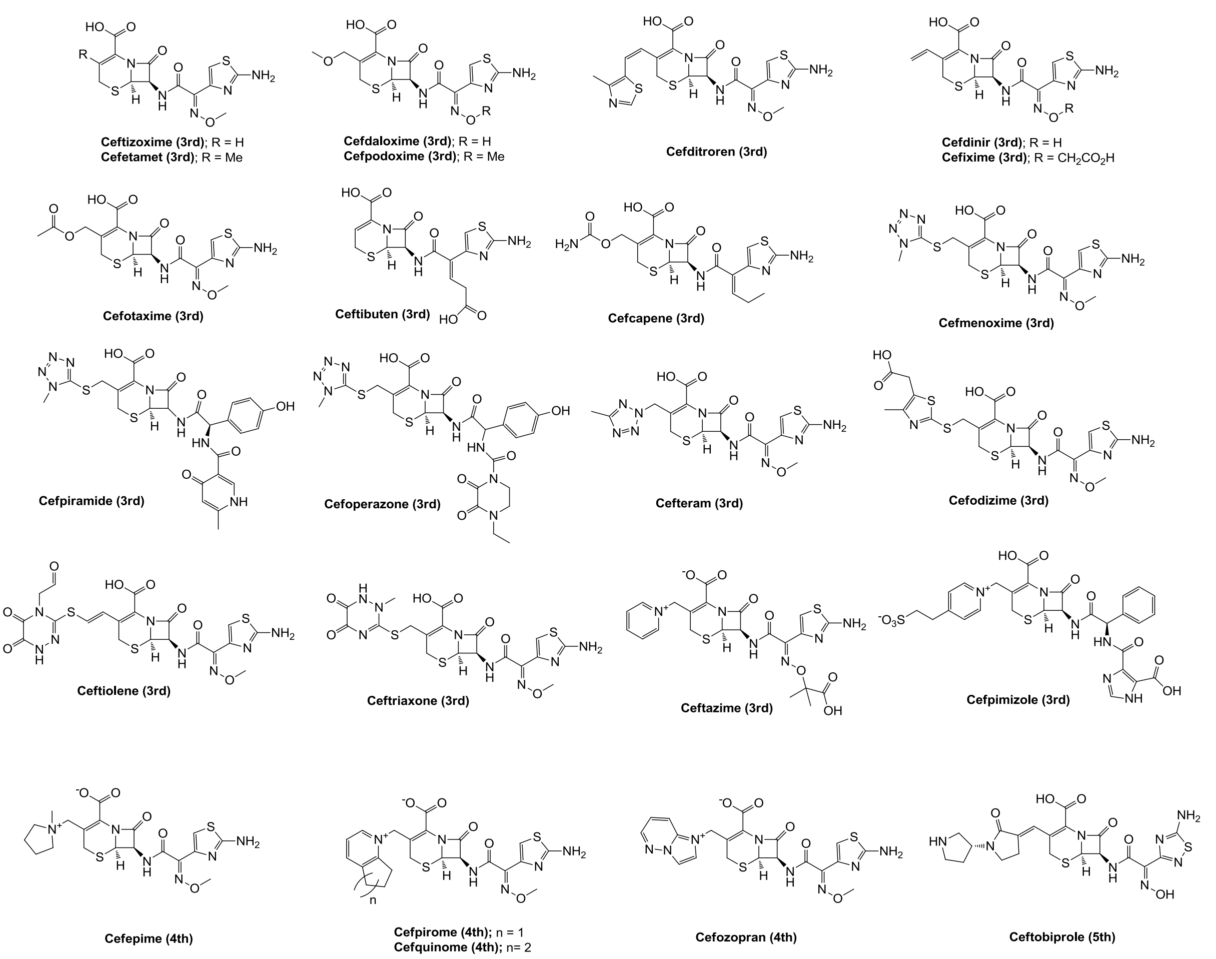

Figure S11. Structures of Cephalosporin antibiotics (3rd–5th generations).

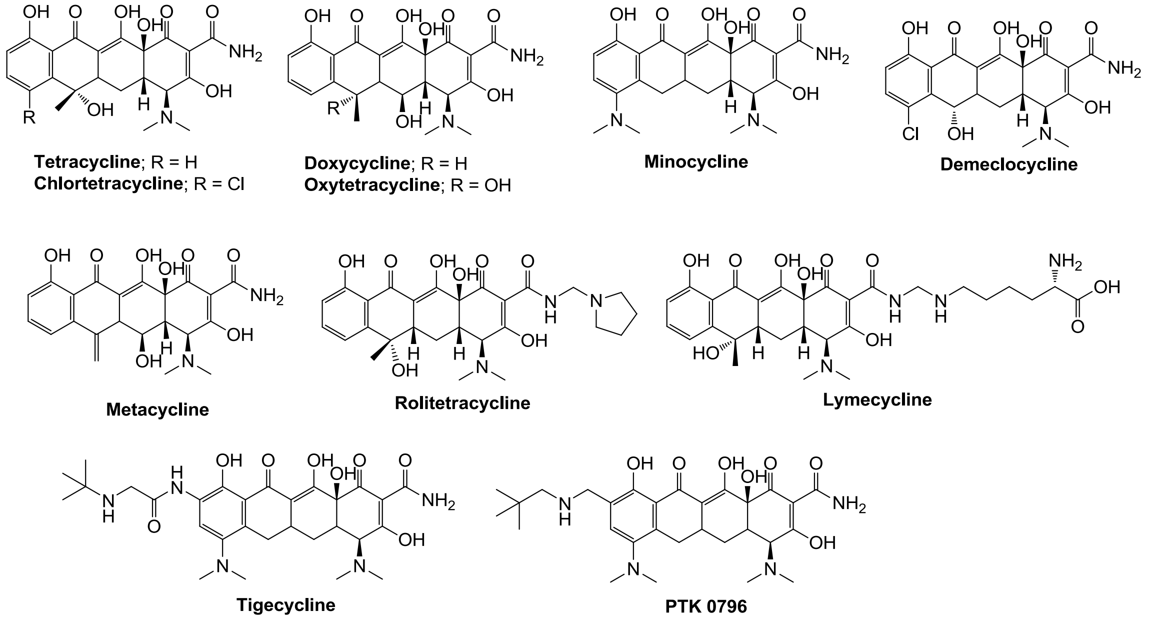

Figure S12. Structures of tetracyclines.

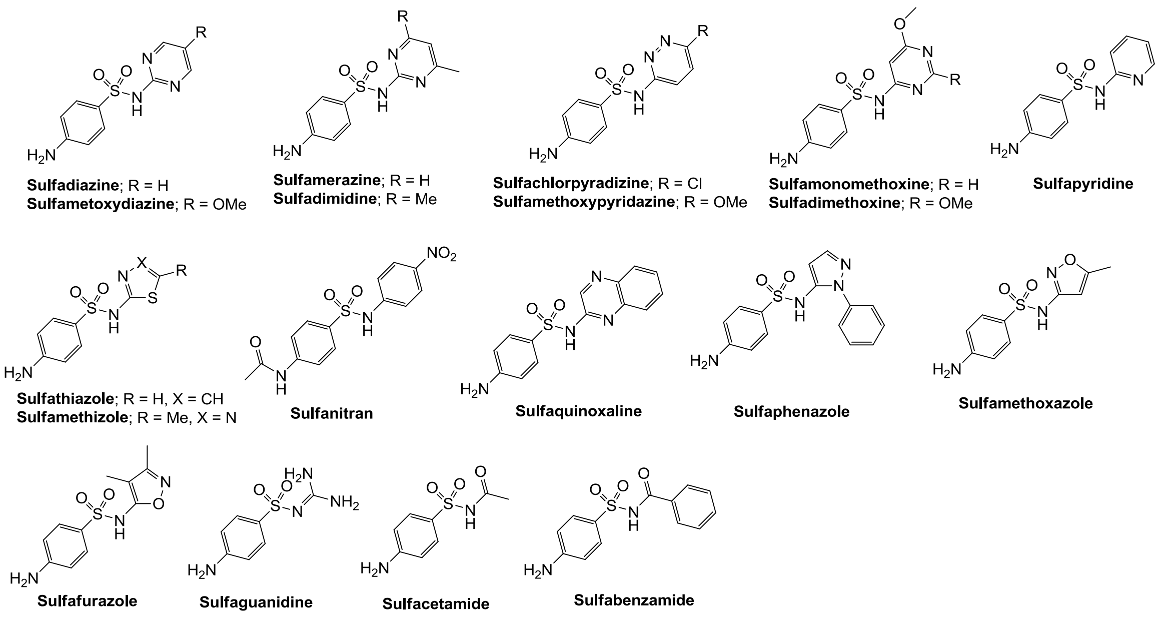

Figure S13. Structures of sulfa drugs.

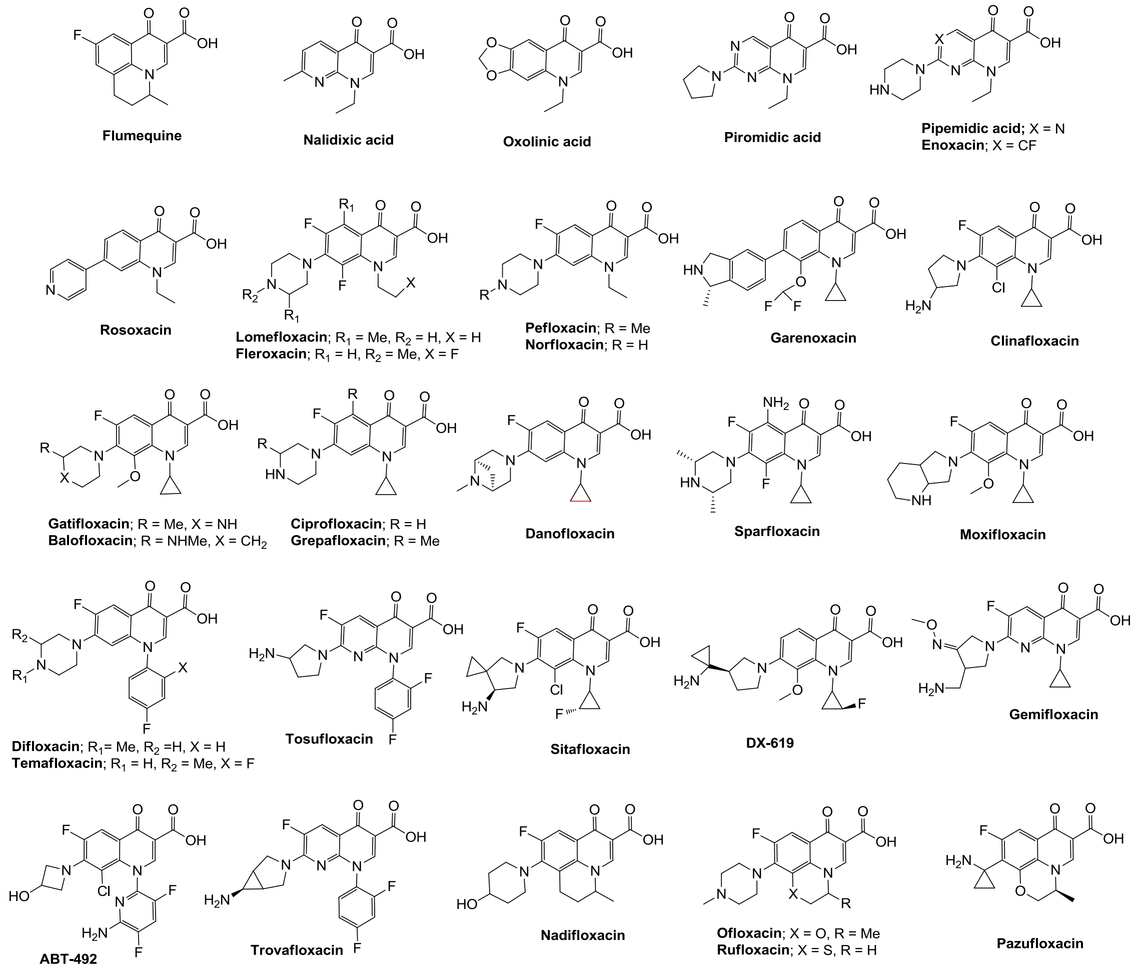

Figure S14. Structures of fluoroquinolones.

Spartan Calculation Data

2a endo-enol

Spartan '02 Mechanics Program: (PC/×86) Release 115B
Reason for exit: Successful completion
Mechanics CPU Time : 000:00:00.3
Mechanics Wall Time: 000:00:00.3
Spartan "02 Quantum Mechanics Program: (PC/x86) Release 115B
To use a standard Psuedopotential a heavy atom must exists
(An atom larger than 'Ar')

Job type: Geometry optimization.
Method: RB3LYP
Basis set: LACVP*
Number of shells: 104
Number of basis functions: 230

SCF model:
A restricted hybrid HF-DFT SCF calculation will be
performed using Pulay DIIS extrapolation

Optimization:

Step	Energy	Max Grad.	Max Dist.
1	−721.3188486	0.033624	0.078653
2	−721.3308940	0.016086	0.127985
3	−721.3333004	0.012471	0.103943
4	−721.3342245	0.007061	0.135359
5	−721.3346190	0.002968	0.210012
6	−721.3347204	0.004707	0.157507
7	−721.3348419	0.002430	0.218716
8	−721.3348432	0.002043	0.134983
9	−721.3348046	0.005487	0.083404
10	−721.3349435	0.001104	0.029607
11	−721.3349530	0.000264	0.013534
12	−721.3349531	0.000214	0.004257

Reason for exit: Sucessful completion
Quantum Mechanics Program CPU Time : 001:13:59.5
Quantum Mechanics Program Wall Time: 001:14:40.5

Spartan '02 Properties Program: (PC/×86) Release 115B
Reason for exit: Successful completion
Properties Program CPU Time : 000:00:00.6
Properties Program Wall Time: 000:00:00.7

2a exo-enol

Spartan '02 Mechanics Program: (PC/×86) Release 115B
Reason for exit: Successful completion
Mechanics CPU Time : 000:00:00.2
Mechanics Wall Time: 000:00:00.2
Spartan '02 Quantum Mechanics Program: (PC/x86) Release 115B
To use a standard Psuedopotential a heavy atom must exists

(An atom larger than 'Ar')

Job type: Geometry optimization.
Method: RB3LYP
Basis set: LACVP*
Number of shells: 104
Number of basis functions: 230

SCF model:
A restricted hybrid HF-DFT SCF calculation will be performed using Pulay DIIS extrapolation

Optimization:

Step	Energy	Max Grad.	Max Dist.	
1	−721.3263787	0.032326	0.113498	1
2	−721.3370556	0.018469	0.126549	1
3	−721.3383956	0.006959	0.047221	1
4	−721.3387449	0.004516	0.204974	1
5	−721.3389192	0.002930	0.190566	1
6	−721.3388968	0.001947	0.207503	
7	−721.3389925	0.003011	0.086110	
8	−721.3389887	0.002448	0.173256	
9	−721.3390866	0.002429	0.123832	
10	−721.3390916	0.000901	0.090482	
11	−721.3391152	0.000999	0.015727	
12	−721.3391209	0.001146	0.018765	
13	−721.3391254	0.000996	0.024971	
14	−721.3391220	0.000984	0.013254	
15	−721.3391225	0.000908	0.015705	
16	−721.3391255	0.000908	0.025376	
17	−721.3391308	0.000868	0.045755	
18	−721.3391351	0.000957	0.078654	
19	−721.3391434	0.001057	0.106313	
20	−721.3391527	0.000909	0.111160	
21	−721.3391706	0.000368	0.052632	
22	−721.3391826	0.000266	0.007876	
23	−721.3391804	0.000125	0.008678	
24	−721.3391816	0.000022	0.002365	
25	−721.3391832	0.000011	0.000781	

Reason for exit: Sucessful completion
Quantum Mechanics Program CPU Time : 002:14:01.3
Quantum Mechanics Program Wall Time: 002:15:10.1

Spartan '02 Properties Program: (PC/×86) Release 115B
Reason for exit: Successful completion
Properties Program CPU Time : 000:00:00.6

43a endo-enol
Spartan '02 Mechanics Program: (PC/×86) Release 115B
Reason for exit: Successful completion
Mechanics CPU Time : 000:00:00.3
Mechanics Wall Time: 000:00:00.4

Spartan '02 Quantum Mechanics Program: (PC/×86) Release 115B
To use a standard Psuedopotential a heavy atom must exists
(An atom larger than 'Ar')

Job type: Geometry optimization.
Method: RB3LYP
Basis set: LACVP*
Number of shells: 112
Number of basis functions: 247

SCF model:
A restricted hybrid HF-DFT SCF calculation will be
performed using Pulay DIIS extrapolation

Optimization:

Step	Energy	Max Grad.	Max Dist.
1	-776.6951997	0.046908	0.096893
2	-776.7079966	0.019827	0.037601
3	-776.7096040	0.006526	0.024911
4	-776.7100745	0.003761	0.010782
5	-776.7101654	0.001752	0.009819
6	-776.7102003	0.000709	0.005156
7	-776.7102072	0.000187	0.000557

Reason for exit: Sucessful completion
Quantum Mechanics Program CPU Time : 000:51:47.3
Quantum Mechanics Program Wall Time: 000:52:13.6

Spartan '02 Properties Program: (PC/×86) Release 115B
Orientation rotated by 179.999994991044 degrees!
Reason for exit: Successful completion
Properties Program CPU Time : 000:00:00.8
Properties Program Wall Time: 000:00:00.8

43a exo-enol
Spartan '02 Mechanics Program: (PC/×86) Release 115B
Reason for exit: Successful completion
Mechanics CPU Time : 000:00:00.2
Mechanics Wall Time: 000:00:00.3
Spartan '02 Quantum Mechanics Program: (PC/x86) Release 115B
To use a standard Psuedopotential a heavy atom must exists
(An atom larger than 'Ar')
Job type: Geometry optimization.
Method: RB3LYP
Basis set: LACVP*
Number of shells: 112
Number of basis functions: 247

SCF model:
A restricted hybrid HF-DFT SCF calculation will be
performed using Pulay DIIS extrapolation

Optimization:

Step	Energy	Max Grad.	Max Dist.
1	−776.6966514	0.046695	0.131846
2	−776.7110719	0.015006	0.044762
3	−776.7129486	0.006951	0.021776
4	−776.7132746	0.002265	0.005289
5	−776.7133219	0.000872	0.003559
6	−776.7133319	0.000303	0.001371
7	−776.7133347	0.000075	0.000230

Reason for exit: Sucessful completion
Quantum Mechanics Program CPU Time : 000:49:17.3
Quantum Mechanics Program Wall Time: 000:49:38.5

Spartan '02 Properties Program: (PC/×86) Release 115B
Orientation rotated by 179.999994991044 degrees!
Reason for exit: Successful completion
Properties Program CPU Time : 000:00:00.8
Properties Program Wall Time: 000:00:00.8

43b endo-enol
Spartan '02 Mechanics Program: (PC/×86) Release 115B
Reason for exit: Successful completion
Mechanics CPU Time : 000:00:00.3
Mechanics Wall Time: 000:00:00.3
Spartan '02 Quantum Mechanics Program: (PC/×86) Release 115B
To use a standard Psuedopotential a heavy atom must exists
(An atom larger than 'Ar')

Job type: Geometry optimization.
Method: RB3LYP
Basis set: LACVP*
Number of shells: 110
Number of basis functions: 245

SCF model:
A restricted hybrid HF-DFT SCF calculation will be
performed using Pulay DIIS extrapolation

Optimization:

Step	Energy	Max Grad.	Max Dist.
1	−796.5398353	0.031101	0.081303
2	−796.5514058	0.011737	0.115368
3	−796.5539497	0.009158	0.125261
4	−796.5553533	0.002656	0.200584
5	−796.5556053	0.002332	0.171176
6	−796.5557240	0.002477	0.189063
7	−796.5557514	0.002169	0.040229
8	−796.5557948	0.001725	0.046947
9	−796.5557987	0.001456	0.027589
10	−796.5558121	0.000259	0.009347
11	−796.5558110	0.000136	0.006829
12	−796.5558111	0.000094	0.013612

Reason for exit: Sucessful completion
Quantum Mechanics Program CPU Time : 001:18:46.1
Quantum Mechanics Program Wall Time: 001:19:35.8

Spartan '02 Properties Program: (PC/×86) Release 115B
Reason for exit: Successful completion
Properties Program CPU Time : 000:00:00.8
Properties Program Wall Time: 000:00:00.9

43b exo-enol
Spartan '02 Mechanics Program: (PC/×86) Release 115B
Reason for exit: Successful completion
Mechanics CPU Time : 000:00:00.2
Mechanics Wall Time: 000:00:00.2
Spartan '02 Quantum Mechanics Program: (PC/x86) Release 115B
To use a standard Psuedopotential a heavy atom must exists
(An atom larger than 'Ar')

Job type: Geometry optimization.
Method: RB3LYP
Basis set: LACVP*
Number of shells: 110
Number of basis functions: 245

SCF model:
A restricted hybrid HF-DFT SCF calculation will be
performed using Pulay DIIS extrapolation

Optimization:

Step	Energy	Max Grad.	Max Dist.
1	−796.5309818	0.040547	0.109151
2	−796.5474243	0.018759	0.126703
3	−796.5497678	0.010188	0.115629
4	−796.5505489	0.009241	0.102296
5	−796.5513235	0.003616	0.199455
6	−796.5514271	0.003953	0.134447
7	−796.5516225	0.003191	0.161288
8	−796.5515938	0.002848	0.231051
9	−796.5517480	0.002009	0.157874
10	−796.5517238	0.002433	0.209888
11	−796.5518567	0.001892	0.110813
12	−796.5518573	0.001583	0.125314
13	−796.5518868	0.000898	0.043009
14	−796.5518925	0.000637	0.020466
15	−796.5518905	0.000204	0.012587
16	−796.5518938	0.000154	0.011356
17	−796.5518937	0.000140	0.002354

Reason for exit: Sucessful completion
Quantum Mechanics Program CPU Time: 001:55:05.9
Quantum Mechanics Program Wall Time: 001:56:02.5

Spartan '02 Properties Program: (PC/×86) Release 115B
Reason for exit: Successful completion
Properties Program CPU Time : 000:00:00.8
Properties Program Wall Time: 000:00:00.8

43c endo-enol

Spartan '02 Mechanics Program: (PC/×86) Release 115B
Reason for exit: Successful completion
Mechanics CPU Time : 000:00:00.2
Mechanics Wall Time: 000:00:00.3
Spartan '02 Quantum Mechanics Program: (PC/x86) Release 115B
To use a standard Psuedopotential a heavy atom must exists
(An atom larger than 'Ar')

Job type: Geometry optimization.
Method: RB3LYP
Basis set: LACVP*
Number of shells: 116
Number of basis functions: 251

SCF model:
A restricted hybrid HF-DFT SCF calculation will be
performed using Pulay DIIS extrapolation

Optimization:

Step	Energy	Max Grad.	Max Dist.
1	−740.7411033	0.027986	0.138068
2	−740.7531158	0.012369	0.160457
3	−740.7572300	0.010632	0.160369
4	−740.7598000	0.008894	0.133589
5	−740.7612865	0.010822	0.166581
6	−740.7622362	0.013628	0.131936
7	−740.7648297	0.016571	0.170287
8	−740.7678508	0.072216	0.181196
9	−740.7661919	0.123149	0.094622
10	−740.7742026	0.029218	0.081418
11	−740.7764886	0.033657	0.163726
12	−740.7787096	0.012189	0.206396
13	−740.7787037	0.114526	0.096824
14	−740.7785679	0.085077	0.112683
15	−740.7799352	0.014139	0.048742
16	−740.7802972	0.009886	0.124529
17	−740.7804272	0.001948	0.182666
18	−740.7804337	0.011901	0.143527
19	−740.7805176	0.011679	0.042119
20	−740.7805533	0.003066	0.104964
21	−740.7806003	0.003279	0.161397
22	−740.7806736	0.001733	0.168561
23	−740.7807771	0.003138	0.134783
24	−740.7808980	0.006986	0.119081
25	−740.7810368	0.008778	0.160896
26	−740.7811143	0.004827	0.054200

27	−740.7811699	0.006834	0.061373
28	−740.7811908	0.000564	0.013420
29	−740.7811953	0.000140	0.010785
30	−740.7811978	0.000619	0.007744
31	−740.7811998	0.000120	0.004211
32	−740.7811964	0.000032	0.000856

Reason for exit: Sucessful completion
Quantum Mechanics Program CPU Time : 003:49:54.6
Quantum Mechanics Program Wall Time: 003:51:52.6

Spartan '02 Properties Program: (PC/x86) Release 115B
Reason for exit: Successful completion
Properties Program CPU Time : 000:00:00.8
Properties Program Wall Time: 000:00:00.9

43c exo-enol
Spartan '02 Mechanics Program: (PC/×86) Release 115B
Reason for exit: Successful completion
Mechanics CPU Time : 000:00:00.3
Mechanics Wall Time: 000:00:00.3
Spartan '02 Quantum Mechanics Program: (PC/×86) Release 115B
To use a standard Psuedopotential a heavy atom must exists
(An atom larger than 'Ar')

Job type: Geometry optimization.
Method: RB3LYP
Basis set: LACVP*
Number of shells: 116
Number of basis functions: 251

SCF model:
A restricted hybrid HF-DFT SCF calculation will be
performed using Pulay DIIS extrapolation

Optimization:

Step	Energy	Max Grad.	Max Dist.	
1	−740.7654588	0.030768	0.109693	1
2	−740.7768503	0.009971	0.107683	1
3	−740.7791640	0.005401	0.122953	1
4	−740.7799392	0.001780	0.144484	1
5	−740.7802953	0.002154	0.137447	
6	−740.7805198	0.002010	0.146240	
7	−740.7806576	0.001639	0.176762	
8	−740.7807983	0.002028	0.182191	
9	−740.7809267	0.002555	0.208698	
10	−740.7810108	0.003344	0.209505	
11	−740.7811067	0.002266	0.174507	
12	−740.7811445	0.001757	0.092090	
13	−740.7811758	0.000606	0.054986	
14	−740.7811757	0.001023	0.034879	
15	−740.7811867	0.000547	0.014782	

16	−740.7811922	0.000398	0.019310
17	−740.7811903	0.000193	0.009492
18	−740.7811931	0.000158	0.007443
19	−740.7811914	0.000093	0.002956
20	−740.7811951	0.000034	0.001365
21	−740.7811955	0.000007	0.000780

Reason for exit: Sucessful completion
Quantum Mechanics Program CPU Time : 002:20:07.9
Quantum Mechanics Program Wall Time: 002:23:11.8

Spartan '02 Properties Program: (PC/×86) Release 115B
Reason for exit: Successful completion
Properties Program CPU Time: 000:00:00.9
Properties Program Wall Time: 000:00:01.0

References

1. Jeong, Y.-C.; Anwar, M.; Moloney, M.G.; Bikadi, Z.; Hazai, E. *Chem. Sci.*, **2013**, *4*, 1008–1015.
2. Jeong, Y.-C.; Moloney, M.G.; Bikadi, Z.; Hazai, E. *ChemMedChem* **2014**, *9*, 1826–1837.
3. Jeong, Y.-C.; Moloney, M.G. *Beilstein J. Org. Chem.* **2013**, *9*, 1899–1906.
4. Jeong, Y.-C.; Anwar, M.; Moloney, M.G.; Bikadi, Z.; Hazai, E. *Biorg. Med. Chem. Lett.* **2014**, *24*, 1901–1906.
5. Critchley, I.A.; Ochsner, U. A Recent advances in the preclinical evaluation of the topical antibacterial agent REP8839. *Curr. Opin. Chem. Biol.* **2008**, *12*, 409–417.
6. O'Shea, R.; Moser, H.E. Physicochemical properties of antibacterial compounds: Implications for drug discovery. *J. Med. Chem.* **2008**, *51*, 2871–2878.
7. Kohlhoff, S.A.; Sharma, R. Iclaprim. *Expert Opin. Investig. Drugs* **2007**, *16*, 1441–1448.
8. Othersen, O.G.; Beierlein, F.; Lanig, H.; Clark, T. Conformations and tautomers of tetracycline. *J. Phys. Chem. B* **2003**, *107*, 13743–13749.
